# Supplementary material for: Co-creating community-driven solutions and policy priorities to address antimicrobial resistance through Responsive Dialogues: A qualitative evaluation from Malawi
Source: PLOS Glob Public Health. 2026 Apr 28;6(4):e0005697. doi: 10.1371/journal.pgph.0005697 (PMC13123971; doi:10.1371/journal.pgph.0005697)
Supplement: S1 Text — (DOCX) [file pgph.0005697.s001.docx]

**Facilitator:** Alright, we are starting up. Please feel free, like I said earlier there is no right or wrong answers. You should be raising your voice a bit higher so that we can all hear each other. First of all, I would like us to know each other, what do we do on our daily basis?

**Number 3:** I stay in [community name] and I’m chicken farmer specified in local chickens

**Facilitator:** Alright, how about others, what do we do on a daily basis?

**Number 2:** I stay in [community name]. I’m a chicken farmer, I have red layers and white layers.

**Facilitator:** Alright

**Number 1:** I’m a business woman, I sell eggs, I used to have chickens but currently I don’t have.

**Facilitator:** Alright. Now we are starting up. Firstly, I would like to know, what do we know about antimicrobial resistance?

**Number 1:** The issue of antibiotic resistance mostly involves misuse of antibiotics in our chickens

**Facilitator:** Alright, what do you mean when you say they are misused?

**Number 2:** In Chicken farming most of us use antibiotics to treat our chickens, however some farmers sell or consume chickens during the withdrawal period and it’s not only chickens but some farmers sell and consume meat of different tamed animals when the animals are still in withdrawal period, some of the animals include cattle, pigs or even goats. And when people consume that meat it causes them to develop antibiotic resistance, so it has a bad effect on human health.

**Facilitator:** Alright, I think you wanted to add?

**Number 3:** I also would like to add that from what we learnt during the meetings, we learnt that some drugs that we take such as Penicillin, Amoxicillin and Bactrim may cause antimicrobial resistance if we take them without seeking a prescription from the doctor.

**Facilitator:** Alright, How would that issue affect animal or human health?

**Number 3:** I will begin by answering on human health, if you develop resistance to a particular antibiotic it would be impossible for you to recover from that antibiotic when you get sick

**Facilitator:** Alright

**Number 1:** I also just want to add that sometimes antimicrobial resistance is also caused because we don’t complete the full dose of the antibiotic

**Facilitator:** Alright. How does antimicrobial resistance affect the community?

**Number 2:** In terms of our communities we lack awareness of antimicrobial resistance as a result people develop resistance and as a result they fail to recover from the drugs and they die

**Facilitator:** Alright. Do we have any other extra ideas?

**Number 3:** I just want to add on what number 2 has said, in our communities there is no any knowledge of antimicrobial resistance as a result people in the communities are selling and consuming meat which contains antibiotics and at the end of it the whole community is highly affected.

**Facilitator:** Alright. How can we overcome or prevent the issue of antimicrobial resistance?

**Number 1:** There is need for civic education on antimicrobial resistance in our communities

**Facilitator:** Okay

**Number 4:** Just to add on what she has said, it is difficult for only a small group of us who learnt about this to teach the whole community but there is need to train more people who would go into the communities to educate people in the communities about this issue

**Facilitator:** Alright.

**Number 3:** During these meetings it wasn’t only us that participated but we also had chiefs and veterinary officers during those meetings, so there is need to provide those people with resources so that they should take the messages to the people in their communities

**Facilitator:** Okay

**Number 2:** I also want to add, in my community there was a certain illegal drug vendor who was selling antibiotics within the community and when I participated in those meetings I went to him as if I want to buy the drugs and I warned him that if doesn’t leave the village I will call the police to arrest him and he left the village. I have also encouraged my colleagues to inform the police when they see any illegal drug vendor

**Facilitator:** Alright. Now, I would like to hear about your experiences on participating in those conversation events, by the way how many times did you meet?

**Number 2:** I think we met 4times, the last day was the biggest event.

**Facilitator:** Alright, so what was your experience for participating in the conversation events?

**Number 2:** I came here to participate in the conversation events as a chicken farmer and when I was coming here I had a bad habit of misusing antibiotics to treat my chickens but after participating in those meetings I have now stopped that bad habit and sometimes I just use traditional herbs to treat my chickens rather than antibiotics

**Facilitator:** You changed from doing that bad behavior due to your participation in the conversation events?

**Number 2:** Yes, I stopped that habit after participating in the conversation events

**Facilitator:** Okay

**Number 2:** However, some people are still misusing antibiotics and most of them are big chicken companies, I’m not sure if they are aware of this issue of antimicrobial resistance because they are still into that bad habit

**Facilitator:** Okay

**Number 1:** I also want to add that when I was coming to participate in the conversation events I didn’t had any knowledge on what antimicrobial resistance is, I was one of the people who was selling my chickens when they are in their withdrawal period but after participating in these conversation events I stopped that bad habit, I no longer sell my chickens when they are in their withdrawal period.

**Facilitator:** Okay

**Number 4:** Before participating in the conversation events I used to buy drugs to treat my chickens without consulting a veterinary officer first but ever since I participated in the events I now consult a veterinary officer before treating my chickens with any drugs

**Facilitator:** Okay

**Number 3:** I also want to add, when I was coming to participate in those events I never knew what an antibiotic was but after participating in the events that’s when I gained some knowledge on what an antibiotic is and what it does, so to me it was an eye opener. And we shared new ideas with our fellow farmers.

**Facilitator:** mmh

**Number 3:** However, I feel like somehow we were left behind

**Facilitator:** mmh

**Number 3:** I’m saying we were left behind because ever since we finished with the co-creation event there has never been any communication back to answer until now. But the events were helpful and we learnt a lot. I should stop from there.

**Facilitator:** Alright. You have mostly talked about knew ideas and knowledge that you gained from the meetings, but how about in terms of the venue were the meetings were taking place, time or directions to get to the venue, things like those?

**Number 2:** We used this same venue and according to me I never had any problems with the venue and the facilitators were good, we were happy and when we were getting to the end we never wanted it to end. So, in short to me everything was good

**Facilitator:** Okay. How about others?

**Number 1:** According to I also never had any problem with the venue and in terms of the distance of course I was coming from [community name] but that wasn’t a challenge for me, and as my colleague has said everything was good and it was interesting and we made new friends whom we are still talking to up to now, we even call each other. And they were providing us with food and transport was being refunded to us.

**Facilitator:** Okay

**Number 3:** As my colleagues’ number 1 and number 2 have said, the events were indeed good and interesting there was no any problem however I feel like the final event was a repetition of the event 2. And to be honest after the final event we never received any communication and of course they mentioned that they will hold other meetings with other stakeholders but we don’t know if that was done or if they included anyone of us in those other events.

**Facilitator:** Okay. So, you are suggesting that if there was any other meeting or there is going to be another meeting they should include a representative from your group?

**Number 3:** Yes, because if that was done then the representative would have been giving us updates on what is happening. We even have a whatsapp group so they could have communicated with us through that.

**Facilitator:** Alright

**Number 4:** I should also agree with number 3, when we were concluding the events during the final event they were some points which we agreed that they are supposed to be taken to the government to be addressed but ever since we left there hasn’t been any feedback from them and the big companies sell chickens which look far different from us meaning that there is something fishy that they do to their chickens which needs to be investigated.

**Facilitator:** Alright, let me ask you in this way, when you were designing these solutions did you overlook at those points? Because I believe that we were the ones that developed these solutions and did you discuss such issues concerning feedback for instance?

**Number 2:** Like my colleagues 4 and 3 have said already, all we need is feedback because after all the events what we were expecting from them was feedback but up to now we never got any feedback.

**Facilitator:** Alright, should we take a break or we should continue?

**Number 2:** Let’s continue

**Facilitator:** Alright, now I would like to hear about your interaction with the facilitators, how was your interaction with the facilitators of these meetings?

**Number 1:** The facilitators were good and they were very respectful

**Facilitator:** Alright, what about others? What do you think? Were they listening to you or the messages that they gave you was it enough?

**Number 4:** The facilitators were very open and they were speaking in a language that everyone could understand and we all felt free

**Facilitator:** Alright, what do others think?

**Number 3:** The facilitators were good, however we could have wanted if we had more experts but overall it was good and they were listening and they were responding to our questions but the experts were not given enough time so next time the experts should be given enough time.

**Facilitator:** Alright. Maybe which message about antimicrobial resistance was difficult to understand or which needed a further explanation?

**Number 3:** According to me don’t think there was any message that was difficult to understand but I just wish we could have more experts but I don’t think there was any message that was difficult to understand.

**Facilitator:** Alright. We are moving on. How about your interaction with different experts who were with you during the meetings, do you feel like they were listening to your ideas?

**Number 2:** According to me the interaction with the experts was good, they explained well about antimicrobial resistance, but the issue is just feedback like my friends have already said because it’s now about a year since our last meeting but there hasn’t been any feedback.

**Facilitator:** Okay

**Number 1:** I just want to add that I wish we could have been taking these kind of programs to a radio so that other people can also listen about it in order to prevent this problem

**Facilitator:** Alright, it seems like we have already started talking about where we are going next. Firstly, I want to ask about the process that you used to design the various solutions that you have mentioned, how did you see that process what process did you use?

**Number 2:** The process that we used was through groups, we were divided into groups and in each group we were coming up with solutions and at the end of it when we come back together to present the solutions some of the solutions were similar. So, we used group work.

**Facilitator:** Okay. How about others what do we think about this process of using groups?

**Number 4:** That process was very good because the group was made up with people from different places for example some were coming from [community name], some were coming from [community name], so when we form a group we were sharing experiences from our areas and we were generating solutions. And when the solution match with solutions of other groups we were considering that solution to be a good one.

**Facilitator:** Okay

**Number 3:** I want to agree with number 2 that the process of using groups was good because the groups were made of few people and we were able to listen to each other. The only challenge that I observed was that each group was given a different topic to discuss but I feel like it could have been good if all the groups were given all the topics to discuss. For example, I think we had four topics so what they could have done was that during the morning session each group could have been given two topics to discuss and then during the afternoon session each group could have been given the remaining topics. But apart from that the group work was good.

**Facilitator:** Alright. Are there any more opinions or we should continue?

(No answer)

**Facilitator:** Alright. I want us to talk about the co-creation event. What are our views in terms of the time, the venue that we used, how was it?

**Number 3:** The co-creation event was good because we received new visitors, some came from Lilongwe and some were joining us during the meetings so it was good. However, I was expecting that we would have senior visitors

**Facilitator:** More senior than the ones that came?

**Number 3:** Yes, because some of the solutions that we came up with we requested that they should be taken to the government, so they could have at least invited a deputy minister of agriculture or health.

**Facilitator:** Okay. So, you mentioned that you received other visitors, what kind of groups of people came?

**Number 3:** Some of them were from college of medicine, some were white people from Malawi Liverpool wellcome trust, some came from minister of health and some seniors were from ADD.

**Facilitator:** Alright, did we have our community leaders?

**Number 1:** Yes, we had a couple of chiefs

**Facilitator:** Alright, how do you feel about that arrangement that some visitors such as the chiefs should be joining you at the final event of the meetings, was it good that way or it should be changed?

**Number 4:** When we came here we didn’t come here to learn but to share ideas and come up with solutions so when the chiefs joined us our expectation was that they will take everything that we discussed to the communities but there has never been any feedback like my colleagues have already complained

**Facilitator:** Alright, but my question is those various leaders that joined you was it okay for them to be joining you at the end or they should have been joining you within the previous meetings? For example, you mentioned about the chiefs, is it good for them to be joining you at the final event or they should have joined earlier when you were discussing the solutions?

**Number 3:** They should have joined us from the start, I’m saying this because in our communities the fast way of spreading information is through chiefs, they spread messages during funerals and it gets to a lot of people, so those people are very influential and they should have joined us from the start.

**Facilitator:** Okay. How about others what are your opinions?

**Number 2:** I just want to add, apart from the chiefs I feel like we would have also included church leaders because those people also spread messages to the masses

**Facilitator:** Alright, Now I want to discuss about the various solutions that you developed, how feasible are the solutions that you designed in dealing with antimicrobial resistance?

**Number 3:** The solutions are feasible that they can solve the problem but it is difficult to comment on them now because it has been a while now and we have no idea if those solutions were tried out there but we needed feedback from you if the solutions were tried out. Because even if we take the messages to our colleague in our communities to tell them not to consume chickens which contain antibiotics they will still buy and consume them if they are cheap

**Facilitator:** What would make them neglect you and still consume the chickens that contain antibiotics what would be the reason for doing that because those are some of the challenges that I would like to hear?

**Number 3:** Due to financial problems and in short I should say poverty and I think we discussed about this during the meetings that some of the root causes is poverty and ignorance

**Facilitator:** Alright. And you also talked about need for resources to implement some of the solutions, in your perspective what challenges have you come across in implementing these solutions and how will that affect antimicrobial resistance problem?

**Number 1:** People are finding it difficult to accept it due to poverty that’s why I said there is need for civic education

**Facilitator:** Okay

**Number 3:** The other challenge is that some of the people they do bad activities that may cause AMR but those activities they are their source of income so even if you advise them to stop they will not stop because it’s their source of income

**Facilitator:** mmh

**Number 4:** The way things are currently operating in this country it’s even becoming difficult for the veterinary officer to conduct a test on the animals that are being slaughtered in the markets because they become overpowered by the people who neglect his decision to burn the animal if it has a problem

**Facilitator:** Alright

**Number 2:** I agree with what my colleagues have said, but in my community the big challenge is that there is lack of knowledge about this issue so it could have been good if we would spread the messages so that a lot of people should be aware of this problem.

**Facilitator:** Alright. Which groups of people have we shared the messages with and how was there reaction and what questions were they asking?

**Number 2:** As for me I have shared these messages with the youths who are vendors of drugs such as antibiotics but my only worry is that they just stopped selling in my community but they have moved to other communities.

**Number 3:** As for me I have spoken mostly to shop owners because I also happen to own a shop and the common complaint which they have been raising is that the antibiotics give them a lot of profit hence it’s difficult for them to stop selling them, so I think the main problem is failure to regulate these drugs by the regulatory authority

**Facilitator:** Okay. Do we have any other more ideas?

**Number 1:** As for me I have talked with the youths especially those that sell dressed chickens, when they go to buy the chickens they choose the chickens which are already dead instead of choosing live chickens and when I asked them why they are buying dead chickens they told me that they make more profit from the dead chickens because they buy them at a low price so I advised them to stop. And apart from that I have also spoken to my family members to seek medical assistance from the hospital and they should be taking the full dose

**Facilitator:** Alright, any other final words?

**Number 2:** My final words are that the government should put strict restrictions so that antibiotics should be protected

**Facilitator:** Alright. We have reached the end of our discussion, thank you very much for your time!
