# Supplementary material for: Co-creating community-driven solutions and policy priorities to address antimicrobial resistance through Responsive Dialogues: A qualitative evaluation from Malawi
Source: PLOS Glob Public Health. 2026 Apr 28;6(4):e0005697. doi: 10.1371/journal.pgph.0005697 (PMC13123971; doi:10.1371/journal.pgph.0005697)
Supplement: S2 Text — (DOCX) [file pgph.0005697.s002.docx]

**Facilitator:** Alright. We are starting up. Firstly, thank you for your acceptance to take part in this discussion. Before we start everything I would like to know each one of you, everyone can tell me what you do in your daily basis?

**Number 1:** On a daily basis I depend on business to earn a living, I’m a mobile money agent such as airtel money and Mpamba

**Facilitator:** Alright. How about others? By the way where do you come from?

**Number 1:** I come from [community name]

**Facilitator:** Alright

**Number 2:** On a daily basis I am an agro dealer, I come from [community name]

**Facilitator:** Okay. How about others?

**Number 4:** There is nothing much that I do on a daily basis but I’m a technician so I wait for people to call me if there is a piece work, I come from [community name]

**Facilitator:** What kind of a technician are you?

**Number 4:** I’m a general fitter

**Facilitator:** So you deal with electrical appliances?

**Number 4:** Partly but mostly I fix machines

**Facilitator:** Alright, anyone else?

**Number 3:** I’m also a technician and that’s what I depend on most to earn a living but my technical expertise is different from number 4 because I fix phones, radios and Tvs

**Facilitator:** Okay

**Number 3:** Apart from that I am also an assistant to a chief in [community name]

**Facilitator:** Meaning that you come from [community name]?

**Number 3:** Yes

**Facilitator:** Alright

**Number 5:** To earn a living on a daily basis I am a businessman, I sell dry fish and I’m also an assistant to a chief in [community name]

**Facilitator:** You come from [community name]?

**Number 5:** Yes

**Facilitator:** Alright. We are starting up. So, I would like to know what do you know about the issue of antimicrobial resistance?

**Number 3:** I have known about this issue 4 to 3 months ago during the research that I participated in when they were having discussions with men who had little knowledge or no knowledge at all concerning this issue.

**Facilitator:** Alright. What do other know about this issue of antimicrobial resistance?

**Number 4:** Indeed, at first I never know about antibiotics I was just consuming them, I wasn’t even able to compare an antibiotic with other drugs

**Facilitator:** Alright, what do others think or in terms of what causes the issue of antimicrobial resistance or what risks would it have on human and animal health?

**Number 1:** First of all, I should thank Malawi Liverpool Wellcome Trust for giving us knowledge on this issue

**Facilitator:** mmh

**Number 1:** Indeed, the risk is there, because in our communities it’s only few people that are aware of this problem but a lot of people still don’t know about it so there is a need to approach more people to address this issue more quickly

**Facilitator:** Alright

**Number 5:** I just want to add on what he said, we were indeed at risk because we were not aware of antimicrobial resistance as a result we were taking antibiotics without seeking prescription, so I should thank Malawi Liverpool wellcome Trust for sharing knowledge with us concerning this issue.

**Facilitator:** Okay. What do others think?

**Number 2:**  We indeed learnt from Malawi Liverpool Wellcome Trust that misusing antibiotics would have health risks on human health and we also learnt that antibiotics don’t cure flue

**Facilitator:** Alright. How can we prevent this issue of antimicrobial resistance?

**Number 3:** During the meetings we discussed some of the ways which would prevent this problem. Some of the ways which we discussed was raising awareness in the communities using megaphones, posters, radios and even leaflets. But there is nothing that is happening since we discussed and it has now been like 3months but nothing is happening.

**Facilitator:** Okay

**Number 2:** It is possible to prevent antimicrobial resistance. One of the ways of preventing this issue is by seeking prescription from the hospital whenever we feel sick rather than just buying from the markets on our own.

**Facilitator:** Alright. We are moving on. I believe you took part in the various conversation event that happened?

**Participants:** Yes

**Facilitator:** What is your experience for participating in those conversation events or what are your views for participating in those conversation events?

**Number 1:** I should thank Wellcome Trust for organizing those events because they have assisted us men to know more about this problem, previously as men we had a habit of not seeking prescription from the hospital because whenever we feel sick we were just buying drugs from the pharmacy but after participating in those conversation events personally I have changed from practicing that bad habit and where I’m coming from I have also shared messages on the same with several men, I have talked to maybe about 6 men during our bawo game session

**Facilitator:** mmh

**Number 5:** I also would like to add that these events were very fruitful not just to our families but with our colleagues that they shouldn’t just buy drugs without seeking a prescription from the hospital but I have been encouraging them that even if when they have fever or feeling any kind of pain they should visit the hospital to seek for a prescription. And it seems like people are able to understand the message clearly

**Facilitator:** mmh

**Number 5:** Despite that as men we had a problem of not visiting the hospital whenever we feel sick. But after sharing the messages most men now have started visiting the hospital to seek prescription. I should stop there.

**Facilitator:** mmh

**Number 4:** Malawi Liverpool Wellcome Trust has indeed assisted us a lot especially in our community. For instance, I have been announcing to many people that they should avoid using drugs carelessly and they shouldn’t be taking antibiotics anyhow. At first I should also say that I never had any idea what an antibiotic is but after the event that’s when I gained knowledge on what an antibiotic is.

**Number 3:** I also would like to add on what number 4 has said. First of all, let me thank Malawi Liverpool Wellcome Trust for organizing these events but my request is that they should be giving us feedback on whatever transpired after the discussions that we share.

**Facilitator:** Alright that’s understood. Now, I want to ask about the time that you spent at the events or the venue or the distance to the venue where the events took place or the venue itself, what are your views on those things?

**Number 4:** The issue of distance wasn’t a problem to me and I didn’t even have any problem with the venue. And the venue where the co-creation event took place was outstanding I never thought we would ever visit such a place in our lives but we went there thanks to Liverpool Wellcome Trust.

**Facilitator:** mmh

**Number 1:** The Malawi Liverpool Wellcome Trust Staff were also very good at assisting us. They were very respectful and very friendly

**Participants:** True!

**Number 1:** And the venue was very nice and they were also keeping time, they have never been late and sometimes we were the ones that would come late. The meeting materials were all enough and we thank them for that. Everything was good and very interesting.

**Facilitator:** Okay

**Number 4:** I agree with my colleague here everything was good and it was very beneficial, so because it was very beneficial I don’t feel like I wasted my time there, the time that I spent there was very necessarily to do that and both venues were very good

**Facilitator:** Okay. I believe number 1 already started answering on my next question. I would like to know how was your interaction with the facilitators of these events?

**Number 3:** Like number 1 already mentioned, the facilitators of these events were very helpful, they made everyone feel free and they were talking to us as their own friends. Wherever we don’t understand they were giving us an opportunity to ask questions. So to talk about the facilitators they were very competent and they did a great job and if you are to ask everyone that attended the meetings they will tell you the same.

**Facilitator:**  mmh

**Number 4:** I should also thank the facilitators they were indeed very good; they were not coming alone they were even bringing experts with them to join us and they made us to feel free and they were informative and we gained more knowledge from them.

**Facilitator:** Alright, so you have talked about experts, how was your interaction with these experts?

**Number 1:** Our interaction was very good and especially during the group work because each group had an expert so the experts were guiding us on our topics that we were discussing on the groups so the I would say the interaction was good and all he experts from Lilongwe and Blantyre they were very good and even some experts who own drugstores we with us so it was interesting.

**Facilitator:** mmh okay

**Number 3:** Like number has said the experts were really good and they were very open, they were respectful and they made us not to be scared of them or their knowledge, they were not teachers as such but they were also like learners. They were very competent and we appreciate having them on top of the Malawi Liverpool Wellcome Trust staff.

**Facilitator:** mmh

**Number 5:** I should also thank the experts for coming, they never wanted to look superior even though they had all the expertise but they were humble and made themselves look like they are learners.

**Facilitator:** Alright. Now I want to hear about the process that you used in designing the solutions, how was the process that you used?

**Number 3:** I will start by thanking the facilitators, they were really good at their job, the process which they used to develop the solutions was good, because every group was coming up with their own solutions and then at the end of it all we were coming together and share the solutions which was a good process. Because the group work helped us to come up with good solutions.

**Facilitator:** mmh

**Number 2:** The process was a good process, they held the conversation events with several groups of people such as assistants to the chiefs, business people and I also heard that they had the events with farmers, so I believe that using that approach it was good approach by Malawi Liverpool Wellcome Trust.

**Facilitator:** Okay

**Number 1:** To me I feel like the solutions did not benefit anything because when the solutions were presented from us we expected Malawi Liverpool Wellcome Trust to take those solutions to the government so that the solutions should be implemented but we never received any feedback from them up to now. For example, we had a solution whereby we requested the government to build health clinics closer to people but ever since the parliament was opened I never heard the members of parliaments discussing about these kind of issues.

**Facilitator:** mmh

**Number 1:** We also had another solution whereby we requested for the government to raise awareness on this issue through a mobile vehicle just the way they do with TB but ever since that we never got feedback on that. And apart from that we also agreed that we should have the chiefs during the meetings so that they should also take these messages to the people as one way of raising awareness but as I speak that is not happening in my community. So, mine is a request to Malawi Liverpool Wellcome Trust that they should put in efforts so that these solutions should be implemented. Thank you

**Facilitator:** mmh

**Number 2:** We discussed that some solutions are long-term solutions and some are short-term solutions. So, some of the solutions that he mentioned about such as building health facilities, those are long-term solutions. But my request is that at least if they should give us some leaflets which we can share with people.

**Facilitator:** Alright. So, it looks like we have ventured into the next topic but if I heard you all correctly it seems like the solutions that you developed are good but you lack some resources and you also feel like the solutions haven’t been implemented right?

**Participants:** Yes

**Facilitator:** So let us focus on that part now, do you think these solutions are feasible to deal with this problem of antimicrobial resistance?

**Number 3:** The solutions are more feasible but the only problem is hesitation because we talked about raising awareness through banners and mobile vehicles the same way they do with covid19 but nothing is happening. I feel like it’s only Malawi Liverpool Wellcome Trust which has a lot of interest in dealing with this problem unlike the government side. So it seems like there is a gap between people have information on this issue and people who need to have this information.

**Facilitator:** Okay

**Number 3:** Sure

**Facilitator:** What do some of you think on the feasibility of the solutions?

**Number 1:** The solutions that we developed are very feasible and some of them are already working. For example, I have already been encouraging my fellow men to visit the hospital when they feel sick but like I said we don’t have enough resources to approach to a wide range of people. So there is need to put in more interest especially for you Malawi Liverpool Wellcome Trust to create a better Malawi. Thank you

**Facilitator:** Alright. Are there any other more ideas?

**Number 1:** Yes, mine is more like a request to Malawi Liverpool Wellcome Trust, they should give us some resources that we should be using in spreading these messages for instance they would give us megaphones.

**Facilitator:** Alright. Now I would like us to talk about the co-creation event. What are your views on how that event was organized?

**Number 3:** According to me the co-creation event was an event that gave me a lot of interest because we were joined by new visitors who had some input in what we have been discussing and they responded to any question that we asked them and if the solution is good they were agreeing with us on spot. So, according to me that event was good.

**Facilitator:** Okay

**Number 4:** I would like to add on what number 3 has said. The final meeting which took place at the hotel was very good, we shared new ideas with people and we were challenging each other with the visitors.

**Facilitator:** Okay

**Number 2:** The co-creation event was very good starting from the venue, it was very nice and the materials that we used there were good, even the food was very good. We discussed the solutions with the visitors and we came up with solutions which may be used in the whole country. Thank you

**Facilitator:** Alright So, you mentioned that you were joined by other stakeholders during this event such as the chiefs and experts, what are your opinions on that arrangement that some other people should be joining you at the final event?

**Number 5:** To say the truth here when we were starting our events we started together with the chiefs and we involved them to the end but we were joined by other stakeholders such as members from the ministry of health and pharmacists who seemed to be bringing in new ideas.

**Facilitator:** Okay

**Number 1:** According to me it was very important to have new people at the co-creation event because we needed them to share their thoughts on what we discussed and agreed as the solutions, so their input was necessary and it was good to have them.

**Facilitator:** mmh

**Number 3:** I would like to answer on your question about involving the chiefs. I should thank Malawi Liverpool Wellcome Trust for recognizing that the chiefs are an important part of the community and if there is a fastest means to get to the people is through the chiefs. so it was important to involve the chiefs because that will help to spread the awareness messages faster to the people. I should stop there.

**Facilitator:** Alright. Now what I would like to hear from you is that what has changed in your daily activities or what are you planning to change in your daily activities after your participation in these events?

**Number 1:** According to me and my whole family our lives have changed because of my participation in the events, I’m saying it has changed because we are now fully aware that there is this particular problem and now we share this with other people for instance I always talk about it with my colleagues when I’m playing Bawo with them.

**Facilitator:** Okay

**Number 2:** These meetings were very beneficial to me and my family, previously we had a bad habit of not completing the full dose of drugs and we were not seeking prescription from the hospital but now we have stopped that habit when we feel sick we go to the hospital and seek prescription and we now complete the full dose of drugs

**Facilitator:** What challenges are we coming across in implementing this change or what you are planning to change in our communities or in our households?

**Number 3:** There is change in our households and in our communities but it is a challenge to completely implement the change due to lack of resources because we need to approach others to change and for us to approach many people we will need resources.

**Facilitator:** mmh

**Number 2:** I want to add on what he said, for us to fully implement the change we will need resources such as the hospitals to be closer to us and availability of drugs at the hospital at all times will also be an important factor to cement the change because people have a habit of not seeking prescription from the hospital because the health facilities are far from them and when they go there most of the times they don’t find medicine hence they opt just to buy from a private pharmacy.

**Facilitator:** Okay

**Number 1:** According to me I honestly feel like I don’t see the change because we don’t have much power in our hands to influence the change, I wish Malawi Liverpool Wellcome Trust could have given us the power to act as agents of spreading these AMR messages just like the way Airtel money agents and TNM mpamba agents operate, if they could make us agents people would be approaching us to seek AMR messages in our communities because if we are equipped enough there would be a big change in our communities.

**Number 5:** I should also agree on what number 3 said that we need resources to spread the messages such as megaphones. For us we have learnt about this issue but it is becoming difficult for people to believe us on when we are sharing the messages with them because we have nothing to show them as a evidence of what we are talking about so it is becoming difficult to influence the change due to lack of the resources.

**Facilitator:** Alright. We have approached the end of our discussion. Thank you very much for your time

**Participants:** Thank you!
