# Supplementary material for: Co-creating community-driven solutions and policy priorities to address antimicrobial resistance through Responsive Dialogues: A qualitative evaluation from Malawi
Source: PLOS Glob Public Health. 2026 Apr 28;6(4):e0005697. doi: 10.1371/journal.pgph.0005697 (PMC13123971; doi:10.1371/journal.pgph.0005697)
Supplement: S3 Text — (DOCX) [file pgph.0005697.s003.docx]

**Facilitator:** Alright we are starting up, firstly I would like to thank you for your acceptance to participate in this discussion, there is no right or wrong answers here, mostly I just want to hear your opinions and your experiences. So, before we go any further I would like to know each one of you, what do you do on your daily lives?

**Number 1:** I work at [health facility] as a pharmacy assistant. I started work in 2018.

**Facilitator:** Alright, anyone else?

**Number 2:** I work at [health facility] as a pharmacy technician and I have been working for 2years

**Facilitator:** Alright

**Number 4:** I work at [health facility] as an HSA and I’m also a focal person for TB, I started work in 2007 and my first work was at [health facility] before I was transferred to my current health center.

**Facilitator:** Alright

**Number 3:** I work at [health facility] as a pharmacist and I have worked for 7years.

**Facilitator:** Alright. We are going further with our discussion. Now, I would like to know that what do you know about the issue of Antimicrobial resistance? Anything that comes to your mind when you hear about antimicrobial resistance? Number three start for us?

**Number 3:** Antimicrobial Resistance is an issue whereby the microbes in our bodies develop resistance to antibiotics and even if you take the antibiotics when you are sick you cannot recover from them.

**Facilitator:** Alright. What do others know? Maybe how it starts or how it affects human health or anything that you know about it?

**Number 2:** Antibiotic Resistance is the situation whereby the antibiotics no longer work in our bodies because the bacteria has developed resistance to the drugs as a result it causes recovery failure, this affects our economy because we are forced to buy other expensive drugs to use.

**Facilitator:** Okay

**Number 4:** Antimicrobial resistance is caused by taking of antibiotics without following the prescription. Some people when have cough they don’t seek for a prescription rather they opt to take antibiotics such as amoxicillin or erythromycin without prescription

**Facilitator:** Okay

**Number 4:** Antimicrobial Resistance is also caused when we don’t complete the full dose of the antibiotics for example when we start to feel better after taking the antibiotics for few days some people stop taking the medicine before completing the dosage.

**Facilitator:** Alright. How can we prevent this or how can we resolve these issues?

**Number 1:** I think before we proceed to that, I would like to add that Antimicrobial resistance is also caused when we consume food that contain antibiotics such as vegetables

**Facilitator:** mmh

**Number 1:** Now let me get back to your question on how we can resolve this problem. This problem can be resolved by seeking prescription from the hospital and we should also make sure that the food we are consuming doesn’t contain antibiotics

**Facilitator:** Alright. How did you get the information about antimicrobial resistance?

**Number 4:** I got access to that information from our previous conversation events that I participated

**Facilitator:** Was that your first time to hear about it?

**Number 4:** It was my first time

**Facilitator:** Alright, others?

**Number 3:** I heard about it from college in Pharmacology course but I never knew that the problem is that huge until I participated in the meetings that were organized by Malawi Liverpool Wellcome Trust

**Facilitator:** Alright

**Number 2:** We indeed learned about this from school in Pharmacology course and my second time I heard about it from the radio, and during the responsive dialogues it was my third time to hear about it and that’s when I realized that it is a serious problem

**Facilitator:** Alright. We are moving forward. Now, I would like to hear about your experiences for participating in those conversation events?

**Number 1:** The meetings were good because there were a lot of new things which we learned as providers from them, so to me all the meetings were good and were beneficial

**Facilitator:** Okay, before we proceed, what are those new things which you learned about antibiotics?

**Number 1:** For example, as for me I never knew that you can also develop antimicrobial resistance through consuming food that contain antibiotics

**Facilitator:** Alright. How about others? What was our experience for participating in these various meetings?

**Number 2:** Previously at [health facility] we used to provide updates to the clinicians on the antibiotics that are available as a result the clinician was prescribing based on the antibiotics that are available rather than prescribing the recommended antibiotics, but when I participated in the meetings I learned that what we were doing was wrong and now we have stopped doing that bad habit

**Facilitator:** Okay

**Number 4:** Personally the meetings were helpful to me as an individual because I was also one of the people who used to take antibiotics without following the prescription but when I participated in the meetings I learned that taking antibiotics without prescription is bad and I also learned that we should be completing our dose

**Facilitator:** Alright. What was your experience in terms of your commitment to participate in those meetings or the time that you spent there or maybe the directions to get to the venue?

**Number 1:** To say the truth during my first day of the meeting I found the place not conducive for the meeting because it was at a church and it wasn’t clean enough so I had a negative attitude towards the venue but when we started the meetings I didn’t have any issue with the venue because everything was going good

**Facilitator:** mmh

**Number 1:** I should also thank our facilitators for keeping time and we were finishing exactly on the time that we had scheduled to finish. And even though the venue was far but we had all the resources to get there. So, to me everything was good.

**Facilitator:** Alright, how about others? And in terms of the venue if we are to look for another venue what things should we fix from your previous experience on the venue?

**Number 4:** To me I feel like it was located far, because as for me I was coming from [community name] and I had to board two min buses to get to the venue so most of the times I was arriving late

**Facilitator:** Okay

**Number 2:** I also want to agree that the venue was far and the environment was not conducive but it could be good if you would look for a better venue in town because all the learning materials were okay.

**Facilitator:** Alright, according to our experiences including the venue, is there anything that you would like to change for the meetings to be excellent?

**Number 1:** Yes, we need to change especially the environment of the venue, because for you to collect good data you need a good environment. People can opt not to attend the meetings due to the environment of the venue. And as it has also already been mentioned that some people were coming late because the venue was far so there is need to find a venue that is easily accessible by everyone that is participating in the meetings and if the resources are available it is better to choose another neutral location

**Facilitator:** Alright. We are moving forward. Now, I would like to know how was your interaction with the facilitators of these meetings?

**Number 4:** We had a very good interaction with our facilitators, they were guiding us when we don’t understand about something and they were very friendly.

**Facilitator:** Alright, how about others what do we think? Maybe in terms of listening to your ideas how was it? Or maybe in terms of the information which they gave you about antimicrobial resistance how was it?

**Number 3:** They were listening to our ideas very well and they were not rejecting our ideas because they were saying that they want to learn from us because we are the people who work on the ground and they were agreeing to our ideas. And they gave us information which was very competent to what we were learning and I was happy with such things.

**Facilitator:** Okay

**Number 2:** It was all good because they were not like teachers as such but we were all learning from each other, no one was superior to one another so it was interesting

**Facilitator:** Okay. Among all the messages which the facilitators gave you, which message was difficult to understand?

**Number 2:** They gave us messages about animals but for us we usually deal with people’s medicine rather than livestock so to us that was like a new area

**Facilitator:** Okay. We are moving on. Now I want to hear about your interaction with the experts who came to talk about antimicrobial resistance, how was your interaction with them?

**Number 1:** I think we already talked about it, the interaction was very good and to say the truth the experts which came according to me they were very competent and they were also able to listen and learn from us

**Facilitator:** mmh

**Number 1:** Likewise, with us we were also able to listen and learn from them so in short those experts were very competent

**Facilitator:** Alright. I want us to talk about the process that you used to develop them various solutions that you developed, what do you think about that process?

**Number 1:** It was a good process because the solutions which were developed were not just coming from one person but it was done as a group work each group was coming up with their solutions and then all the groups were presenting the solution to the whole group and if the groups present a similar solution we were taking that solution to be useful.

**Facilitator:** Alright, how about others? What did we like about the process?

**Number 2:** It was a good process because the ideas were coming from the whole group rather than one person

**Facilitator:** Alright

**Number 2:** Sure

**Facilitator:** Now I want us to talk about the co-creation event, was it similar to the previous events that you had?

**Number 1:** The co-creation event was very different from the previous events because firstly the environment was 100% excellent

**Facilitator:** Okay

**Number 1:** And secondly, we had other experts who were not with us during the previous events but during this final event we had extra experts and we were concluding everything that we we had been discussing. It was a good event.

**Facilitator:** Alright, how about others what do we think about this event?

**Number 3:** The co-creation event was good but it had a small problem.

**Facilitator:** Mmh

**Number 3:** The problem was that the groups had a lot of points to present but they were given a little bit of time to do the presentation because we were supposed to finish everything by 12pm, so the presenters were failing to present everything due to time

**Facilitator:** Alright. You mentioned that the venue was 100%, how do you compare it with the previous venue of the meetings?

**Number 1:** The first good thing about the venue was that it was a hotel and for us who are in a working class that’s a good place for us to have a meeting

**Facilitator:** mmh

**Number 1:** Secondly, in terms of transportation to get to the venue it was easy because the venue was in town and it was easy to travel to the venue

**Facilitator:** mmh

**Number 1:** And the problem was just time as she has already indicated, maybe we could have been there for three days or more but considering that it was a hotel maybe they were running away from expenses so that’s why we were rushed

**Facilitator:** mmh

**Number 2:** Like he said it was good but to me I was expecting that we would have the event in Salima or Mangochi

**(Laughter)**

**Facilitator:** What could be the benefit of having the event in another district?

**Number 2:** We are used to having such kind of meetings in those places

**Number 1:** We were coming from home so we needed a place where our minds were free from home and everything else

**Facilitator:** Alright. So, you mentioned that you were joined by other stakeholders at the co-creation event, what are your thoughts on that arrangement that other stakeholders should be joining you at the final event?

**Number 2:** It was good but the issue was just time, we didn’t had enough time to discuss

**Facilitator:** mmh

**Number 2:** But it was good because we had chiefs, HAC members and we also had stakeholders from the ministry

**Number 4:** I also just want to add on her point that those people that joined us were giving us insights on the points that we were raising but however sometimes they would talk about something that we had already discussed during the previous meetings

**Facilitator:** Alright. We are moving on. Now I want us to talk about the various solutions that you developed involving antibiotics, how feasible are these solutions to deal with antimicrobial resistance?

**Number 1:** I feel like even though we developed the solutions it is however difficult to implement those solutions easily in a Malawian setting for example one of the solutions that we came up with was that the government should deal with unauthorized drug sellers but that it becomes difficult for the government to implement that.

**Facilitator:** Okay. What challenges are you expecting that would make the solutions not to be possible?

**Number 4:** The first challenge is lack of antibiotics in government hospitals because that is the first reason why those unauthorized drug vendors choose to sell more antibiotics because they make more sells since people seek more antibiotics

**Facilitator:** Okay. What do others think? How feasible are these solutions that you came up with?

**Number 3:** Like she mentioned it sometimes the same people who own the drug shops are also employees of the public hospital so it becomes difficult to control such issues

**Facilitator:** Alright. What have we changed or what are we planning to change after participating in those events?

**Number 3:** I used to be one of the people who shared drugs such as amoxicillin or doxycycline with colleagues but after participating in the events I stopped that habit and I took leaflets from the event which I show everyone that ask me to share them drugs without prescription

**Facilitator:** Okay

**Number 4:** Before participating in these events I also used to take antibiotics without prescription but after participating in those events I stopped that habit I no longer share drugs without prescription with my children or neighbors and at church I’m also a chair person of health committee so sometimes I also teach people about what I learnt from the events so it’s helpful

**Facilitator:**  Alright

**Number 4:** Sure

**Facilitator:** Is there anyone that would like to add on what challenges are you coming across to implement these or what is making the solutions feasible?

**Number 1:** The big challenge is that people talk harshly to us when we refuse to share them drugs

**Facilitator:** Okay

**Number 3:** I also want to add that sometimes people find it difficult to understand from just explaining to them, so in my opinion I feel like we should have received big posters involving antimicrobial resistance so that we should post them in our pharmacies

**Facilitator:** Okay

**Number 2:** I also want to add that previously when a patient has fever they were asking me for antibiotics and I was giving them but after participating in the events I explain to them that not every fever requires an antibiotic and I ask them to seek prescription from the doctor

**Facilitator:** Alright

**Number 2:** Sure

**Facilitator:** It looks like we are at the end of our discussion, but I want to give this opportunity to anyone that has anything that they would like to add?

**Number 2:** If anything we will call

**(Laughter)**

**Facilitator:** Alright, thank you very much for your time!
