# Supplementary material for: Co-creating community-driven solutions and policy priorities to address antimicrobial resistance through Responsive Dialogues: A qualitative evaluation from Malawi
Source: PLOS Glob Public Health. 2026 Apr 28;6(4):e0005697. doi: 10.1371/journal.pgph.0005697 (PMC13123971; doi:10.1371/journal.pgph.0005697)
Supplement: S4 Text — (DOCX) [file pgph.0005697.s004.docx]

**Interviewer:** Alright, firstly thank you very much for taking part in our discussion today. And I will ask you to feel free. Mostly I would like to hear your thoughts.

**MP:** Alright

**Interviewer:** So first of I would like to know from the whole procedure that you went through, what do you know about antimicrobial resistance?

**MP:** After our discussion, the antibiotic drugs are also used by people

**Interviewer:** Okay

**MP:** For example, there is Penicillin, and some also mentioned about penicillin which they also give to animals. So, we are using the drugs on animals, yet the drugs are supposed to be used by humans

**Interviewer:** Okay

**MP:** So, due to lack of knowledge on how to use the drugs it’s what is causing the antimicrobial resistance

**Interviewer:** Okay

**MP:** So even when you use those drugs you don’t recover

**Interviewer:** Okay. Maybe what causes all this to reach the extent of antimicrobial resistance?

**MP:** It is caused due to use of the drugs without following it’s recommended instructions

**Interviewer:** Okay

**MP:** If we would be following the instructions of taking the drugs then the problem of antimicrobial resistance wouldn’t be there. But because we don’t follow the instructions that’s why it causes antimicrobial resistance. So, even if when you take the right drugs you still fail to recover

**Interviewer:** What challenges are caused as a result of that in a community like here or maybe to the animals?

**MP:** It makes the animals to be unhealthy and for instance you would have 50chickens, but 20 chickens would survive, and the rest would die due to things like those.

**Interviewer:** okay, what about the challenges which a community would face due to antimicrobial resistance?

**MP:** The challenge is that if the animal dies even if when you throw it away people still pick it up and use it as relish. You throw it away knowing that it contains drugs, but someone still picks it up and use as a result the person is affected with such a problem as well.

**Interviewer:** Okay

**MP:** mmh

**Interviewer:** Where did you learn about all this?

**MP:** I learnt that from Wellcome-Trust, I went in town to attend the discussion for several days.

**Interviewer:** Was it your first time to hear about it during that time or you already knew of the problem?

**MP:** According to me it was my first time to hear about antimicrobial resistance name

**Interviewer:** Okay, so you said you learnt that from Wellcome-Trust?

**MP:** Yes

**Interviewer:** In what way were you learning about these things?

**MP:** We learnt these through group discussions. I met different farmers there, and most of the farmers were poultry farmers who keep layers and broilers chickens

**Interviewer:** Alright

**MP:** But in terms of cattle farming that I do I was the only farmer that practices cattle farming, so that affected me because my colleagues were 5, 6 or 7 but most of the discussion was focused on chickens

**Interviewer:** Okay, as in the discussion was focused on chickens or the ones that were contributing the most were chicken farmers?

**MP:** Most of the contribution were coming from chicken farmers, such as how to take care of our chickens, how can we overcome this problem

**Interviewer:** Okay, so you have mentioned that the absence of people that practice the same kind of farming as you do it affected you, what do you mean by that?

**MP:** We didn’t talk much on the cattle farming side, that if cattle have a certain disease how would we treat it. That part wasn’t talked much, it seems like a lot of farmers were chicken farmers.

**Interviewer:** didn’t you try to give your opinions on issues like these?

**MP:** I tried but it didn’t bear any fruits however there was a Veterinary Officer who touch based on such issues

**Interviewer:** Alright. Now, I would like to hear your feedback on all the four stages that took place, how did you see them?

**MP:** All the stages and the opinions that people were suggesting were very good

**Interviewer:** Okay.

**MP:** What I saw to be the most important thing if you want to be successful in chicken farming you must buy recommended drugs and you should do that through the consultation of veterinary officers. If your chickens are sick, you need to consult a veterinary officer and he will guide you on what drugs are recommended for the chickens.

**Interviewer:** How about in terms of the preparedness of the whole events, what is your comment on how the events were organized for instance in terms of the time, maybe duration of the day, what is your comment?

**MP:** Alright, the way the organizers prepared for these meetings it was very well organized

**Interviewer:** Okay

**MP:** And time was allocated very well, even people who were coming from far places were arriving at a good time

**Interviewer:** Okay

**MP:** Sure

**Interviewer:** How long did you stay there during your first meeting?

**MP:** On the first day we started around 8 O’clock and we were knocking off at around 4:30pm.

**Interviewer:** Was this fine with you?

**MP:** everything was fine

**Interviewer:** How about in terms of the venue, how did you find the condition of the venue?

**MP:** The venue was very nice, and it was at the center whereby it was easily accessed by both farmers from Blantyre city and Blantyre rural. They choose a good place.

**Interviewer:** As in good in what way?

**MP:** In terms of travelling

**Interviewer:** As in you didn’t face challenges in travelling to the place?

**MP:** Yes

**Interviewer:** Alright.

**MP:** Sure

**Interviewer:** What new things did you learn from there?

**MP:** I learnt new things about chicken farming, I learnt that chicken farming is very profitable, and I thought of starting it but the only challenge that Identified is that it will require me to have electricity nearby because the chickens require light environment, and the other challenge is capital.

**Interviewer:** Did you learn about that during the first meeting, second meeting or third meeting?

**MP:** These discussions were divided into several sessions, such as we would have a first session whereby, we would discuss on how we would treat the chickens

**Interviewer:** Alright, so what you mean there is that most of the examples that were being given involved more about chickens?

**MP:** Yes

**Interviewer:** In terms of antimicrobial resistance that is?

**MP:** Exactly

**Interviewer:** Alright, so what did you like the most in terms of how the whole program was organized?

**MP:** What I liked the most was that there was an issue to do with big chicken farmers and others, the issue was that those big farmers their chickens are bigger comparing to the chickens from small holder farmers which determine the price of chickens to be different, so they were discussions to try and find a way if these two farmers would work together

**Interviewer:** Alright. How about what you think wasn’t organized well in this procedure?

**MP:** To me what I think was organized well was that I would have loved if they would have included at least 4 or 5 cattle farmers during the meetings

**Interviewer:** Alright, so in short what should be done better to solve those problems or what should be changed?

**MP:** I don’t think there is anything that has to be changed, the procedure that you are taking is very good

**Interviewer:** Alright. We are proceeding. Now I would like to hear from you, what is your comment on the facilitators of these discussions?

**MP:** The facilitators were very good people; they were very happy and open in how they speak, and they actually said that everyone should feel free to speak whatever we think is bad or good and the whole group indeed felt free to speak

**Interviewer:** Okay. Is there anything else?

**MP:** They are good people, and they are good at their job.

**Interviewer:** So, in terms of your opinions how did you see it, how were your opinions being received by them?

**MP:** Everyone’s point was being taken by them, there wasn’t any negative issue with that.

**Interviewer:** Alright, now in terms of the message that was being given at the event was enough?

**MP:** The messages which the boys were giving us was enough.

**Interviewer:** Why are you saying it was enough?

**MP:** Because it was clear, it was enough because they were speaking in a way that everyone should understand.

**Interviewer:** Okay 1`

**MP:** And I believe that everyone that was interested to listen has got something from it.

**Interviewer:** Was there any difficulty in understanding the messages that were being given there?

**MP:** Yes, on my side it was very difficult because at the time they contacted me I wasn’t feeling so well, so I was worried if I would make it to the meeting, but luckily by the day of the event I was feeling better.

**Interviewer:** Didn’t you think of replacing yourself with someone else?

**MP:** At first, I thought of telling my wife to attend instead of me, but I felt better then.

**Interviewer:** Didn’t they change the way they chatted with you in all those different stages?

**MP:** No, there wasn’t any change, because we were in groups of one, two and three, like that, then what you have discussed was being shared as a whole group and then we were discussing them together.

**Interviewer:** Alright, how about in terms of your chat with the experts, how was it?

**MP:** We were the experts at that time

**Interviewer:** But were there no other professionals or experts on the day?

**MP:** Yes, there was a certain group of people which came from Lilongwe, so I believe those are the ones you are talking about.

**Interviewer:** That group seemed to know more about the issue very well?

**MP:** Yes, those ones had more knowledge.

**Interviewer:** How was your chat with these people?

**MP:** Our chat with those people wasn’t much different from the chat that we always had with the facilitators

**Interviewer:** When you say it wasn’t different what do you mean?

**MP:** They were also speaking to us in the same way, and they also explained their knowledge according to the skills they possess.

**Interviewer:** What did you learn from this group?

**MP:** This group encouraged us to be united, and to also form groups. Because to get assistance in a group It’s easy

**Interviewer:** mmh

**MP:** Than to do it alone

**Interviewer:** Okay. How were your opinions being received with these experts?

**MP:** They received our ideas well, they listened to them.

**Interviewer:** How do you would change the chat with this group?

**MP:** I don’t see anything to be changed personally.

**Interviewer:** Alright we are proceeding.

**MP:**  mmh

**Interviewer:** Now I would like us to review the procedure that you took to come up with the solutions, how did you see that procedure?

**MP:**  This procedure was very good because when you are just an individual you just do what’s in your mind but as a group, we could share several ideas and come up with one solid idea that we all agree to.

**Interviewer:** What did you like the most about the way the solutions were designed?

**MP:** What I liked the most is that everything was being displayed at the front where everyone could see just like the way teachers do at school.

**Interviewer:** Okay, what would you like to change on the way the solutions were designed?

**MP:** To me I feel like everything was fine.

**Interviewer:** Alright, now I would like us to talk about the final meeting where I understand that there was presence of several people to co-create your ideas, how did you see that event?

**MP:** The final stage was very good, because we concluded everything on that day, and we even had a role play of what was discussed from the beginning up to the end

**Interviewer:** what satisfied you day?

**MP:** I was satisfied with the skills which the experts had shown on the day, and I was also satisfied with the role play that the farmers had organized.

**Interviewer:** Okay so you have mentioned about the skills which the experts had, what kind of skills were these?

**MP:** The skill to explain everything in a way that everyone would understand

**Interviewer:** What else happened apart from the role play?

**MP:** Apart from the role play, we discussed everything that we have been doing from the beginning as a way of reminding each other on what has been happening up to the end.

**Interviewer:** Alright, how about the time which you spent there, how do you see it?

**MP:** The time that I spent there was a good time, but only on the first day at the beginning we arrived at a good time but those who had organized the meeting were a bit late

**Interviewer:** So how did that affect your program?

**MP:** It affected it in a way that we left the place a bit late than the planned time

**Interviewer:** Alright, how about in terms of the venue of the place, how did you see it?

**MP:** It was a good place, it was quite without any noise

**Interviewer:** Wasn’t it difficult for you to find the place?

**MP:** No, the place was alongside of the road and the directions were clear

**Interviewer:** What can you change next time on how that final stage was organized?

**MP:** It’s been a while, but I don’t think there was anything that was supposed to change

**Interviewer:** Apart from the farmers what other group of people came to the event?

**MP:** The veterinary officers came, there was also another group of people, but I have forgotten where they were from

**Interviewer:** How about leaders such as the chiefs, didn’t they also come?

**MP:** The chiefs also came

**Interviewer:** Okay, so how did you see the procedure of having the other groups of people on the final event?

**MP:** It was good because they also shared some ideas on how farmers would take care of their animals and one of the points that was raised was that there should be unity between the chiefs and the community members.

**Interviewer:** Do you think it was fine for them to come on the final day or do you think they should have been coming on different days.

**MP:** I feel like a journey that you start together is much better, for instance if we had started together with the chiefs then that way it could have been better.

**Interviewer:** Alright, let’s proceed

**MP:** yes

**Interviewer:** Now how do you feel about the solutions that you designed?

**MP:** The solutions that we designed were very good and if we would work together with the chiefs and other groups of people and follow those solutions it would be very helpful

**Interviewer:** How feasible are these solutions?

**MP:** These problems would have been possible if we would involve other government senior officials at the ministry of agriculture to set policies to help in implementing the solutions, maybe that way it would help

**Interviewer:** Okay. What challenges do you expect to come across when implementing these solutions?

**MP:** We don’t see any challenges

**Interviewer:** You feel that they can be possible?

**MP:** Yes, it’s all about coordination between the chiefs, community members and the government

**Interviewer:** Apart from coordination, is there anything else that would make these solutions more feasible?

**MP:** No, that’s the only one

**Interviewer:** Alright, but do you think they would bear fruits? or they still need to be worked on?

**MP:** They would bear fruits if we are all united but if we are not united then it wouldn’t work

**Interviewer:** Alright, let’s get back to the final section, Now I would like to understand from your experience and all the knowledge that you gained from the discussions, what behaviors have you changed or since you finished the discussions what impact have you made on antimicrobial resistance?

**MP:** What I have done?

**Interviewer:** Yes, or what you planning to do differently?

**MP:** What I have done is that I tried to keep chickens without giving them the medications and what I have realized is that a farmer would have a loss because most of the chickens would die, because for my chickens I was just keeping them in a free-range system as local chickens

**Interviewer:** Okay so this system of not giving chickens drugs was it one way of which you discussed?

**MP:** I wanted to check if it’s possible to keep them in that way

**Interviewer:** So, what challenges would you incur if you are to do that?

**MP:** I noticed that a farmer wouldn’t gain profit and the farming wouldn’t grow forward

**Interviewer:** Is there anything else that you have done, or you are planning to do differently from what you learnt from the events?

**MP:** The other thing that I have done differently is that at first, I used to buy inject my cattle with drugs on my own to get lead of mites but after I attended the meetings, I have now joined a group and we have built a dip tank where we clean our cattle

**Interviewer:** That idea came due to the conversation events?

**MP:** Yes, when I met with my fellow farmers we decided to try if this can be possible and when we tried it was made possible

**Interviewer:** Alright, you have just mentioned a bit about your fellow farmers whom you talked with about these discussions, which people did you talk to about the various messages that you received?

**MP:** I talked to my fellow cattle farmers, that what if we build a dip tank that we can all use to get lead of the mites on our cattle instead of everyone buying the drugs individually to treat the cattle, but as a group it would be cheap

**Interviewer:** Alright, is there anyone else that you talked with about antimicrobial resistance?

**MP:** No, just my fellow farmers

**Interviewer:** When did you had that talk with those colleagues of yours?

**MP:** It was in October

**Interviewer:** How long did it take from the final conversation event?

**MP:** Maybe two weeks

**Interviewer:** Where did you meet?

**MP:** At the dip tank

**Interviewer:** So, what was their reaction?

**MP:** They only wished if the organizers of the event would come and see what we are doing here

**Interviewer:** Before I close our discussion is there anything else that you think you have forgotten to say before we finish?

**MP:** The other thing that I can say is that there are other places where they sale drugs and what happens is that those people took authorization to sale drugs yet the person who they employ to work in the drugstore doesn’t know anything about drugs such as how they work and the type of drugs they are and what they do.

**Interviewer:** Alright, thank you very much for your time

**MP:** Thank you
