# Supplementary material for: Co-creating community-driven solutions and policy priorities to address antimicrobial resistance through Responsive Dialogues: A qualitative evaluation from Malawi
Source: PLOS Glob Public Health. 2026 Apr 28;6(4):e0005697. doi: 10.1371/journal.pgph.0005697 (PMC13123971; doi:10.1371/journal.pgph.0005697)
Supplement: S5 Text — (DOCX) [file pgph.0005697.s005.docx]

**Interviewer:** Alright we are starting, firstly I would like to thank you for giving me your time to have this discussion with me, mostly I want to hear your thoughts so I should encourage you that there are no wrong answers here, I just want to hear your thoughts.

**FP:** Alright

**Interviewer:** Firstly, I would like to understand that what is your occupation?

**FP:** I live in [community name] in Blantyre

**Interviewer:** Mmh

**FP:** My daily Job is to tame animals such as goats and chickens and I also go to the farm to take care of my crops. That’s how I spend my day.

**Interviewer:** You only tame goats and chickens and no other animals?

**FP:** No just local goats and chickens

**Interviewer:** Alright, we are starting up, firstly I would like to understand that what do you know about antimicrobial resistance?

**FP:** Antibiotic resistance occurs when a person has received antibiotics but hasn’t completed taking the dose

**Interviewer:** Okay

**FP:** For instance let’s assume that at the hospital they have given you drugs such as amoxicillin, maybe 30tablets and they tell you to finish them all, but when you start to feel better after taking 5 to 6 tablets you stop taking the antibiotics because you feel like you have recovered and after a month you will notice that you have started the same sickness and then you go back to continue taking the same drug which is wrong.

**Interviewer:** mmh

**FP:** But if the doctor has given us 30tablets we are supposed to finish all the tablets and in that way our body wouldn’t be affected by antimicrobial resistance. And that’s it on the human side

**Interviewer:** mmh

**FP:** And now when it comes to the animals, we were giving drugs to animals for example the goats, I was giving them doxycycline when they have diarrhea

**Interviewer:** mmh

**FP:** So, when you give the goat doxycycline the goat would feel better but after few days it would get sick again and at the end of the day it would be dead because you didn’t give it the right medications, that job has got its own specialists

**Interviewer:** Including you

**FP:** Including me, especially the veterinary officers

**Interviewer:** Alright, so you sort of already started talking about wat it should be done to prevent this problem, can you elaborate?

**FP:** To prevent this problem we should make sure that we finish all the doses that we receive at the hospital

**Interviewer:** What challenges would that cause?

**FP:**  If an individual doesn’t finish the dosage, he or she may get sick frequently as a result the person may fail to take care of his or her family, if the person is a student, then he or she might not do good in school because of frequent sickness

**Interviewer:** Alright, where did you learn about that?

**FP:**  I have learnt about that from Malawi Liverpool Wellcome-Trust during the group discussion

**Interviewer:** Was it your first time to hear about it or you already knew about it previously?

**FP:** I used to hear about it previously, but I wasn’t taking it seriously, I was hesitating I used to think it’s just a lie but when this organization came in that’s when I realized that it is a serious issue.

**Interviewer:** So, you mentioned that you were having discussions, how did you see that procedure?

**FP:** The procedure was very good because everyone including me learnt a lot from the discussions

**Interviewer:** Can you give me an example of some of the things that you learnt?

**FP:** For example, our friends who keep chickens sometimes they sale the chickens which have died on their own at a lower price like K2000 and people buy and eat that which would cause sickness and not just chickens but even goats

**Interviewer:** What else have you learnt about antimicrobial resistance?

**FP:** That’s all I remember

**Interviewer:** Alright, how about in terms of how the program was organized maybe in terms of time, what do you think about it?

**FP:** Their program was well organized, and they were keeping time and they were starting at the exact time as planned. They were not taking our spare time.

**Interviewer:** It wasn’t disturbing you from your daily activities?

**FP:** No

**Interviewer:** Alright. How about in terms of the venue where you were meeting at?

**FP:** There was nothing wrong with the venue

**Interviewer:** You were not finding it difficult to travel there in terms of the directions?

**FP:** No

**Interviewer:** In all the meetings it has been well like that?

**FP:** Yes, in all the meetings it has been like that

**Interviewer:** Okay

**FP:** sure

**Interviewer:** What did you like the most about how the whole program was designed?

**FP:** What I liked the most is that I have learnt a lot especially in chicken farming, for instance some farmers feed their animals drugs in order to fatten them and that is a bad practice from what I learnt

**Interviewer:** Okay, in terms of a human perspective or animals?

**FP:** In terms of human perspective, because people are the ones that will eat those chickens and as a result, they consume it together with the antibiotic which are in the chickens and as a result if the person gets sick and seeks medical treatment, he or she may fail to recover because of the drugs that he or she consumed with the chickens

**Interviewer:** What didn’t you like about the whole program which you wish should have been done better?

**FP:** Everything was fine, I don’t think there is anything that should have been changed

**Interviewer:** Alright, now I want us to talk about your interaction with the facilitators, how were you interacting with them?

**FP:** They were respectful boys, they were speaking to us nicely, they were not harsh on us, and we interacted very well as friends

**Interviewer:** Okay, how about in terms of your ideas, were they listening to your ideas?

**FP:** They were listening to us properly

**Interviewer:** How about in terms of the messages, were they giving you enough messages or you feel like they were not giving you enough messages?

**FP:** What kind of messages?

**Interviewer:** Messages about the whole program, maybe about antimicrobial resistance, do you think it was enough?

**FP:** Actually, it was very enough

**Interviewer:** Maybe how hard was it to understand the messages if there was anything that was hard?

**FP:** It wasn’t any hard because we were having group discussions and after that we were discussing all the ideas as a whole group then we would break back to the groups again and continue with the discussions.

**Interviewer:** Alright, from your conversations do you see anything that you feel like it can be changed?

**FP:** No, I don’t see anything that can be changed

**Interviewer:** It was all good?

**FP:** Yes, it was good

**Interviewer:** Alright, how about your interaction with the experts, how was it?

**FP:** We were all discussing as a group there was nobody that was taking himself as an expert

**Interviewer:** Alright, so you are saying that you were all discussing as a group, what new things did you learn from these people which you were not aware about?

**FP:** mmh

**Interviewer:** Or maybe let me start by asking that apart from you the farmers and the facilitators, was there any other people who came who seem to have more knowledge about the antimicrobial resistance?

**FP:** During the final day of the discussions, we had visitors who came from Lilongwe, who arrived a bit late maybe because of time, so we had a discussion with them for a short time but here was that group of people who came from Lilongwe.

**Interviewer:** Alright, they only came on the last day?

**FP:** Yes, on the final day

**Interviewer:** During the events you only had the facilitators?

**FP:** Yes

**Interviewer:** Alright, how did you interact with the people who visited from Lilongwe?

**FP:** We interacted with them very well and they were excited to see what we have learnt

**Interviewer:** Is there anything that they shared with you?

**FP:** They encouraged us that what we have learned we should share it with other people in our communities especially about antimicrobial resistance. Because to say the truth this problem it truly exists in the communities, if a child has minor cough, you will find that a parent rushes to buy drugs such as Bactrim and we even find it in groceries in the community

**Interviewer:** Do you feel like your ideas were being accommodated by these people?

**FP:** Yes, they were listening to our ideas

**Interviewer:** Okay, what can you change on your interaction with these experts?

**FP:** I don’t see anything that should be changed.

**Interviewer:** Now I want us to talk about that procedure that you took in designing the solutions, how did you see that procedure?

**FP:** It was fine

**Interviewer:** What did you like about the procedure of designing the solutions about wat you discussed in the groups?

**FP:** During the discussions I liked it that I learnt about the dangers of misusing antibiotics and the importance of finishing the full dose of antibiotics

**Interviewer:** Is there anything that you didn’t like about the procedure of designing solutions?

**FP:** No, everything was just fine

**Interviewer:** Maybe in terms of listening to each other?

**FP:** Everyone was listening to one other clearly. It was all good.

**Interviewer:** Alright, now I would like us to talk about the final stage were you met with different groups of people to discuss, how was that stage?

**FP:** It was also good

**Interviewer:** Okay, where did you do this event at?

**FP:** We did it at [community name] at a certain church, but I have forgotten its name

**Interviewer:** What happened there?

**FP:** We were encouraging each other as farmers, there were different groups of farmers, some keep layers chickens, others keep broilers chickens, pig farmers, goat farmers even cattle farmers, we were all there but we all came from various areas.

**Interviewer:** So, you also mentioned that you had visitors from Lilongwe?

**FP:** Yes, they came on the final day of our meetings

**Interviewer:** So, you only had them on the final day?

**FP:** Yes

**Interviewer:** You didn’t have other people like the chiefs?

**FP:** Oh! The chiefs came on the final day as well, we had the chief from [community name], we had the chief from [community name], we also had a local chief, we had a veterinary officer

**Interviewer:** Alright, so how did you find this procedure, or maybe in terms of the time, how did you see it? Or maybe duration?

**FP:** It was all good and nothing was wrong

**Interviewer:** Okay, were they listening to your ideas?

**FP:** To say the truth they were listening to us; it was all progressing well.

**Interviewer:** Alright. So, you mentioned that there is another group of people which came on the final day, what is you comment about that?

**FP:** It was encouraging to us because at first, we were only the farmers but when we received the other group of people such as the local chiefs it was very encouraging because the chiefs are the ones which people listen to in the community

**Interviewer:** Alright, but do you think it was alright that way, or you wish they could be coming on different days?

**FP:** It was fine the way it was the chiefs came on the last day and that was good because during the first meetings it was our time to cook the solutions that we had discussed

**Interviewer:** What do you think it should be done differently?

**FP:** Nothing

**Interviewer:** Are there any challenges that you encountered?

**FP:** It was all good, they were even giving us food

**Interviewer:** mmh

**FP:** However, on one of the meetings we complained that the food wasn’t well cooked

**Interviewer:** What happened?

**FP:** It seemed like the person that was supplying them with the food was running late so he just prepared the food in a rush

**Interviewer:** Okay

**FP:** Yes, but it was just for that day only

**Interviewer:** Alright, we are proceeding. So, I want to discuss about the solutions which you came up with in addressing the several issues that you had discussed, what do you think about those solutions?

**FP:** They were good solutions for instance we encourage ourselves to share the message about antimicrobial resistance with our colleagues especially our fellow women maybe at the hospital, we also talked about use of veterinary officers to assess the animals before taking them to the market for sale.

**Interviewer:** Alright, how feasible do you see the solutions that you came up with?

**FP:** They are possible to implement, it’s easy to open up people’s eyes because some people do bad things due to lack of knowledge and some people just do the bad practices due to poverty.

**Interviewer:** Maybe what challenges should we expect in implementing the solutions that you came up with?

**FP:** The only challenge is that some people may not listen to us even if when we tell them about it

**Interviewer:** why is that?

**FP:** Because like I said some people do bad practices due to poverty

**Interviewer:** Alright, any other challenges?

**FP:** No

**Interviewer:** Alright. Moving forward is there anything that you are doing differently, or you have planned to do differently as a way of addressing the issue of antimicrobial resistance?

**FP:** The difference that I have made is that I have the knowledge on the dangers of antimicrobial resistance and even here at my house when a child gets sick, I’m making sure that they complete the full dose of the medications, even relatives and colleagues I’m also encouraging them to finish their doses

**Interviewer:** mmh

**FP:** Even when I here that someone is selling meat somewhere I go there to ask if the meat is eligible to be sold

**Interviewer:** Okay, so you have mentioned that you are encouraging relatives and friends to complete their doses and you have also mentioned that you want people to be more aware of the dangers of antimicrobial resistance, how feasible do you find these solutions?

**FP:** They are very possible

**Interviewer:** You don’t think you would come across several challenges maybe?

**FP:** The challenges will be there but like I said some people won’t listen so maybe in a group of 10people only 5people will listen to you but that won’t matter a lot because you have at least saved 5people from the problem

**Interviewer:** Who have you talked to about the messages which you have acquired through this procedure?

**FP:** I have talked with several friends of mine, and I have also talked with my fellow women at the church’s women’s group, so I have talked with my friends about it.

**Interviewer:** How did these women react to it?

**FP:** They reacted well to it, they all acknowledged that what I was saying is true

**Interviewer:** Do you think they understood very well to the message?

**FP:** Yes

**Interviewer:** What kind of question were they asking maybe?

**FP:** Mostly it was just a comment like I said that some complain that they do such kind of things due to lack of money

**Interviewer:** Alright we have reached the final part of our discussion; I would like to give you an opportunity to add any comment that you maybe you might have forgotten something that you may like to add?

**FP:** My only comment is just to appreciate what Malawi Liverpool welcome trust in giving me the opportunity to participate and I would like to encourage them to continue what they are doing, and the chiefs should also be informed of these issues

**Interviewer:** Can’t you inform the chiefs?

**FP:** I can but like I in the communities’ people would listen to the chiefs more than just me alone

**Interviewer:** Alright, thank you very much for your time

**FP:** Thank you
