# Supplementary material for: Co-creating community-driven solutions and policy priorities to address antimicrobial resistance through Responsive Dialogues: A qualitative evaluation from Malawi
Source: PLOS Glob Public Health. 2026 Apr 28;6(4):e0005697. doi: 10.1371/journal.pgph.0005697 (PMC13123971; doi:10.1371/journal.pgph.0005697)
Supplement: S6 Text — (DOCX) [file pgph.0005697.s006.docx]

**Interviewer:** So, we are starting, firstly, I should thank you for accepting to have this discussion with me, mostly I would like to hear more about your ideas about antimicrobial resistance and I also want to hear about your experiences as you were progressing through the whole procedure, so feel free, there is no right or wrong ideas, so firstly I want to know what is your daily occupation?

**MP:** I’m a person who has worked before, the job that I was doing was refrigeration and air-conditioning but currently I work independently when I’m booked by companies, organizations and even by people.

**Interviewer:** Okay

**MP:** I am also an electrician and apart from that I practice poultry farming and I also have pigs

**Interviewer:** Alright

**MP:** These are the things that I do on daily basis

**Interviewer:** What do you know about antimicrobial resistance or how do you understand this issue?

**MP:** My understanding is that they are micro-organisms which fight against antibiotic drugs. My understanding is that us farmers we have been using those drugs inappropriately, maybe by overdosing our animals or by storing the drugs over its expiry date we were doing that because there wasn’t any guidance, so at the end of it all just like the way it happens with humans, when we give our animals drugs, they were not recovering from it. So, we were making a big loss because our animals were dying.

**Interviewer:** Alright, what challenges would emerge from it?

**MP:** Yes, this would bring a very big problem to people because I have already said that if we eat a chicken eat a chicken which is resistant then that would also affect us in such a way that if we get sick and receive medication we might also not recover from the drugs. So, there we be like a circle whereby those drugs from a chicken will enter into humans. And as a farmer if I sale affected chickens then I will also put many people that will consume those chickens in danger.

**Interviewer:** Alright what community challenges would it cause?

**MP:** In a community like here if they eat chickens which are resistant, they would also be affected but it is the responsibility of me as a farmer to burn all those affected chickens with paraffin or petrol than just throwing them away anyhow

**Interviewer:** Okay, so you have just started, what would be the other way of preventing these issues?

**MP:** The first preventive measure is to follow all the instructions that have been given to you by a veterinary officer.

**Interviewer:** mmh

**MP:** For example, if I’m a pig farmer and I have observed that one of my pigs is sick, the first right thing to do is to consult a veterinary officer to recommend the right drugs for the animal. The problem that the farmers had before the meetings with the Malawi Liverpool Wellcome Trust was that when their animals are sick, they were making their own judgement and buy whatever drugs they decide without consulting a veterinary officer or they were just asking for drugs from their neighbors and give them to their chickens or pigs as a result they were giving the animals wrong drugs

**Interviewer:** Alright, where did you learn about all this?

**MP:** I learnt this from an organization of Malawi Liverpool Wellcome Trust last year.

**Interviewer:** Okay

**MP:** I happen to be a chairman of a Poultry Farmers’ Cooperative

**Interviewer:** Okay

**MP:** So, they found us at agricultural fair at Trade fair where we were displaying our produce. So those boys were interested in what we were doing, and they told us that they would like us to join them and take part in their meetings, so we had those meeting at [community name] and they also listened to our ideas on antibiotic resistance among our animals

**Interviewer:** Alright, what was your experience from the whole process?

**MP:** My experience from the meetings and the group discussions conducted by the Malawi Liverpool Wellcome Trust is that it doesn’t matter how big the problem is but whenever our animals are sick, we should be seeking help from a veterinary officer

**Interviewer:**  How about the venue where you were meeting at or time that you spent there, what would you comment on that?

**MP:** According to me maybe I should appreciate because I didn’t see any problem with the venue because everything that we were expecting to have for the discussions it was there

**Interviewer:** How about in terms of the time that you spent there?

**MP:** There wasn’t anything wrong with the time that we spent there, to me the time was just fine

**Interviewer:** Maybe you just started talking about it, what new thing have you learnt about antimicrobial resistance?

**MP:** The other thing that I have learnt is that us the farmers should learn to take care of our animals without always depending on drugs. Some of the way that we discussed include general hygiene such as cleaning the animal stalls, giving our animals clean water, this would prevent our animals from getting sick

**Interviewer:** Alright, in terms of how the whole process was organized what did you like or what didn’t you like?

**MP:** To say the truth everything that happened there was good. In terms of the time sometimes we were coming early, and they would be a bit late and sometimes we would come late but they would come early but this to me wasn’t a problem because on the road we meet different delays

**Interviewer:** mmh

**MP:** But everything that happened throughout the process to me was well because there was keeping time, before we start the meeting, we were choosing a timekeeper and they were also telling us that we will have a tea break then we were having lunch there also because were knocking off in the afternoon. So, to me the whole process was organized professionally

**Interviewer:** Alright we are proceeding; I want us to discuss about the interactions that were taking place during the meeting. Firstly, I want to know how was the interaction between you and the facilitators?

**MP:** The process which they followed was good, the first thing they wanted to learn from us the farmers. So, we were more like teaching each other because they were learning and we were also learning some things from them, for example some farmers give ARV to pigs which is a bad practice.

**Interviewer:** Okay. Would you say your ideas were being accommodated?

**MP:** Our ideas were being accommodated, I will give a good example, there was like some board where they were writing down every point that we have contributed, and we were having a special time to prioritize what we have been discussing

**Interviewer:** Alright how about in terms of the messages that you received, how difficult was it to listen to the message?

**MP:** According to me every message that they gave me was very clear and at the beginning of the first meeting we all agreed to use Chichewa in our discussion so everything that we had discussed was in Chichewa, even the papers that were handled to us were written in Chichewa, so everyone was free to speak and hey were giving us a chance to ask questions wherever we don’t understand.

**Interviewer:** Is there anything that you would like to be changed on how you interacted with them?

**MP:** I don’t think there is any change that need to happen because the main focus of our discussion was to find the challenges that farmers are facing with the antimicrobial resistance, I would just like to request that in the future there should be an opportunity to have these discussions with other farmers, of course here we have a farmers club where we discuss some of these issues because I have sat down with my fellow farmers in our farmers club and they were interested to hear more and they even asked me a lot of questions

**Interviewer:** Maybe we should discuss that a bit, when did you have that discussion with your farmers club?

**MP:** When we had finished our meeting I met with my members of our Poultry Farmers Cooperative, and we discussed issues of how we are supposed to administer drugs to our animals in a right amount to avoid antimicrobial resistance and we also discussed about the importance of consulting a veterinary officer before administering drugs to the animals.

**Interviewer:** So, what was their reaction?

**MP:** They reacted well to it because even some of them were telling me that they have gone through such situations whereby they were sharing drugs with other farmers, but the chickens didn’t recover and all the chickens died, so they listened to what I told them, and they were willing to follow that

**Interviewer:** Alright, so we are proceeding. I want us to discuss about your interaction with the experts, how was your interaction with them?

**MP:** The main purpose of these people mostly was to guide us on whatever experiences that we explained to them, so they were guiding us on the recommended approaches that we need to follow for the good health of our animals for example they told us that if we have administered an injection to a dairy cow and the instructions are telling us that the animal shouldn’t be eaten for at least 10days then even if the cow has milk then all the milk for that period is supposed to be thrown away because that milk still contains the drugs.

**Interviewer:** Were they accommodating your ideas or your words?

**MP:** All our words and ideas were being taken by them very well, I’m saying this because every farmer was coming from different parts of Blantyre some came from [community name], [community name] and [community name], so some of the farming practices that were being followed by other farmers wasn’t being done by other farmers, for instance some farmers were saying instead of using modern drugs they were just using traditional herbs such as nimu and it works.

**Interviewer:** mmh

**MP:** But I’m not saying it’s recommended to do that, because I remember we received one of the visitors who came from college of medicine, and I asked him a question to say out there we have a lot of people who are selling traditional herbs and I asked if that practice was authorized, and he said they are still conducting research on that.

**Interviewer:** Is there anything that you would like to change on your interaction with these experts?

**MP:** I don’t think there is anything that needs to be changed because most of the ideas were coming from us

**Interviewer:** Alright, we are moving on. Now I would like us to discuss about the process of designing solutions, how did you see that process of designing solutions for various problems that you discussed?

**MP:** The whole process to me it was good, we were having flip charts

**Interviewer:** Okay

**MP:** And we were being divided into groups and each group was discussing the problems and then we were presenting what we have discussed in the groups to the whole group as a whole and at the end of it all we were identifying solutions that were agreed by the whole group and the experts plus us we were prioritize the solutions and solution that were similar were being merged.

**Interviewer:** What didn’t you like about the process of designing solutions?

**MP:** To say the truth the process which they used was very good because the facilitators were very open, and they didn’t just give us the information and leave us, but they were guiding us on whatever we are trying to mean when explaining an idea. So, there wasn’t anything that I didn’t like.

**Interviewer:** Alright. Now I want us to discuss about the co-creation event, which was the final stage of your meetings, how did you see that stage?

**MP:** What happened was that we received some visitors from the laboratory who came from Lilongwe, and we also received some visitors from the ministry of agriculture and when we met them, they also showed interest in what we were discussing, and they encouraged us to consult a veterinary officer when we want to administer drugs to animals. They guided us in several things.

**Interviewer:** Alright, how about in terms of the time that you had spent there, how did you see it?

**MP:** There wasn’t any problem with the time it all went on very well because every session was given its own time including the breaks. So, to me it was interesting

**Interviewer:** How about in terms of giving your ideas, do you feel that you presented your ideas freely?

**MP:** Yes, they were writing down everything that everyone was suggesting and at the end then we were choosing what we thought to be feasible by us the farmers on our own. So, to me I was happy because they were taking everyone’s idea.

**Interviewer:** Alright, so you have mentioned that some other visitors came from Lilongwe to join you, what do you think about that process that some other people should be joining you at the end when you have already passed through other stages?

**MP:** To me I don’t see any problem because they were experts so to me it was good that they came at the end, and they were given a chance to speak to us and they sort of added other ideas on top of what we have already been discussing with the facilitators from Malawi Liverpool Wellcome Trust.

**Interviewer:** Alright, we are moving on. I want us to discuss about the solutions that you came up with

**MP:** Alright

**Interviewer:** What do you think about these solutions?

**MP:** The solutions that we came up with were very good solutions to me

**Interviewer:** How feasible are these solutions according to you?

**MP:** Maybe the only challenge could be lack of money, but they are good solutions

**Interviewer:** Alright, you have mentioned about money, what other challenges could you come across in implementing these solutions?

**MP:** The biggest challenge is lack of money

**Interviewer:** Alright, going forward what have you done differently or what are you planning to do differently from what you have learnt through the whole process?

**MP:** Ever since we finished the meetings with the members from the Malawi Liverpool Wellcome Trust, I gave myself time to make sure that before I do anything I should be speaking to a veterinary officer and also, I should give my chickens clean water and clean food. And this has improved the health of my animals.

**Interviewer:** Okay, how about in terms of the issue of fighting against antimicrobial resistance, what have you done differently?

**MP:** On this issue I have stopped buying drugs anyhow, before I buy drugs, I seek for consultation with a veterinary officer to assist me with the problem, this has helped me a lot because like I said earlier at first, I just used to make judgement out of assumptions on what drugs would work.

**Interviewer:** What challenges are you coming across in trying to do that?

**MP:** All the time when you are following some instructions there is need for money to fulfil those needs, for instance for me to give my animals clean water I will need to have a proper piped water and I will need to have electricity to provide light for my chickens which requires money.

**Interviewer:** Alright before we close our discussion, I would like to give you an opportunity to say anything that you feel like is left out?

**MP:** My last words that I would say is that firstly, I should thank you for coming.

**Interviewer:** Okay

**MP:** This process that you are taking if we had started doing it 10years ago farming could have gone far in the country. Even some of the issues that we discussed my colleagues come to me and ask for ideas on how we would go on with some of the solutions and that is giving me encouragement.

**Interviewer:** Alright, thank you very much for your time, this is the end of our discussion.

**MP:** Thank you.
