# Supplementary material for: Co-creating community-driven solutions and policy priorities to address antimicrobial resistance through Responsive Dialogues: A qualitative evaluation from Malawi
Source: PLOS Glob Public Health. 2026 Apr 28;6(4):e0005697. doi: 10.1371/journal.pgph.0005697 (PMC13123971; doi:10.1371/journal.pgph.0005697)
Supplement: S7 Text — (DOCX) [file pgph.0005697.s007.docx]

**Interviewer:** Alright, so, we are starting up.

**MP:** mmh

**Interviewer:** Firstly, I thank you for giving me your time to have this discussion with you,

**MP:** mmh

**Interviewer:** Mostly I want to hear your views about this approach involving group discussions in designing solutions for various problems. There is no wrong answer so feel free, I want to hear your opinions.

**MP:** Mmh

**Interviewer:** Firstly, I would like to know you, what is your occupation?

**MP:** I’m a pig farmer

**Interviewer:** Okay

**MP:** I do also several Jobs, I’m also a carpenter and I’m also a welder

**Interviewer:** Alright. We are starting. What do you know about antimicrobial resistance?

**MP:** This problem I never knew you it before attended the meetings which were organized by researchers at the Malawi Liverpool Wellcome Trust.

**Interviewer:** mmh

**MP:** That’s when I knew about resistance, so within the group discussions that’s when I knew that micro-organisms build resistance to drugs.

**Interviewer:** Okay

**MP:** So, I learnt that it is caused when you don’t complete your dose that you have been given at the hospital, and when you get sick again and use the same drugs again, you may not recover because the micro-organisms have developed resistance.

**Interviewer:** Alright.

**MP:** Sure.

**Interviewer:** Alright. In your understanding what challenges are caused to human healthy due to this issue?

**MP:** This issue brings health threats to human immunity

**Interviewer:** How about as a community, what challenges would be caused due to this issue?

**MP:** The community would be hugely affected because people may get sick often as a result, they may not be productive

**Interviewer:** How is this connected on the animal perspective?

**MP:** To animals sometimes we administer drugs without knowing the recommended quantity and sometimes even the feed which we give our animals sometimes is mixed with drugs and if we eat the animal which contain those drugs then still it will affect people’s lives.

**Interviewer:** How can we prevent this?

**MP:** This can be prevented firstly; we must be responsible to take care of ourselves and I should also thank the researchers for encouraging us to be in groups because in a group you get so many solid ideas which will assist the whole group. And what I loved the most was that they gave us the authority to discuss on our own because they also wanted to learn from us.

**Interviewer:** Alright, so you also mentioned about taking care of ourselves, taking care of ourselves in what way?

**MP:** To take care of ourselves what I mean by saying that is if we are sick and go to the hospital, if we receive drugs from the hospital, we must be responsible enough to finish the dosage which you have receive at the hospital. And don’t ask drugs from neighbors

**Interviewer:** Alright, you mentioned that you heard about this issue from the meetings, have you never heard of this problem previously?

**MP:** No, it was my first time to hear about it.

**Interviewer:** What was your experience in taking part in these discussions?

**MP:** It was very beneficial to me. It was very beneficial because I learnt some new things from colleagues which I never knew about. And my friends also learnt new things from me.

**Interviewer:** Alright, how about in terms of the time which you were spending there, wasn’t it disturbing your daily activities? And what’s your view on the venue?

**MP:** The place was fine. And on the time perspective it wasn’t disturbing with my time because I already knew about the program, so I was planning my daily activities according to that particular timeline.

**Interviewer:** You mean throughout the whole process it was fine?

**MP:** It was all fine, maybe the only time that it went wrong was when we received the chiefs because they didn’t start with us, they just joined us at the final stages.

**Interviewer:** Alright, so you are saying the chiefs joined you in later stages, what is your view on that arrangement?

**MP:** Yes

**Interviewer:** Was it fine for them to come on the final stages or you think it wasn’t good?

**MP:** No, it wasn’t fine with me. Because the chiefs are the leaders of our communities so if we are starting something in the community there is need to start together.

**Interviewer:** Maybe in the whole process what do you think should be changed or it should be done differently?

**MP:** I don’t see anything that should be changed

**Interviewer:** Okay

**MP:** Maybe the only change that can be included is that those seniors that came from Lilongwe came very late, if they were busy, they should have sent someone else to represent them so that we should be starting together. Because sometimes we ask questions so it would be good for everyone to be there when the questions are being asked.

**Interviewer:** Alright. Now, what was your interaction with the facilitators?

**MP:** It was very good. Because we were coming to a point where we were spreading into groups, I think they were 3groups, and within the groups we were discussing the same issue of the Bactria, so we would draw a tree diagram and we were finding the fruits of that tree. So, the facilitators were spreading into the groups for example one facilitator would be in one group and the other facilitator could be in the other group.

**Interviewer:** Do you think they were listening to your opinions?

**MP:** Yes, they were listening to us, I will be lying if I say they never listened to our opinions.

**Interviewer:** Alright. In terms of the messages which they were giving you, was it enough?

**MP:** Yes, the messages were enough

**Interviewer:** Was there any message which was hard to understand?

**MP:** No all the messages were clear

**Interviewer:** What would you change in the interaction that you had with these people?

**MP:** I don’t see anything that should be changed

**Interviewer:** Alright. Now what I want to ask is how was your interaction with the experts on the topic of antimicrobial resistance?

**MP:** It was a good interaction. Because they were also describing the issue according to how they learnt. But our interaction was good.

**Interviewer:** Okay. Do you feel like you have learnt anything new from these people?

**MP:** Yes, because I didn’t know a lot of things

**Interviewer:** Maybe what new thing did you learn from them?

**MP:** I learnt that if I have received drugs at the hospital, I must finish the whole dosage

**Interviewer:** Alright. How were they accommodating your opinions or ideas?

**MP:** They were accommodating all ideas, but our only request is that we should try to reach many people as possible because for us we have been trying to share the messages with friends, but we cannot reach out to the masses, within the group we agreed to use radios and megaphones to send these messages to the masses.

**Interviewer:** You have mentioned that you shared with other people. How was there reaction when you told them about it?

**MP:** Most of them they accept the messages. For example, I have a friend who sales pigs at the market, and I went to have a chat with him just after 2days after the meeting and he was surprised to hear about it, and he accepted the message and so many people have accepted the message

**Interviewer:** Maybe what were their questions or worries?

**MP:** Their only worry was on the numerous existences of groceries which are not authorized to be selling these drugs, but they are doing so.

**Interviewer:** Alright. Now, what are your views on the arrangement that you used to design the solutions?

**MP:** It was very good because like I said in a group you come up with more good ideas than doing it individually, and as a group we were able to debate on the solution and agree on one good solution

**Interviewer:** Alright, what didn’t you like about the process of designing the solutions?

**MP:** There wasn’t anything that I didn’t like. But the only solution that we spent much time debating was on the use of traditional herbs, there were some farmers who rely only traditional herbs such as nimu to treat their animals, so some were saying I t works so they stopped using the modern medicines, but some were saying it doesn’t work.

**Interviewer:** So, how were you coming to one agreement or decision?

**MP:** We were all coming together from the groups to form one bigger group where we were analyzing the solution to reach an agreement as one group.

**Interviewer:** Alright, now I want to hear your views on the arrangement that you had where you met various groups of people to overlook at the solutions that you have been discussing, what are your views on that?

**MP:** It was a good arrangement

**Interviewer:** Alright, now I would like to hear about the co-creation event that you had, what are your views on that?

**MP:** The event went on well, we received visitors from Lilongwe, we received the senior veterinary officer who was also coming in all of the events, and I should thank him for his responses because his responses were clear to everyone

**Interviewer:** Alright, so during that co-creation event, were you given a chance to participate freely?

**MP:** Yes, and we had a role play and I was one of the actors in the role play. I was Mr. Banda in the role play.

**Interviewer:** How about in terms of the time that was spent during that event and the venue, what are your views on those?

**MP:** The time was fine and the venue as fine, it wasn’t far from everyone, the place was closer to everyone. Because the venue was located alongside the road, so it was like at the center for everyone.

**Interviewer:** Now, I want to look at the solutions which you came up with, what are your views on these ideas?

**MP:** These solutions that we came up with need to be followed. Because some of the solution include that anyone who is selling drugs without authorization should not be selling the drugs because they may be selling expired drugs and they don’t have the expertise to guide the patient on how to take the drugs.

**Interviewer:** How feasible are these solutions to be implemented?

**MP:** They are feasible to be implemented in such a way that we as the citizens are the ones that are responsible to make sure that they are implemented.

**Interviewer:** Alright, what are your views on the challenges that you may incur on your solutions to fight against antimicrobial resistance?

**MP:** They were good solutions because everyone was trained on how we can tackle this problem

**Interviewer:** So, is it possible to deal with the problem using the solutions that you developed?

**MP:** Yes, and I was happy that we met as a group

**Interviewer:** Okay. What challenges do you anticipate meeting in trying to implement these solutions?

**MP:** The challenges would be there, because if this behavior of selling drugs in unauthorized groceries continue then this problem won’t end

**Interviewer:** why can make it impossible to end?

**MP:** The intervention of the government, because for those people to stop here will be need for government to use its powers to stop these people.

**Interviewer:** Alright. We are moving on. Now I would like to hear from you, according to your participation in this whole process, what have you done differently or what are you planning to do differently in your daily activities?

**MP:** A lot of things have changed since I attended these meetings. I would say when you are in a group you learn about the right decision because you share various ideas and agree on one solid idea.

**Interviewer:** So, from all those ideas that you got from the discussions, what are you doing differently or what are you planning to do differently? Or you haven’t changed anything?

**MP:** There is change, because at first when I feel like I’m sick I used to rush to take antibiotics but now when I feel sick, I consult a doctor first who then prescribes drugs for me. So, moving forward I will be consulting a doctor if I feel like I am sick rather than just buying drugs from the pharmacy.

**Interviewer:** How important is that on the issue of antimicrobial resistance?

**MP:** It is important in such a way that if the doctor has prescribed drugs, I will follow the drugs according to what I have been told by the doctor, if the doctor has given me antibiotics, I’m supposed to complete the dosage. Even when my child is sick, I need to make sure that he finishes the dosage, because there are some children who throw away the drugs.

**Interviewer:** Alright. We are at the very end of our discussion, but I would like to give you an opportunity to add anything that you feel like you left out in our discussions.

**MP:** I forgot to add that people were emphasizing that we should be using traditional herbs however some were still insisting that we should depend on the modern drugs. But some people even in China, we see it in TVs they use herbs to give to animals and why can’t we also do it? So, we find out that first there is need for to know the right amount of dose.

**Interviewer:** Alright. Thank you very much for your time

**MP:** Thank you
