# Supplementary material for: Co-creating community-driven solutions and policy priorities to address antimicrobial resistance through Responsive Dialogues: A qualitative evaluation from Malawi
Source: PLOS Glob Public Health. 2026 Apr 28;6(4):e0005697. doi: 10.1371/journal.pgph.0005697 (PMC13123971; doi:10.1371/journal.pgph.0005697)
Supplement: S8 Text — (DOCX) [file pgph.0005697.s008.docx]

**Interviewer:** Alright, so we are starting. Firstly, I thank you for allowing me to have this discussion with you, mostly I want to hear your ideas on what you have been discussing so feel free there is no right or wrong answer. Firstly, I would like to know, what is your daily occupation?

**FP:** I’m a chicken farmer so daily I do chicken business. I started with broilers chickens then I ventured into layers chickens, but I faced a challenge with the layer’s chickens because the company where I buy the chickens from sold me sick chicks, so now I have started keeping Mikolongwe Chickens.

**Interviewer:** Alright. Firstly, I would like to know, what do you know about antimicrobial resistance?

**FP:** I know that this is a problem which is also affecting animals whereby when you administer drugs to the animals either, pigs or chickens, the drugs fail to work yet the same drugs used to work in the same animals, so the micro-organisms build resistance to the drugs either in a body of a human or the animals to the extent that the drugs cannot work anymore

**Interviewer:** Alright. What challenges could be caused in animals or humans due to that issue?

**FP:**  If the animals die it means we would make a loss and the other challenge is that if people eat the affected animals the problem would be passed on to them as well, people will develop resistance just like it happened with the animals.

**Interviewer:** Okay

**FP:** So, there are several challenges because even the government would incur a loss

**Interviewer:** Alright, how about the community, what challenges would a community face due to this issue?

**FP:** If the affected animals are being sold in the community it means people from that community will also be at risk of contracting the problem and sometimes even when you throw the affected animals away, there are some people who still go and pick up those animals and eat them

**Interviewer:** Alright. So, how would we prevent this issue?

**FP:** The main preventive measure is to raise awareness so that people should know about this problem because some of these issues are preventable if people are aware of the problem.

**Interviewer:** Alright, where did you learn about these things?

**FP:** I learnt this when I attended a training at [community name]

**Interviewer:** Was it your first time to hear about it or you had heard about it before previously?

**FP:** I had heard about it from the Malawi Liverpool Wellcome Trust when they were conducting a study on the same issue. So, the problem happened to my chickens before, but I never knew about the problem at that time.

**Interviewer:** Alright we are moving on. Now, how did you see this approach of having group discussions about antimicrobial resistance?

**FP:** This approach was very good because we were able to share ideas and learn from other people on how they take care of their animals and we also learnt how big the problem is, for instance one of the participants also mentioned that the problem even affects vegetables

**Interviewer:** Okay. How about the way the process was organized for instance the time that you were getting there or the venue, how did you see it?

**FP:** There was nothing wrong with that, everything was fine.

**Interviewer:** Didn’t it disturb your daily activities?

**FP:** No, it was all good.

**Interviewer:** Alright, we are moving on. Now I would like to hear your thoughts on your interaction with the facilitators, how was it?

**FP:** The interaction was very good; they were leaving everything to us to discuss and come up with ideas so the interaction with them was very good.

**Interviewer:** Alright. How about in terms of the messages which they were giving you? Was it enough?

**FP:** Yes, it was enough

**Interviewer:** Okay, how about your ideas, how were they accommodating your ideas?

**FP:** They were accepting everyone’s idea just like you said it here that there is no wrong or right answer it was also the same with them, everyone’s idea was being welcomed by them.

**Interviewer:** Alright, what message was hard to understand when you were interacting with the facilitators?

**FP:** The messages were clear, only during the discussions that’s when we were able to identify challenges that we would incur in implementing the solutions

**Interviewer:** Alright. Is there anything that you would like to change in terms of your interaction with the facilitators?

**FP:** There isn’t nothing that should be change, maybe the only thing is that on time, the first day we started a bit late than we had agreed but later it was all going well.

**Interviewer:** Alright, how about your interaction with the experts, how was it?

**FP:** The interaction with the experts was very good they gave us some insights which we were doing wrong, and they gave us information.

**Interviewer:** Alright, how were the experts accommodating your ideas?

**FP:** They were accepting our ideas however some of the ideas that we suggested such as the use of traditional herbs was a bit challenging for all of us to be on the same page because the traditional herbs don’t have fixed measurements of its quantity that has to be administered so it is not reliable, but the farmers were saying that the traditional herbs are helpful.

**Interviewer:** So, how were you coming up with a conclusion when it happens like that?

**FP:** We were spending much time discussing the point until we come up with a solution for instance this example that I gave you we agreed that the experts will conduct research to find out the facts so that it should be authorized.

**Interviewer:** Alright. What would you like to change on your interaction with these experts?

**FP:** The experts should be coming in at a good time so that we can have enough time with them

**Interviewer:** Alright, so we are proceeding. Now, I would like to hear what are your views on the approach that you used to design the solutions?

**FP:** It was a good approach, but we were doing it in separate groups so sometimes we were all talking about similar things

**Interviewer:** What did you like the most about this approach of designing solutions?

**FP:** What I liked the most was that everyone’s idea was being heard and everyone was able to provide their ideas. And the good thing about the groups was that we were coming up with strong solutions because some of the solutions were common in the other groups as well.

**Interviewer:** Okay, what didn’t you like about the approach of designing the solutions?

**FP:** It was taking a lot of time

**Interviewer:** What would you like to change so that it should be progressing well?

**FP:** I feel like the problem should be discussed as one group so that when we reach a conclusion, we should be getting over it right away

**Interviewer:** Okay, we are moving on. Now, I would like us to talk about the co-creation event that you had, what is your view on this final stage?

**FP:** This final stage went on very well, but time was limited that’s why I said earlier that I wish we could have more time with the experts

**Interviewer:** Alright, so here you have talked about time how about in terms of the venue where you were meeting at?

**FP:** We were meeting at a church hall in [community name]

**Interviewer:** Didn’t you find any difficulties with the directions to get there?

**FP:** No, the directions were not difficult

**Interviewer:** Do you feel like you were given a chance to take part in the discussions there?

**FP:** Yes

**Interviewer:** So, you mentioned that the experts should have come earlier, is there any other group of people which you wish they could have been come earlier?

**FP:** Yes, the chiefs should have been coming earlier because some of the issues that popped up in the discussions it involved chiefs a lot

**Interviewer:** What do you mean when you say they were involved a lot?

**FP:** They were involved a lot in terms of dissemination of messages, the chiefs seemed to play a major role when it comes to that because they are able to bring people together at one place and they also play a role in forming by-laws

**Interviewer:** Alright, now I would like to understand your views on the solutions that you designed?

**FP:** These solutions are helpful if they are to be implemented

**Interviewer:** How feasible are they? Or how are they helpful?

**FP:** Like I said earlier if we are to raise awareness a lot of people will take responsibility to prevent this problem and even the consumers would take responsibility

**Interviewer:** Okay. What challenges do you think would be incurred if you are to implement these solutions?

**FP:** We would face challenges especially on farmers because if the general public knows that chickens have got this problem then business would go down

**Interviewer:** What could be another challenge apart from that that would hinder the fight against antimicrobial resistance?

**FP:** The other challenge is that for example someone mentioned that farmers sometimes are fully aware of the problem that the animal has but to avoid making a loss he or she decide to sale the animal for consumption instead, so there is need for government to take part, but it seemed difficult.

**Interviewer:** Alright, we are proceeding, what have you done differently or what are you planning to do differently in future in terms of what you learnt from the discussions?

**FP:** On my part as a consumer, I pay much a attention whenever I’m buying meat to be consumed, I just don’t buy meat anywhere. That is according to my consumer perspective

**Interviewer:** mmh

**FP:** On my side as well as a farmer I have learned not to depend much on antibiotics when treating animals and I have also learned that I shouldn’t just be buying drugs on my own, but I should be consulting recommended people

**Interviewer:** What challenges are you facing to make that possible?

**FP:** The challenge that I’m facing is hesitation from other people, when you tell them the dangers of misusing antibiotics they hesitate.

**Interviewer:** Alright, so who have you discussed the messages with so far?

**FP:** I have talked with several people, I have discussed it with my friends, I have discussed it at church, and I also had a training elsewhere and I also raised it up.

**Interviewer:** How long did it take for you to discuss with other people since you finished the conversation events approach?

**FP:** I think after 2 or 3months

**Interviewer:** How is people’s reaction when you explain to them about these issues?

**FP:** In general, it seems like people have been doing things wrongly due to lack of awareness so people are showing willingness to know more.

**Interviewer:** Alright, this is the end of our discussion, but if there is anything that you would like to add you can do so?

**FP:** I don’t have anything else to add

**Interviewer:** Alright, thank you very much for your time

**FP:** Thank you
