# Supplementary material for: Co-creating community-driven solutions and policy priorities to address antimicrobial resistance through Responsive Dialogues: A qualitative evaluation from Malawi
Source: PLOS Glob Public Health. 2026 Apr 28;6(4):e0005697. doi: 10.1371/journal.pgph.0005697 (PMC13123971; doi:10.1371/journal.pgph.0005697)
Supplement: S9 Text — (DOCX) [file pgph.0005697.s009.docx]

**Interviewer:** So, we are starting,

**FP:** mmh

**Interviewer:** thank you very much for accepting to take part in this discussion, there is no right or wrong answer, mostly I just want to hear your opinions.

**FP:** Alright

**Interviewer:** So, I would like to know you first, what is you occupation?

**FP:** Here at work or at home?

**Interviewer:** Here at work or if you do other jobs at home? I just want to know what you do daily basis.

**FP:** My main Job is here at work; I spend much of my time here

**Interviewer:** Okay

**FP:** Every morning when I get here, I do my daily duties

**Interviewer:** What are your duties here?

**FP:** I was recruited here as a hospital attendant and even on my payroll I receive as a hospital attendant but because sometimes you know a few things that’s why they assign other duties to you so that’s why I work here at the pharmacy.

**Interviewer:** Alright. So, we are continuing. I want to hear your views, what do you know about antimicrobial resistance?

**FP:** I know that when you are suffering from cough, it’s not every cough that we are supposed to take an antibiotic

**Interviewer:** Okay

**FP:** And the behavior of taking antibiotics every time when you are sick, it causes the micro-organisms to develop resistance to the drugs. And by the time you get seriously sick the drugs may have completely stopped working in your body.

**Interviewer:** Okay. So, what really causes this resistance?

**FP:** The cause of that is that there is a behavior of not completing the dosage that we have received at the hospital when we start to feel better.

**Interviewer:** Okay

**FP:** So, if you take the drugs that way you leave some micro-organisms alive. Then after a week or a month you start feeling sick again and when you take in the same drugs you will realize that the drugs have stopped working. And some people when they have cough, they rash to the drugstore with the drugs they want in mind, for example they have in mind that amoxicillin treats them better than Bactrim, so when they go to the drugstore they just ask for amoxicillin. Those kinds of things are what they are causing resistance in the body.

**Interviewer:** What would be the problems that you may come across towards people or animals on this issue?

**FP:** If a person is doing that behavior. Of not completing the dosage or buying drugs without the doctor’s prescription

**Interviewer:** mmh

**FP:** In future you will find that the person has fallen sick, but he doesn’t have any drugs that can treat him from the suffering which may brought problems to his house because if the person stays at the hospital for so long, it means money will be wasted, if the person was working or doing business it will all stop.

**Interviewer:** How about the community? What challenges would they come across due to this issue of antimicrobial resistance?

**FP:** The community would face problems with this issue, because if the farmers are misusing the drugs for example in chickens. So, if a person goes to buy that chicken and eat it, or for instance now adays are selling chicken portions which means the chicken will be eaten by several people as a result this issue of antimicrobial resistance would affect the community.

**Interviewer:** Alright.

**FP:** Sure

**Interviewer:** Now, in terms of prevention, what should people do to prevent antimicrobial resistance?

**FP:** They can prevent this by going to the hospital whenever they feel sick and consult with a doctor, and the person should get right prescription.

**Interviewer:** Okay

**FP:** And the farmers they shouldn’t misuse drugs, they should be seeking a veterinary officer to assist them on their farming.

**Interviewer:** Where did you learn that from?

**FP:** I learned about this when I went for a training of antimicrobial resistance. That’s where I learned about it.

**Interviewer:** Was it you first time to hear about it or you had heard about it before?

**FP:** I was just hearing about it but since it was just rumors, I was using the message wrongly, but when I went to attend the training now, that’s when I realized that I was even hurting myself.

**Interviewer:** Alright. So, I would like to understand what was your experience on the events that took place?

**FP:** I had a lot of good experiences because we were doing things ignorantly, but now I make sure that the patient gets the exact drugs that have been prescribed by the doctor. Because there was that behavior whereby if the prescriber knows that there is Bactrim, that means all the patients that are suffering from cough will receive Bactrim. But now due to that training I’m able to tell that not every cough requires an antibiotic.

**Interviewer:** Alright, how about the time that you spent at these events, what is your view on that?

**FP:** The time was fine with me because they told me in advance. They didn’t just surprise me.

**Interviewer:** How about in terms of the length of the day or maybe the venue?

**FP:** The time wasn’t a problem because the day was spared for the event, but in terms of the venue, the place was a bit far.

**Interviewer:** What did you like during your participation in these events?

**FP:** Group discussions are good because you share ideas with each other

**Interviewer:** Okay, how about what you didn’t like about the events?

**FP:** I cannot lie it was good

**Interviewer:** Alright, I can continue right?

**FP:** Yes

**Interviewer:** Alright, how was your interaction with facilitators?

**FP:** We interacted very well, because they were giving us opportunities to ask questions, and wherever we didn’t understand they were explaining it again.

**Interviewer:** Okay. Were they listening to you?

**FP:** Yes, they were listening to us.

**Interviewer:** They were not taking themselves as if they are superior to you?

**FP:** No, they didn’t even seem like they know the things. There wasn’t like passing or failing we were all on the same level.

**Interviewer:** How about in terms of the messages that they were giving you, do you think it was enough?

**FP:** Yes, it was enough, just that we never used to know about the things at first.

**Interviewer:** Was there any message which was hard to understand about antimicrobial resistance?

**FP:** During the first meeting it’s when I was finding it difficult to understand the problem but because we met several times that’s when I understood the problem of antimicrobial resistance.

**Interviewer:** If you can remember what was the message that was hard to understand?

**FP:** The message about AMR in general because I never knew that resistance could develop in a person’s body, I only knew about drug resistance.

**Interviewer:** Alright. Is there anything that you think should be changed in your interaction with the facilitators?

**FP:** No, like I said I didn’t see any problem

**Interviewer:** Now I want to know, how was your interaction with the experts on the issues of AMR?

**FP:** We also had a good interaction because they shared with us what they knew, and they also learned some things from us.

**Interviewer:** Okay. What did you learn from the experts?

**FP:** The same issue of avoiding suspension of treatment which has been prescribed to you by the doctor at the hospital

**Interviewer:** Do you feel like the expert was listening to your ideas?

**FP:** Yes

**Interviewer:** Were they giving you a chance to speak?

**FP:** Yes, whoever had something to say was being given a chance to speak

**Interviewer:** Okay

**FP:** Sure

**Interviewer:** Is there anything that you can change on your interaction with the experts?

**FP:** No

**Interviewer:** It was all good?

**FP:** Yes

**Interviewer:** We are proceeding. Now, what are your views on the process which you used to design your solutions?

**FP:** It was a good process because we were divided into groups. So, everyone was discussing how the problem is caused. And the solutions that we came up with include that we should approach everyone with these messages, in schools, we also had chiefs and the chiefs were told to tell people during funeral gathering that they should be going to the hospital when they feel sick and don’t just rush to the drugstore to buy drugs.

**Interviewer:** mmh

**FP:** So, the solution was to approach everyone with this message

**Interviewer:** So, what did you like or what didn’t you like with the process that you used to design the solutions?

**FP:** I will be lying to say there is anything that I didn’t like. But I liked the solution of approaching people with these messages either using health talks because if people receive these messages it can help to reduce the problem

**Interviewer:** Maybe what challenges would you incur in implementing the solutions that you identified?

**FP:** Okay. The first challenge was transport. Because for instance if we have a drama group which spread the messages through drama, the group cannot travel on foot from here to go and act to another place, so DHO should be providing transport to assist these people.

**Interviewer:** mmh

**FP:** The challenge was that usually in government they set plans but to fulfill those plans that’s when it becomes a problem. So, they are also a challenge to the solutions.

**Interviewer:** But you feel like the solutions are feasible?

**FP:** Yes, very feasible

**Interviewer:** Aright, I want to talk about the co-creation event, where you all came together in one group, how was that event?

**FP:** The event went on well, because we presented all the solutions that we came up with to the visitors that we had on the day.

**Interviewer:** mmh

**FP:** we had some visitors from Lilongwe who took all the idea that we presented.

**Interviewer:** How about in terms of the time that you spent there, or maybe the long duration of the day?

**FP:** It wasn’t long, because we were knocking off at a good time

**Interviewer:** It didn’t interrupt your work?

**FP:** No, because I had already asked for permission at work

**Interviewer:** Alright. Did you feel that you were given a chance to take part in the discussions?

**FP:** Yes, I was given a chance because like I said we were in groups and all our ideas on the groups were being used.

**Interviewer:** Alright. So, you mentioned that you received visitors from Lilongwe, right?

**FP:** Yes

**Interviewer:** What do you think about that arrangement that some people should be joining you when you have already covered the other previous events?

**FP:** The visitors from Lilongwe have joined us in two events, they came on the first event when they explained to us about their role and on the final day they also came, so it was okay

**Interviewer:** Alright. How about the chiefs? What are your views on the arrangement that was made that the chiefs should join on the final day?

**FP:** That part was wrong, the chiefs should have joined us when we were starting the meeting

**Interviewer:** Okay. So, we are moving on.

**FP:** mmh

**Interviewer:** So, depending on your participation in the events especially the final event, is there anything that you have done differently, or you are planning to do differently?

**FP:** Yes, I’m telling my work colleagues about this issue, so slowly by slowly some people are understanding it but others still don’t understand

**Interviewer:** How about you personally, what are you planning to do differently? Either here at work or at home on your daily basis?

**FP:** I will do differently especially at home, because I had a bad habit of taking drugs here and store them at home so that if my child gets sick when I’m at work he can take the medicine at home, but now I make sure that he gets prescription first.

**Interviewer:** What is the importance of doing that?

**FP:** It is important because it for the good of your own health

**Interviewer:** Okay. What challenges would you come across in trying to do this?

**FP:** Misunderstanding with people, we are different people, to reach the extent where a person has understood a thing it is difficult, some people understand things easily than others

**Interviewer:** Alright, have you discussed what you gained from your participation in the conversation events?

**FP:** Yes, I have shared with people some of the are my work colleagues, because when I returned here, they asked me how it was, and I explained to them

**Interviewer:** Okay, so when you discuss with them what questions were they asking?

**FP:** They were showing willingness to know more about how this issue works

**Interviewer:** Alright. We are at the end of our discussions; I don’t know if there is anything that you would like to add from what we have discussed because maybe there could be something that you forgot and you would like to add?

**FP:** I just want to add that learning never ends so if we would organize more of these events so that other people should also learn it could be very helpful.

**Interviewer:** Alright. Thank you very much for your time.

**FP:** Thank you.
