# Supplementary material for: Co-creating community-driven solutions and policy priorities to address antimicrobial resistance through Responsive Dialogues: A qualitative evaluation from Malawi
Source: PLOS Glob Public Health. 2026 Apr 28;6(4):e0005697. doi: 10.1371/journal.pgph.0005697 (PMC13123971; doi:10.1371/journal.pgph.0005697)
Supplement: S10 Text — (DOCX) [file pgph.0005697.s010.docx]

**Interviewer:** We are starting up. Thank you for your acceptance to participate in this interview, mostly I just want to listen to your experiences on takin part in the conversation events, so feel free. Firstly, I would like to know what is your daily occupation?

**FP:** I’m a nurse by profession, I work at a private pharmacy so that’s what I do.

**Interviewer:** You can take off your mask if you feel like it at least we are seating outside.

**FP:** Okay. So, that’s what I do, and it’s not always that I’m always at the pharmacy but when there are any other health related jobs I go to work there.

**Interviewer:** Alright. So, firstly I would like to understand that what do you know about antimicrobial resistance?

**FP:** They are microbes which have gained resistance to drugs or to treatment which was previously being treated with a certain antibiotic but upon gaining resistance it can no longer be treated with that antibiotic

**Interviewer:** Okay. Maybe what are the causes of AMR?

**FP:** First, AMR is caused by wrong treatment, I don’t know how I can explain it

**Interviewer:** You can explain anyhow

**FP:** Okay, because in government health facilities we don’t do blood culture to know the exact bugs that are causing a particular infection

**Interviewer:** Okay

**FP:** so, as a result we just depend on try and error, so I can say wrong or mismanagement of the condition

**Interviewer:** Okay

**FP:** And our habits also contribute to it because sometimes we just take antibiotics for no reason, for instance a person may use antibiotics just because he has flue

**Interviewer:** Okay

**FP:** And things like those cause resistance

**Interviewer:** Okay

**FP:** And the way we handle antibiotics in the hospitals we are not much careful as a result the microbes are gaining resistance because they have already been exposed to that, so they are mutated to go in another form to avoid being attached. I can say so.

**Interviewer:** Alright. What challenges would people or the community would face due to AMR?

**FP:** We would have chronical illnesses

**Interviewer:** Okay

**FP:** Because there will be no drugs to cure a person as such a person will be sick for a long time than to do productive things in his life which would lead to poverty in the community and as a family.

**Interviewer:** mmh

**FP:** And death rate would also increase because the patient may need an antibiotic which he is resistant to as a result the patient may die.

**Interviewer:** mmh

**FP:** And our cost of living would also increase because we would aim to buy an expensive antibiotic to treat those microbes

**Interviewer:** Alright, so, how can we prevent this?

**FP:** if a person gets sick, firstly, he should go to the hospital and consult with a doctor, before starting treatment we need to take samples such as the culture to know the exact antibiotic that the patient must receive, and we shouldn’t be misusing drugs.

**Interviewer:** where did you learn about that from?

**FP:** I learnt about that in class from school, I also learnt about this at the hospital ward, and I also learnt about this at this training.

**Interviewer:** Okay, overall, how was your experience in taking part in these events?

**FP:** It was good because initially we take it as unimportant issue but when I took part in the events I was alarmed and since then I’m cautious when taking antibiotics

**Interviewer:** Cautious how?

**FP:** I only take the antibiotics when I’m seriously sick and when the doctor has prescribed those drugs for me. I stopped the habit of taking antibiotics anyhow.

**Interviewer:** Alright, how about your experience on the time that you were spending in these events? Maybe duration if it was long, or regarding the venue?

**FP:** I cannot say it was long in terms of time duration because it was depending on our activeness, if we are active, we were finishing at a good time, and overall, I would say time was alright we were not having prolonged sessions.

**Interviewer:** Okay, was it the same in all the events?

**FP:** Yes, in all the events

**Interviewer:** Alright, how about in terms of the venue?

**FP:** alright, at first, I wasn’t very comfortable with the venue but on the final day it was very wonderful

**Interviewer:** What do you mean you when you say at first you weren’t comfortable?

**FP:** Alright at first, we were having the discussions in [community name] at some hall, and the whole was huge, but we understand because they told us that Malawi Liverpool welcome Trust hold their activities in the community but to be honest during the co-creation events the venue was very nice.

**Interviewer:** Did you learn anything new from there about AMR?

**FP:** To me it wasn’t something new as such because I already had some knowledge from class, the only new thing to me was that farmers are also using antibiotics, I didn’t know about this, it was news to me.

**Interviewer:** Alright. Overall, what didn’t you like about the whole process of the events?

**FP:** The question though

(Giggles)

**Interviewer:** Or let’s start with what you liked, what did you like?

**FP:** I liked the place, it was quite good

**Interviewer:** Okay

**FP:** And apart from the conversation events we were also meeting people and we were making friends, so it was good.

**Interviewer:** Okay

**FP:** And they treated us with respect, and we were all just uniform, and the researcher were all part of us, and we were all sharing ideas

**Interviewer:** What didn’t you like or what should be changed?

**FP:** almost everything was good

**Interviewer:** Alright we are moving on

**FP:** Alright, if there is something that can be changed is that the first events, they should be choosing at least a better venue

**Interviewer:** What kind of better place is that, would you describe it?

**FP:** It should be somewhere interactive because at that hall it was just us and it was very quiet as if we are at a church.

**Interviewer:** Alright so we are moving on. I want to hear from you, in terms of your interaction with the people that were organizing these events, what was your experience?

**FP:** In terms of what part?

**Interviewer:** The facilitators?

**FP:** They were good, they know their job, they were keeping truck of time. They were having a program that was guiding us through.

**Interviewer:** Okay

**FP:** They were good facilitators

**Interviewer:** Okay, do you feel that they were listening to you?

**FP:** I would say 90% of the contributions were coming from us

**Interviewer:** Okay

**FP:** They were just facilitating

**Interviewer:** How about in terms of the information which they were giving you, was it enough?

**FP:** It was enough because wherever we don’t understand we were asking them Questions and they could explain what they mean to us

**Interviewer:** Was there any information that was difficult to understand?

**FP:** To me I didn’t find anything difficult to understand but since there are different groups of people, so some people find it difficult to understand

**Interviewer:** Okay, would you change anything in terms of your interaction with the facilitators?

**FP:** It was fine because we were discussing in groups.

**Interviewer:** Alright, how about your interaction with the experts?

**FP:** which ones were the experts?

**Interviewer:** Didn’t you receive a certain group of people who were being taken as experts on these matters of AMR?

**FP:** Those ones to me I feel like they were always rejecting the solutions that we came up with, they were always challenging the solutions that they cannot be implemented, and others were saying they are long term solutions

**Interviewer:** Okay

**FP:** But still more I found it more important because they were assisting us to think much better on how best we can design our solutions

**Interviewer:** Okay

**FP:** Because to some people they felt like the experts were just arguing on every solution

**Interviewer:** Okay

**FP:** So, maybe our facilitators should be explaining to us in advance on what the expert expects from us.

**Interviewer:** Okay. What did you learn from the experts?

**FP:** Alright. What I learnt was that for some of the things to be implemented they require political will, so there is need to influence the political leaders.

**Interviewer:**  Alright, did you feel that your voice was heard when you had a discussion with the experts?

**FP:** Yes, our voice was heard because some of the solutions were even being accommodated by them during the event, they could tell us that this solution we will take it to the DHO to do it. So, some of our solutions were being heard

**Interviewer:** Alright. We are proceeding, I wanted us to talk about the process that you used to design various solutions that you came up with, how was your experience? How did you find the process of designing solution?

**FP:** For us to come up with a solution first we were having a problem, and before finding solutions we were identifying the root causes of our problem first

**Interviewer:** Okay

**FP:** So, after identifying our root causes were identifying the effects which would result due to the problem

**Interviewer:** mmh

**FP:** And after finding out the causes and the effects, then we were finding the solutions

**Interviewer:** Okay. What are your views on the whole process?

**FP:** It was a good process to me because it was easy to understand even a layman would understand

**Interviewer:**  What did you enjoy about the whole process of developing solutions?

**FP:** It was good because it was easy to understand and to follow because we could have a cause, effect and then we were moving step by step to see how we can deal with it.

**Interviewer:** What didn’t you like about the whole process of designing the solutions?

**FP:** Some of the solutions we were just wasting our energy because it can never happen because most of the things need political will

**Interviewer:** Alright. Is there anything you would change about developing solutions?

**FP:** No, they can continue the same way.

**Interviewer:** It was all alright?

**FP:** Yes, but we should just add that these are long term, and these are short term solutions, and we should also add that these are probable.

**Interviewer:** Okay. So, I want us to talk about the co-creation event, how was this whole process?

**FP:** The co-creation event with the experts it was good, and the experts were giving us an overview of our discussions and they were bringing in other new ideas to think about on our solutions. So, we could feel that they also took part in developing solutions.

**Interviewer:** Okay

**FP:** yah

**Interviewer:** How about in terms of time commitment?

**FP:** It was good because it was half day

**Interviewer:** Okay, how about in terms of venue?

**FP:** The venue was perfect

**Interviewer:** Alright. Did you feel like you were included in the discussions that were taking place?

**FP:** Yes, I was included 100%

**Interviewer:** You were able to speak freely?

**FP:** Yes, I was able to speak, and I presented.

**Interviewer:** Okay, so you said that you received other people who you didn’t start together with right?

**FP:** Yes, the chiefs

**Interviewer:** what do you think about other stakeholders joining you later?

**FP:** I feel like they should be coming at the beginning of the events and at the final event

**Interviewer:** Why?

**FP:** Because the farmers came on the day that we were prioritizing our solutions so they couldn’t know more about what were discussing because on this day we were sort of just concluding everything that we have been discussing in our previous events. And mostly we were focusing on solutions.

**Interviewer:** Okay. So, what did you think about the solutions which you identified?

**FP:** What do you mean?

**Interviewer:** As in were they feasible?

**FP:** Yes, they are but it needs commitment to achieve this starting from health service providers up to the community, so they are feasible if we all commit ourselves to it.

**Interviewer:** do you think they will be successful in dealing with AMR?

**FP:** Yes, they can be successful

**Interviewer:** What challenges are you anticipating when implementing these solutions?

**FP:** Usually government hospitals depend on donations to receive drugs, which means that if the donation is delayed or there is no donation at all it means the problem would continue, because for us we are business oriented we just sale to make money

**Interviewer:**  Okay. So, going forward based on your involvement on a co-creation event, what are you planning to do differently or what have you done differently so far?

**FP:** So far ai have stopped sharing antibiotics with all my friends that depend on me to share them antibiotics

**Interviewer:** why is this important for you to do?

**FP:** It is important because I’m saving them

**Interviewer:** So, what challenges are you facing?

**FP:** People think I’m being selfish because I’m not assisting them

**Interviewer:** Alright, so who have you spoken to about the information which you acquired during the process

**FP:** My family members, my friends and my fellow health workers

**Interviewer:** When did you speak to these people?

**FP:** I started talking to them about it the moment I started taking part in this

**Interviewer:** What was their response or what were the questions?

**FP:** They only asked about when I realized about this issue, but so far people seem to understand when I tell them

**Interviewer:** Alright, we are towards the end of the discussion, I would like to give you a chance to add whatever you would like to add?

**FP:** No, a lot of things have been discussed.

**Interviewer:** Alright, thank you very much for your time

**FP:** Thank you
