# Supplementary material for: Co-creating community-driven solutions and policy priorities to address antimicrobial resistance through Responsive Dialogues: A qualitative evaluation from Malawi
Source: PLOS Glob Public Health. 2026 Apr 28;6(4):e0005697. doi: 10.1371/journal.pgph.0005697 (PMC13123971; doi:10.1371/journal.pgph.0005697)
Supplement: S11 Text — (DOCX) [file pgph.0005697.s011.docx]

**Interviewer:** Alright, so we are starting.

**MP:** Yes

**Interviewer:** Firstly, I would like to thank you for giving me your time to have this discussion with me. You should feel free, there is no wrong answers in this discussion, mainly I just want to hear your views involving antimicrobial resistance. So, I would like to know you, what is your occupation?

**MP:** I am working as a nurse and I also run a business, I have a medicine store.

**Interviewer:** Okay

**MP:** That’s what I do to get all the basic needs

**Interviewer:** Alright

**MP:** Sure

**Interviewer:** So firstly, I want to know what is your understanding about antimicrobial resistance?

**MP:** Maybe because of the knowledge that I acquired from school and because of the job that I’m doing, I understand pretty well about antimicrobial resistance because I have seen people being affected by antimicrobial resistance, for example some TB patients fail to recover from the drugs or they develop resistance and that is caused because they were skipping the dosages.

**Interviewer:** Alright, what problems would that cause in people and in animals?

**MP:** This is a big problem, let me start with the animal part

**Interviewer:** mmh

**MP:** If an animal has that problem of antimicrobial resistance and if people eat the meat of that animal the problem may be transmitted to them

**Interviewer:** Okay

**MP:** For example if a farmer was administering Bactrim to the animal and the animal has now developed resistance to the drug, it means that if I eat that animal and when I get sick in future and take Bactrim I may not recover from it

**Interviewer:** Okay

**MP:** So, it’s a big problem because it can be transmitted from the animals to people who eat that animal as a result people may also not recover from the sickness because they will develop resistance

**Interviewer:** Alright. How about to the community, what problems would it cause to the community?

**MP:** Currently people in the community don’t know of this problem

**Interviewer:** Okay

**MP:** So, as a result the problem will spread widely in the community and we will end up having a lot of people with this problem in that community

**Interviewer:** Okay, how can we overcome this problem?

**MP:** The first thing that should be done to overcome this problem is to teach people about the positive and negative effects of antibiotics

**Interviewer:** Okay

**MP:** They should know when they are supposed to take antibiotics, because if you go into the community right now even a small child would describe how amoxicillin looks like and this is because they are easily accessed and they are found in their homes.

**Interviewer:** mmh

**MP:** So, every time when they get sick they rush to take antibiotics, so the first thing that needs to be done is to teach people.

**Interviewer:** Okay

**MP:** The easiest way of doing that is through the hospital. Every time at the hospital they do a health talk. So, people may be taught about antibiotics through those health talks

**Interviewer:** mmh

**MP:** And from there then we can use radio stations, then we can use TV stations, we can also use posters and from there we can also use free music concerts where we can share information about antibiotics

**Interviewer:** Alright. Where did you learn about all this?

**MP:** Some of them I have learnt from school but most of it I learnt it from the conversation events that we have been having, and we learnt about new things from those meetings.

**Interviewer:** Okay, what were those new things that you learnt from there?

**MP:** One of the new things that I leant from there was that as a nurse I would also contribute to antimicrobial resistance depending on how I’m dispensing drugs

**Interviewer:** Okay

**MP:** In such a way that am I dispensing the right drugs for that particular time

**Interviewer:** Okay

**MP:** And the second new thing that I learnt there was that we are not always supposed to take antibiotics every time when we feel like we are sick. When we are sick we should be seeing a doctor and we should respect what the doctor has prescribed for us.

**Interviewer:** mmh

**MP:** We also learnt that we have a behavior of storing drugs in our homes which is not a good behavior

**Interviewer:** mmh

**MP:** So, those are some of the new things that I learnt from there

**Interviewer:** Okay

**MP:** Sure

**Interviewer:** So, how was your experience in these conversation events?

**MP:** Okay. Apart from the fact that I’m in a business of selling medicines, I feel that I have learnt a lot of important things and it has made me change some of the ways on how I do things in such a way that when we have a client our main focus shouldn’t just be on the money but we should focus on how we can assist the client.

**Interviewer:** Okay

**MP:** So it looks like I increased my experience on this and even my approach to my colleagues concerning the issue of antibiotics has risen

**Interviewer:** Okay. How about in terms of your time commitment, how did you see it?

**MP:** The time wasn’t a problem because we were starting at a good time and we were finishing at a good time. And I also made a plan when going there, I had somebody that was looking after my business, I just made sure that the person that is looking after the business should be a nurse and not just anybody.

**Interviewer:** Okay

**MP:** So, there wasn’t any problem

**Interviewer:** How about in terms of the venue?

**MP:** At first we were expecting that they will take us to a very good place for instance we thought they will take us to Mulanje

(giggles)

**Interviewer:** Out of Blantyre?

**MP:** Yes, so we asked them about that and they answered very well, they told us that we are working with communities and we like to have these discussions with in a community based venue as a neutral venue.

**Interviewer:** Okay

**MP:** So it was well understood by us. So, it wasn’t a problem

**Interviewer:** Didn’t you find it difficult to locate the venue?

**MP:** The venue was easy to locate for a person that doesn’t have challenges in understanding map

**Interviewer:** Alright. Now, from the way the whole process was organized, is there anything that you liked and disliked?

**MP:** I liked it because it wasn’t teaching as but it was more like a participatory approach, we were all being involved and sometimes they were even putting us in groups. The facilitator in our group wasn’t dominating he was letting to be open to give our ideas.

**Interviewer:** If there is anything that you would change on the way it was organized?

**MP:** The only thing to change is the venue

**Interviewer:** Only the venue?

**MP:** Yes, the venue

**Interviewer:** How do you want the venue to be like?

**MP:** They should be finding a good venue, at least a lodge maybe

**Interviewer:** Alright

**MP:** Yes, to me it’s just the venue, but everything was fine, it was well organized and well presented.

**Interviewer:** mmh

**MP:** Let me explain briefly what was happening, when we arrive at the venue we were being welcomed by a person who was sanitizing our hands, then he was giving us masks, and we were signing in a register to show that I have come, then there was some sweets and we were getting those, then we were going to the chairs, they were already putting two bottles of water there sometimes three bottles and then we would start chatting

**Interviewer:** Okay

**MP:** When it’s time to start our session, somebody was opening with a prayer and we were having tea break and lunch break

**Interviewer:** mmh

**MP:** So, it was well organized and I believe that if they are to call us back to have another discussion I don’t think there would be anyone that wouldn’t want to come

**Interviewer:** Alright

**MP:** Unless if the person is committed to something else then it can be another issue but from how the events were organized I believe everyone would want to come again.

**Interviewer:** Alright so we are proceeding. I want us to discuss how the conversation events were going. Firstly, I want to know how was your interaction with the facilitators?

**MP:** Like I said earlier, they were putting us in groups, so each group was having one facilitator. So our facilitator, he was making sure that everyone puts in their ideas.

**Interviewer:** Okay?

**MP:** In so doing he was making us to feel free, because he was not dominating. It’s unlike when facilitators are dominating we just end up listening to them without contributing our ideas, but as for him, he was leaving the discussion to us. And we would even laugh with them. So, to me I had no problem with the facilitators.

**Interviewer:** Alright. So, in terms of information, do you feel like they were giving you enough information?

**MP:** Okay, so it was like they were guiding us, normally it was a discussion, not teaching as such.

**Interviewer:** mmh

**MP:** So, within the conversation somebody would throw a question, for instance he would ask how do you think we would deal with the problem of antimicrobial resistance, so in so doing we were giving each other a chance to put in ideas

**Interviewer:** Were there any challenges in understanding the information that you were sharing?

**MP:** Okay, they were allowing us to ask questions if we don’t understand the question.

**Interviewer:** mmh

**MP:** And people were asking questions, that chance was there. And sometimes it was happening that the question was being answered within the group and not just by facilitators only.

**Interviewer:** Is there anything you would change in terms of your interaction with the facilitators?

**MP:** To me I was free with them, even at lunch time we would have a chat and we would talk about the conversations. So, we had a good interaction.

**Interviewer:** Alright. Let’s proceed. So, I would want to hear about your interaction with the experts, how was it?

**MP:** From what I remember on the first day we received experts from Lilongwe

**Interviewer:** Okay

**MP:** But the problem we had with them was that they arrived when we were almost at the end of the program. They said the problem was that they started off late in Lilongwe.

**Interviewer:** mmh

**MP:** But they also had their time to talk to us

**Interviewer:** mmh

**MP:** But when we went to the hotel on the last two days that’s when I was too close with them

**Interviewer:** mmh

**MP:** That’s when one of the experts asked me that are you sure that if we got to a local market right now we will find people selling antibiotics at a market?

**Interviewer:** mmh

**MP:** And I said yes, because what happen is that those vendors they sale along the borders, so it’s difficult to stop them because they keep on saying they are not doing it in our country. And it is the same at [border area] people are selling antibiotics like fish.

**Interviewer:** Okay

**MP:** But they do it within the borders of Mozambique

**Interviewer:** mmh

**MP:** So, we interacted

**Interviewer:** Do you feel like you learnt new things from these experts?

**MP:** Yes, especially when one of the experts started to give us the figures of people that are resistant at the moment, so I was shocked to hear the figures. That’s when I realized that this is a very big problem because the figure is huge.

**Interviewer:** Did you feel your voice was heard, were they taking your ideas?

**MP:** Yes they took our ideas. I’m saying they did because on the last day when each group was presenting their ideas to the visitors who came on the day. During the presentations you would actually see your idea on the slides.

**Interviewer:** mmh

**MP:** So, they took our ideas because it’s also what was presented on the last day.

**Interviewer:** Okay. Is there anything you would change in terms of your interaction with the experts?

**MP:** No, it was all good.

**Interviewer:** Alright let’s proceed. Now, I was us to talk about the process that you used in developing the solutions. How did you see that process?

**MP:** The process was good and it wasn’t time consuming, because we were divided into groups and everyone would discuss their topic, and we would bring everything discussed in the groups together and then we would discuss them all of us together.

**Interviewer:** Okay

**MP:** So, in so doing we covered a lot of things in short time.

**Interviewer:** Is there anything you particularly liked or disliked on the process that you used in developing the solutions?

**MP:** I liked it because when we were divided into groups we were able to cover more topics in a short time.

**Interviewer:** Alright. Is there anything you would change on the process of developing solutions that you used?

**MP:** No

**Interviewer:** No?

**MP:** Yes

**Interviewer:** Alright, So I want us to talk about the co-creation event, the last event, how did you see it?

**MP:** They last event was good, and not because of the venue only, since it was a hotel

(Laughter)

**Interviewer:** Okay, so we will include the venue it was good. Okay

**MP:** Okay, last event was good because we were like giving feedback on what we have been discussing, so we had well developed ideas that we presented and ideas that would be feasible

**Interviewer:** Okay

**MP:** So it was a good time because we met intelligent people

**Interviewer:** How about in terms of time commitment or the venue?

**MP:** To me like I said earlier I did not have any problem with the time. If I’m not wrong, we were staring at 8:30am and we were finishing around 9:30am, if I’m not wrong. So, to me it was already in my plans.

**Interviewer:** Okay

**MP:** And I did not have any problem with the venue. They communicated to us during the previous meeting about the venue of the co-creation event, so what I did for me is I had to go there and checkout the location of the venue in advance before the day of the event

**Interviewer:** Okay

**MP:** I had no problem with the venue. And it was a hotel.

**Interviewer:** How about in term of your participation in the events, how do you feel about it? Were they giving you a chance to participate?

**MP:** Yes, and I am one of the people who spoke a lot during the events

**Interviewer:** Okay

**MP:** So, the opportunity to participate was there and everyone would agree that we were given a chance to speak.

**Interviewer:** Okay

**MP:** I was like what you said at the beginning, there was not right or wrong answer. So, people were free to speak. And during the first meeting there was recording and taking pictures, so people thought it will be on Air and their bosses will hear them talk, but we recognized that everything was confidential so people started to open up.

**Interviewer:** Alright. I heard that there were some stakeholders who joined you at the co-creation event such as the chiefs, you also mentioned of the visitors from Lilongwe, what do you think about this?

**MP:** The chiefs participated with the previous group of participants so they were aware of the issue, but they joined us on first day of the co-creation event and they also shared their ideas from the previous group.

**Interviewer:** Alright, we are proceeding. What do you think of the solutions that you identified?

**MP:** Personally I think the solutions were good, and I know the government wouldn’t take all of the solutions but I’m sure they will take some of them, because those solutions are coming from people working on the ground

**Interviewer:** mmh

**MP:** However some of the solutions may hurt other people

**Interviewer:** okay, what do you mean by that?

**MP:** For instance some of the solutions may affect businesses and if some of the people that are going to implement these solutions are involved in this business they may ignore the solutions. It will just depend on how people will accept them but they are good solutions.

**Interviewer:** Okay. How feasible are these solutions, do you think it can be possible to implement them?

**MP:** It’s possible to implement them but some cannot be implemented. I will go back to say that they are possible to implement if the government is willing to implement them, because in government it’s all about politics, I will give an example of the motorbike taxis which are everywhere in the cities, but the government is failing to control them. So some of the solutions may not be feasible due to politics

**Interviewer:** How feasible are these solutions in dealing with antimicrobial resistance problem?

**MP:** They are good solutions because this is a big problem and if we don’t control it in four to five years to come this problem will be among every family as a result government will lose money in procuring drugs.

**Interviewer:** mmh

**MP:** Because if you noticed the government stopped procuring antibiotics such as Penicillin and that is because people developed resistance to those antibiotics and if you think about it you will realize that government had already lost money in procuring those drugs.

**Interviewer:** Okay, what challenges do you anticipate in implementing these solutions?

**MP:** The challenge that I am anticipating is that most of those solutions are affecting people who sale drugs

**Interviewer:** Okay

**MP:** To say that most of the times people that sale drugs to people they sale them without a prescription, for instance if I go to town now, I will be able to buy drugs without any problem

**Interviewer:** mmh

**MP:** So, to me I feel like it will be difficult for people who sale drugs to accept these solutions

**Interviewer:** Alright. Who have you discussed with about the information that you acquired from these conversations?

**MP:** Most of the people that I have talked to are my customers

**Interviewer:** mmh

**MP:** So, I tell them about the negatives and positives of antibiotics

**Interviewer:** mmh

**MP:** I have also talked to my relatives about it

**Interviewer:** Alright, what was the reaction of the people that you have talked with?

**MP:** It’s not everyone that would accept it, some people still ignore it and say it get to pass like other problems.

**Interviewer:** They are taking it as a small problem?

**MP:** They are not taking it as a problem at all

**Interviewer:** Alright, it looks like we are going towards the end, I want to give you a chance if you have any last words that you forgot to bring it up you would speak up?

**MP:** I just want to add that whosoever thought of coming up with the idea of doing the conversation dialogues came up with a very brilliant idea and government should consider the solutions that we came up, they can polish them up but they should consider them so that we should reduce the problem of antimicrobial resistance.

**Interviewer:** Alright, thank you very much it looks like we are at the end.

**MP:** Thank you.
