# Supplementary material for: Co-creating community-driven solutions and policy priorities to address antimicrobial resistance through Responsive Dialogues: A qualitative evaluation from Malawi
Source: PLOS Glob Public Health. 2026 Apr 28;6(4):e0005697. doi: 10.1371/journal.pgph.0005697 (PMC13123971; doi:10.1371/journal.pgph.0005697)
Supplement: S12 Text — (DOCX) [file pgph.0005697.s012.docx]

Interviewer: Alright, firstly, I would like you for accepting to have this interview with me. Before we start, I would like to know, what is your daily occupation?

MP: I work at [health facility]

Interviewer: Who do you work as here?

MP: I work here as a pharmacy clerk

Interviewer: So, firstly I would like to know that what do you know about antimicrobial resistance?

MP: I know a couple of things when it comes to that part,

Interviewer: Okay

MP: firstly, what I know is that a lot of people are suffering from the issue of antimicrobial resistance because the drugs that we were depending on when people get sick, they are no longer working

Interviewer: Okay, how is that caused?

MP: Okay, this is caused because many people don’t complete the dosage that has been prescribed for them by a doctor at the hospital as a result, they cause resistance

Interviewer: What challenges are cause due to this in people or in animals?

MP: There are several challenges that people can face, the first one is that people can lose their lives, secondly it would increase poverty in the country because if the drugs are weak then there is need to buy more drugs which would be expensive to buy.

Interviewer: Okay, how about in terms of the animals, how does it relate in the animal perspective?

MP: In animals we are also being affected because most farmers are using antibiotics in animals. And some of the drugs which they administer to the animals are human drugs, so those drugs that have been administered to the animals can be transferred to us if we say we are to buy and it that meat

Interviewer: So, what can people do to overcome these problems?

MP: These problems can be overcome in several ways, but the main way is to teach people about antibiotics and their effects, people shouldn’t also buy antibiotics in markets without proper documentation but what is needed is that when a person is sick, he or she should be going to the hospital to get a prescription

Interviewer: Where did you learn about this?

MP: I learnt this from the Malawi Liverpool Wellcome Trust

Interviewer: Was it your first time to hear about it or you heard about it before?

MP: I was hearing it but here and there, but when I went for the conversation events that’s when I knew about it in details.

Interviewer: What was your experience for taking part in these discussions?

MP: I gained a lot of experience because I learned about new things which I wasn’t aware about. I now know how dangerous it is to misuse antibiotics, so I have a lot of experience

Interviewer: Alright, how about your views on the time that you spent there, or maybe the venue of the place, what are your views on that?

MP: During the first 3events I would say the venue wasn’t good, because we were doing the conversation events at some church hall. So, the place was good

Interviewer: Okay, in what way?

MP: The whole that we were using wasn’t nice, but during the final stages we had two events which took place at a hotel and the place was very nice and everything was very nice

Interviewer: When you say it was nice what are you looking at? What did you want to fix about the other place?

MP: It was a hot season, but we didn’t have fans, so it was very hot, the windows of the hall were not fine

Interviewer: did you encounter any challenges to get to the place?

MP: Yes, the place was in a hidden location so to get to the place we were struggling a bit with the directions

Interviewer: alright depending on your participation in the whole process of the conversation events, what did you like or what didn’t you like?

MP: what I liked was that I gained knowledge first

Interviewer: Okay

MP: secondly, I visited a place where I have never been before at the hotel

Interviewer: Okay

MP: Now what I didn’t like was that, first the allowance wasn’t enough and secondly, we needed to receive T-shirts that we could put on when giving health talks at the health facility because there are other people who can read and if wear those T-shirts then those people would be interested to know about antibiotics

Interviewer: Okay. So, we are moving on.

MP: mmh

Interviewer: I would like to hear your views on your interaction with the facilitators?

MP: To say the truth our facilitators were very good people, and everything went on very well, they were giving us a chance to ask questions and when we have a point to share, they were also giving us that opportunity to explain

Interviewer: Okay, were they listening to you when you present your ideas?

MP: They were listening to us. They were listening to everything. They were actively paying attention and everything was going on very well

Interviewer: Okay?

MP: Sure

Interviewer: How about in terms of the messages which they were giving you, do you see like they were giving you enough messages?

MP: Yes, they were giving us enough messages.

Interviewer: Among the messages which they gave you, which ones were difficult for you to understand?

MP: During the first days it was difficult for me to understand the main reason why I was going there. But when I started to learn more from the trainings it was all understood.

Interviewer: Alright. How about your interaction with the expert, how did you see your interaction with the experts?

MP: Our interaction was also good because they are the ones who opened our eyes on antimicrobial resistance, they also told us it’s negative effects, they told us how we can prevent this behaviour to prevent it from spreading. They gave us knowledge in all these.

Interviewer: How were they reacting to your ideas?

MP: They were fine with our ideas and they said they will take all of our ideas and approach other people.

Interviewer: Alright. Now, I want us to talk about the solutions which you designed. What do you think of the approach that you used to design the solutions? How was it?

MP: What was happening with this approach was that they divided us into four groups I think.

Interviewer: Mmh

MP: Among the four groups, every group was coming up with their solutions, and then we were all coming together as one group and discuss the solutions together and then we were prioritizing the solutions. And they were taking those.

Interviewer: Okay. So what do you think of that process?

MP: It is a helpful process, because one person cannot come up with all the ideas for the whole nation. But in this process the solutions were coming from a group

Interviewer: What did you like about this whole process of coming up with the solutions and what didn’t you like which needs to be changed?

MP: I don’t see anything that should be changed because on my own I cannot come with all the right solutions for the whole nation but as a group you make good solutions.

Interviewer: Now, I want us to talk about the co-creation events, how did you see it?

MP: The co-creation event was very good, I’m saying it was good because we chose representatives from all the four groups to make a presentation of their solutions, so it was a beautiful event

Interviewer: How about in terms of the time that you were supposed to be there, the period of time that you spent there on the day or maybe about the venue? What do you think about these things?

MP: When it comes to the venue it was an Excellent venue, the only problem was that I had to walk from the depot to the hotel, but the place was good and everything was fine than the previous venue. But at this venue the food was nice and we were having a cool breeze. Everything was fine.

Interviewer: Alright, how about in terms of time that you were spending there?

MP: There was nothing wrong with the time

Interviewer: what kind of opportunities were you being given to take part in the discussions?

MP: In this process we able to contribute our ideas, we were able to ask questions, which to me I saw that it was good.

Interviewer: Alright. So I also that at the co-creation events you also had other visitors such as the Chiefs, what do you think about that arrangement that some people should be joining you at the end of the co-creation?

MP: It was a very good arrangement, because from what I heard the chiefs were already participating even before us, they were previously involved with the group of the farmers. Apart from the farmers we were joined by other stakeholders as well so it was interesting to hear their inputs on our idea as well.

Interviewer: So now I want us to talk about the solutions that you designed, what do you think about those solutions?

MP: Those solutions were excellent; they were excellent because they were designed in a way that all concerned parties had an input to them.

Interviewer: What do you mean when you say all concerned parties? Who are these concerned parties?

MP: The chiefs, the farmers, of course we didn’t have farmers on the day but their ideas were there. So, we discussed some of these ideas and come up with strong ideas.

Interviewer: How feasible are these solutions?

MP: They can work; I have even been sharing some of the knowledge with patients at the dispensary, for instance a certain patient came to receive amoxicillin at the dispensary but when I checked her medical history I realised that she was already receiving a treatment of another antibiotic, and when I asked her what happened to the other medicines she said she doesn’t like the other drugs because they don’t work on her so she stopped taking them. But I used my knowledge on the dangers of doing that and I advised her to go back home and continue taking her drugs until they are complete.

Interviewer: mmh

MP: So, when we explain to them in details about the dangers of antibiotics people are listening to us.

Interviewer: Alright

MP: Sure

Interviewer: What challenges are you anticipating to come across in trying to implement these solutions?

MP: The challenge that I anticipate is shortage of drugs in the hospital, because for example if we don’t have the drugs and we ask the patients to buy from private pharmacies, and if the patient doesn’t have money he might buy in any quantity depending on his finances

Interviewer: So, we are proceeding

MP: Yes

Interviewer: Now moving forward, from your experience in participating in these events, is there anything that you are doing differently or you are planning to do differently?

MP: There is a huge difference from my previous habits

Interviewer: Alright, what has changed or what will change?

MP: Mostly the change will be reflected on the patients, because we will be sharing with them knowledge about antibiotics which include its effects, the effects of not following the instructions when taking antibiotics, and effects of taking antibiotics without a prescription. We will share all these with the patients

Interviewer: How will that assist us?

MP: It will help in such a way that if we share this knowledge with people we will help to save people from dying due to misuse of drugs and lack of information

Interviewer: Okay

MP: Sure

Interviewer: What challenges do you expect to meet when implementing these solutions?

MP: There is need to have all the required materials to do this job

Interviewer: Okay, what kind of materials?

MP: Materials such as the posters, we need to stick posters around the health facility, we will also need to be conducting health education and we will also need to spread the messages even using radio stations and other activities such as dramas.

Interviewer: Alright, have you shared the knowledge that you acquired on this issue with anyone?

MP: I have share with so many people

Interviewer: Who are these people and how are they reacting to it?

MP: They are clients and they are reacting well to it because I’m explaining to them clearly on misusing of antibiotics.

Interviewer: What kind of worries are they showing or what kind of questions are they asking?

MP: The questions which they are asking mostly is that if you don’t want me to be using antibiotics what other drugs would assist me?

Interviewer: Mmh

MP: So, I tell them that finish the painkillers that have been prescribed to you by the doctor first and if you still don’t feel better then you should go and see the doctor again to examine you.

Interviewer: Alright. We are approaching towards the end of our discussion, now I would like to give you a chance to add or comment anything that you might have forgotten.

MP: I think everything is fine

Interviewer: Alright, thank you very much for your time.

MP: Thank you!
