# Supplementary material for: Co-creating community-driven solutions and policy priorities to address antimicrobial resistance through Responsive Dialogues: A qualitative evaluation from Malawi
Source: PLOS Glob Public Health. 2026 Apr 28;6(4):e0005697. doi: 10.1371/journal.pgph.0005697 (PMC13123971; doi:10.1371/journal.pgph.0005697)
Supplement: S13 Text — (DOCX) [file pgph.0005697.s013.docx]

**Interviewer:** So, firstly let me thank you for giving me the time to participate in this interview.

**FP:** Okay

**Interviewer:** And be open and free, there is no, wrong answers, I just want to hear your ideas. So, firstly I would like to know you, what do you do?

**FP:** Should I start by mentioning my name or?

**Interviewer:** You don’t have to mention it

**FP:** Alright, I work here at [pharmacy] as an administrator, I’m also a sales and dispensing associate.

**Interviewer:** Okay

**FP:** I started working here in 2015 as sales and dispensing associate

**Interviewer:** Okay

**FP:** Later that’s when I started working as an administrator.

**Interviewer:** mmh

**FP:** But I can say I go everywhere since I have got experience, and since I have done this for a longtime. I have got 7years experience

**Interviewer:** So, I want to hear from you. What do you understand when you here about Antimicrobial resistance, AMR?

**FP:** Antimicrobial resistance, right?

**Interviewer:** mmh

**FP:** Its common to me because I’m aware of it, I was told by the pharmacists that I work with here. They tell us the negative effects of misusing antibiotics and the importance of seeking prescription before using antibiotics.

**Interviewer:** Alright. What challenges would antimicrobial resistance cause to animals and humans?

**FP:** The problem that would be caused by antimicrobial resistance is death, because if the person is seek, and the antimicrobials have developed resistance in his body, there he may not recover from the treatment as a result he may die. And it is also the same in animals.

**Interviewer:** It is the same in animals in what way?

**FP:** It is the same in animals because they may also get sick or else if the animals are resistant and if we consume those animals they may also cause resistance in our bodies.

**Interviewer:** Okay. How about in the community, what burdens would it cause?

**FP:** The community will be affected in a way that if the person is sick and he or she is resistant, that patient will be a burden to the community in terms of the care givers

**Interviewer:** What can people do to prevent antimicrobial resistance?

**FP:** They should be taking medications in a recommended way, and they shouldn’t just be taking antibiotics unnecessarily and not any cough requires antibiotics, sometimes you can just take a painkiller.

**Interviewer:** Where did you learn about all this?

**FP:** Like I said I have learnt about this right here

**Interviewer:** You do have these talks?

**FP:** Yes, we do have these talks because this is a pharmacy, so the pharmacist who are qualified with the College of Medicine so they share their knowledge with us.

**Interviewer:** Alright. So, what was your experience in the events which you participated?

**FP:** I had a big experience, for example like I experienced how things are done in some health centers and I also experience how things are done in drugstores. Because we had participants from drugstores, health care workers and pharmacists.

**Interviewer:** mmh

**FP:** They shared some of their observations for instance they said some people go there when they are not even sick, they just go there to receive drugs. They just get the drugs and store them for future use as a result they misuse the drugs as a result they may cause AMR.

**Interviewer:** How about in terms of your commitment for instance the time that you spent there? Or how was the venue?

**FP:** The distance to the venue was long

**Interviewer:** mmh

**FP:** and the environment was fine but the problem was the toilets, they were not good.

**Interviewer:** I think you have started already. Is there anything else that you disliked? Or you would like to change on how things were organized?

**FP:** So far it was good, the only issue for me was the toilets, with the condition I’m in I have to pee severally so to be in a toilet that is uncomfortable it wasn’t alright.

**Interviewer:** Okay. What did you like on how it was organized?

**FP:** What I liked was the way people were committed to make it to the event

**Interviewer:** How did people show that?

**FP:** By showing up in each and every event

**Interviewer:** Alright we are proceeding; how did you find your interaction with the facilitators?

**FP:** The facilitators were very welcoming, as in they were willing to learn and then we should learn from them as well, we could interact and we could chat, and they were very clear when explaining things. One facilitator was handling the recording and other technical issues as well, and the others were assisting us were we didn’t understand, so it was all good.

**Interviewer:** What would you like to change on how it was organized to make it even better?

**FP:** No, it was good, so far to me it was all good.

**Interviewer:** How about your interaction with the experts, how was it?

**FP:** I can say the experts were good, the interaction was the same. It was alright.

**Interviewer:** Alright

**FP:** Only on someday the experts came very late. But so far so good

**Interviewer:** Do you feel like you learnt new things from them?

**FP:** Yes, I did, and they else showed that they were there to learn from us

**Interviewer:** What new things have you learnt from them?

**FP:** They were saying they are doing a research about this, and they all came from various organizations, some of them were coming from [health institution]

**Interviewer:** Okay

**FP:** They were listening to our idea and they were taking note, some of them were policy makers so we expected them to take our ideas into account

**Interviewer:** Alright, is there anything that you would change on your interaction with the experts?

**FP:** No they were okay

**Interviewer:** mmh

**FP:** Most of them were seniors who left their jobs to come and listen to us

**Interviewer:** Alright. Let’s talk about the solutions, how did you find the process of developing the solutions?

**FP:** We had several solutions, for instance we talked about community sensitization through the chiefs and the HSAs because those people work in the communities and they do several talks, we also thought of using flyers and megaphone in a mobile van. So we thought with that we can raise awareness

**Interviewer:** So, how did you find the process of coming up with those solutions?

**FP:** The process was okay. Because we were coming together as a group to discuss it. That’s why we thought there should be awareness, even in health facilities they provide several health talks, they would include this topic to raise awareness to people.

**Interviewer:** Okay, what did you like about the whole process or what did you dislike in this whole process that you used in developing the solutions?

**FP:** I cannot say I disliked, I will be lying if I say that, what I can say is I liked it because those things are important to our health

**Interviewer:** mmh

**FP:** That’s what we face in each and every day. So, it helped us to understand what AMR is, and how we can overcome it.

**Interviewer:** Alright, let’s talk about the co-creation event. The final event that you had

**FP:** mmh

**Interviewer:** How did you find that process or how did it go?

**FP:** According to me I can say I was happy.

**Interviewer:** Okay

**FP:** I can say I was happy because I did the presentation, normally I am not a presenting kind of person but I developed confidence in me that I can do this.

**Interviewer:** mmh

**FP:** And then I was able to represent my group, I was in group 3. So it was interesting and we had the facilitators and we also received other people who came from different organizations and that arrangement gave me hope that these things will be made into a policy and in the next years to come things will be better.

**Interviewer:** Okay

**FP:** Yah something like that

**Interviewer:** How about in terms of time commitment, that you are supposed to be there, or the venue?

**FP:** The venue was okay and in terms of time and being there it was just fine

**Interviewer:** Okay

**FP:** There wasn’t any problem, and going to that event we felt important

**Interviewer:** How was the venue good?

**FP:** It was good because of the environment, the place was very clean and the services were just okay, in fact not just okay but they were good.

**Interviewer:** Okay

**FP:** Yah

**Interviewer:** Is there anything you would change in terms of how the co-creation event was designed?

**FP:** I cannot say I’m against the venue no, but I can just say they should be finding places where people should be comfortable. And for a person to be comfortable there is need to have good food and clean toilets. And the place shouldn’t be noisy, like the venue that we were using before the co-creation event there were some kids in the compound. There was a nursery school

**Interviewer:** mmh

**FP:** Next time they should try to find a hall like the same one but at least there shouldn’t be kids around to avoid the noise.

**Interviewer:** Alright, so you mentioned that there are other groups of people who joined you?

**FP:** Yes, at the co-creation event

**Interviewer:** What do you think about that arrangement that other stakeholders should be joining you at the final event after you have gone through the process?

**FP:** Yah, and some specialists were coming here and there like on the first day we had experts who were with us throughout, so to me it was okay because we wouldn’t accommodate everyone.

**Interviewer:** How about the chiefs, I heard you also had the chiefs on the last day?

**FP:** The chiefs were also eager to learn and they were part of us.

**Interviewer:** So what do you think for them to come on the final day?

**FP:** I would say the chiefs are busy people, they have a lot of duties in the community so we cannot have them all the time. But their presence was very important to us because we felt as one community.

**Interviewer:** Alright, so, I want to talk about the solutions that you developed, or identified, what do you think of its feasibility do you think they are possible to implement?

**FP:** Yes, they are possible they can be implemented however some of the solutions like awareness will need resources like vehicles and people to carry out the work and those people will also need money.

**Interviewer:** Alright, what challenges do you anticipate in trying to implement the solutions?

**FP:** The only challenge will be to change people’s mindset, because if a person has his mindset that his child gets better with amoxicillin, it will be difficult for that person to accept the change at once.

**Interviewer:** Alright, so going forward based on your involvement in the conversation events what have you done differently or what are you planning to do differently?

**FP:** At first I was one of the people who never used to finish the dose of antibiotics, and I was taking antibiotics without any prescription but after participating the conversation events that’s when I learnt that such behavior is a contributing factor to AMR, so now I know that I’m supposed to complete the dose. And when I receive customers I try as much as possible to change their mindset, and we tell them the dangers of using antibiotics but if they don’t listen I just send them to a pharmacist

**Interviewer:** Alright, so who have you spoken to or discuss with regarding this information?

**FP:** I have had discussed this with my parents because they keep on asking me for medicines and when I ask them about the medicines that I gave them previously they keep on telling me that the shared them with friends and I explained to them the negative effects of doing that.

**Interviewer:** mmh

**FP:** And I even discuss with patients

**Interviewer:** Alright, how do they respond or what kind of questions do they ask?

**FP:** Like I said some people have their minds already set so to change them it’s difficult but there are some other people who are able to listen

**Interviewer:** Alright, it looks like we are approaching the end of our interview, do you have anything that you feel like you have missed that you need to explain clearly?

**FP:** No, the only thing I can say is that this problem can be fixed if we follow proper instructions of using drugs

**Interviewer:** Alright. Thank you so much for your time.
