# Supplementary material for: Co-creating community-driven solutions and policy priorities to address antimicrobial resistance through Responsive Dialogues: A qualitative evaluation from Malawi
Source: PLOS Glob Public Health. 2026 Apr 28;6(4):e0005697. doi: 10.1371/journal.pgph.0005697 (PMC13123971; doi:10.1371/journal.pgph.0005697)
Supplement: S14 Text — (DOCX) [file pgph.0005697.s014.docx]

**Interviewer:** Alright, firstly I thank you so much for your acceptance to participate in this interview. Secondly I ask you to feel free there is no right or wrong answer. I just want to hear your views on the various topics that we are going to discuss.

**Respondent:** Alright.

**Interviewer:** So to begin with can I know you, what do you do on your daily basis?

**Respondent:** I work as a builder on a daily basis.

**Interviewer:** You work as a builder as a self-employed or you are employed as a builder?

**Respondent:** I’m employed

**Interviewer:** Alright, you should be raising your voice a bit

**Respondent:** alright

**Interviewer:** So, firstly I would like to know, what do you know about Antimicrobial Resistance?

**Respondent:** At first I didn’t know about Antimicrobial Resistance, but after participating in the conversation events that we had with our colleagues that’s when I gained knowledge that Antimicrobial resistance is dangerous and to use drugs without following proper instructions is a bad habit.

**Interviewer:** Okay

**Respondent:** Yah

**Interviewer:** Can you explain to me what you mean when you say antimicrobial resistance is dangerous?

**Respondent:** What I mean is that when we are sick it means in our body we have certain pathogens, so we buy to drugs to fight against those pathogens, and because sometimes we just buy drugs unnecessarily as a result those pathogens develop resistance to the drugs.

**Interviewer:** Okay

**Respondent:** Sure

**Interviewer:** What are the effects of that to human health or animal health?

**Respondent:** The big effect of this problem is death

**Interviewer:** Alright, how about the community, what consequences would a community face due to antimicrobial resistance?

**Respondent:** As for the community it couldn’t develop, because I said this problem would cause deaths so in the community there couldn’t be development.

**Interviewer:** Alright

**Respondent:** Sure

**Interviewer:** So, how can we prevent antimicrobial resistance?

**Respondent:** we can prevent this problem by not buying drugs carelessly

**Interviewer:** mmh

**Respondent:** And if we are feeling sick we should be going to the hospital and consult a doctor so that the doctor should prescribe the right drugs for us

**Interviewer:** Alright, where did you learn about this?

**Respondent:** I learnt about this when I participated in the conversation event which was organized by Malawi-Liverpool-Wellcome-Trust at [community name]

**Interviewer:** Okay

**Respondent:** Yah

**Interviewer:** Was this your first time to hear about it or you heard about it before?

**Respondent:** Like I said before, this was my first time to learn about this

**Interviewer:** You didn’t know about it previously?

**Respondent:** No

**Interviewer:** Alright, we are moving on. Now, what was your experience in participating in these conversation events?

**Respondent:** At first I had pressure because I didn’t know about it, but during the second meeting I started to learn more about this issue and after everything I had knowledge and I even shared with my friends about it so that they should also learn about it too.

**Interviewer:** So, when you say you had pressure, what part gave you that pressure depending on how the conversations were organized?

**Respondent:** Like I said before I didn’t know what antimicrobial resistance is, so this was like my first time to hear about it so on the first day I had pressure because I didn’t know what this problem is and I didn’t know where to start and what my direction was.

**Interviewer:** Okay

**Respondent:** But on the second meeting my mind was opened and I was open minded throughout the rest of the events.

**Interviewer:** Alright, so you also mentioned about that you shared what you learnt with your friends, was this within the period when you were still in the middle of the conversation events or after you had completed all the events, when exactly did you approach to your friends with this?

**Respondent:** I approached to my friends when we had just begun, the first people that I approached to was my family

**Interviewer:** Okay

**Respondent:** Yes, my family, my parents and people around me, because they were wondering where I have been going to.

**Interviewer:** mmh

**Respondent:** So, that’s when I explained to them what this problem and some people were able to understand

**Interviewer:** What was their reaction to it or maybe what questions were they asking and what was their worry?

**Respondent:** Most of their worry was that they mostly depend on buying drugs from groceries than going to the hospital, and when I explained to them about the importance of going to the hospital they agreed with me and started doing that.

**Interviewer:** Alright. So, we are still discussing about your experience in these meetings.

**Respondent:** Alright

**Interviewer:** Now during these meetings what was your experience in terms of the time that you were supposed to be there or maybe in terms of the duration that you were supposed to spend there and also the place where the events were held?

**Respondent:** In terms of the place and the time it was all good. We had time to have breakfast there, we had time to have lunch, everything was good.

**Interviewer:** Wasn’t it disturbing your work?

**Respondent:** No, I make my own time to work.

**Interviewer:** Alright.

**Respondent:** Sure

**Interviewer:** What did you like and what didn’t you like in terms of your experience in participating in these meetings?

**Respondent:** What I liked the most about it was that everyone was free and open to each other

**Interviewer:** Okay

**Respondent:** And our facilitators were also very free and when they are explaining things to us they were explaining in a way that they were not shouting at us or making us feel uncomfortable but they were explaining in a manner that everyone should understand clearly

**Interviewer:** mmh

**Respondent:** Sure

**Interviewer:** Alright, so how was your interaction with these facilitators?

**Respondent:** When we arrive we were having an opening prayer and then continue with our program, so our interaction was very good.

**Interviewer:** You feel like they were listening to you?

**Respondent:** Yes! They were taking all of our ideas whether it’s right or wrong they were putting everything together, they never excluded any of our ideas but they took every idea in board.

**Interviewer:** Okay

**Respondent:** Sure

**Interviewer:** How about in terms of the messages that they were giving you, do you feel like they were giving you enough messages?

**Respondent:** Yes, they were giving us enough messages and they were also giving us images so that when we don’t understand the message we would refer to the pictures

**Interviewer:** Okay, which message was difficult to understand?

**Respondent:** There wasn’t any message that was difficult to understand but every message that we received I was able to understand it

**Interviewer:** Alright. So we are moving on. Now, I would like to hear about your interaction with the experts, how was your interaction with the experts?

**Respondent:** Our interaction was in a way that we were in a group, so we would seat in a circle or in any seating position of our choice and everyone was asked to feel free and given a chance to speak. So our interaction was good

**Interviewer:** Okay

**Respondent:** Yes, it was all good and I didn’t see any problem

**Interviewer:** They came and they were putting in comments?

**Respondent:** Yes, they came and most of them came during the final event after we the first 3events, they came at a hotel in Town.

**Interviewer:** Okay

**Respondent:** Yah, but it was all good and we had the experts from Lilongwe and other health experts from various places. They all came on that day

**Interviewer:** Okay, do you feel like there were any new things that you learnt from those experts?

**Respondent:** Yes

**Interviewer:** What did you learn from them?

**Respondent:** These experts the clarified further in what we were discussing so we learnt that some of our ideas were indeed strong points from these experts

**Interviewer:** Alright. How were these experts reacting to your ideas?

**Respondent:** they reacted well to our ideas, they never rejected any ideas from a person as an individual or as a group, they were listening to all of our ideas and they even gave us an opportunity to ask questions.

**Interviewer:** Is there anything that you would change in terms of your interaction with these experts?

**Respondent:** There is nothing I would change from it. I was satisfied with how it went

**Interviewer:** Okay. We are moving on. Now, what is your view on the process that you used to come up with the various solutions?

**Respondent:** This process was good to me, at first we had the idea that everyone should present their own ideas as one group but then they told us to be divided into groups and discuss the topics, so if you ask me I was very satisfied with how it went.

**Interviewer:** Okay. What did you like and what didn’t you like about the process that you used to develop these solutions?

**Respondent:** According to me there is nothing that I didn’t like. I liked it all because what we were doing in the groups was being presented to everyone as one group

**Interviewer:** Is there anything that you would change in the process that you used to develop these solutions?

**Respondent:** There is nothing that I would change

**Interviewer:** Alright. Now I would like us to discuss about the final event, the co-creation event that you had, how did that event go?

**Respondent:** That meeting went on well, it took place in town at a hotel

**Interviewer:** Okay

**Respondent:** We agree that everyone should be there at 8:00am, and everyone kept time. The chiefs and other various experts also came.

**Interviewer:** mmh

**Respondent:**  That event went on well and I was satisfied with it

**Interviewer:** What makes you think it went on well or what satisfied you?

**Respondent:** What satisfied me was that after we had discussed everything in the previous events we took everything to a bigger group and discussed everything together, so it satisfied me because it was like the whole team was complete and came together and make one thing

**Interviewer:** Okay. How do you see that arrangement that some people joined you at this final event only for instance the chiefs and other experts came on the final day, what are your views on that particular arrangement?

**Respondent:** At first I asked myself the same question that why are these people joining us at the last event but after I gave it a thought I realized that it was good for them to join us at the last event because these people were professionals and some of the come from far.

**Interviewer:** Alright. How about your views in terms of the time to get there and the duration that you spent there and also the place where the event took place?

**Respondent:** In terms of the time it was all good and the place where the event was held was also good.

**Interviewer:** What makes you say the place was good?

**Respondent:** The place was beautiful, it is well taken care of and everything there was good.

**Interviewer:** Alright. What are your views in terms of being given a chance to contribute in the discussions that took place there?

**Respondent:** In my own point of view everyone was given a chance to speak and if you have a question you were being given a chance to ask and the feedback was good

**Interviewer:** Alright, we are moving own. What do you think about the solutions that you developed?

**Respondent:** On my part I would say if we are to use those solutions and if the government adapts those solutions and use them it would be a very good thing

**Interviewer:** Okay. What challenges do you think we can come across when implementing these solutions?

**Respondent:** The big challenge would be the government, most of the solutions will require government intervention

**Interviewer:** Alright, going onwards after participating in these events, what changes have you made in your daily activities?

**Respondent:** In terms of my daily activities as I said earlier that I work nothing has changed on that but in terms of use the dangers of use of drugs carelessly my life has changed a lot and it also changed my family because we now know the dangers of doing that. But in terms of my daily work activities nothing has changed?

**Interviewer:** Okay. But in terms of antimicrobial resistance, what changes have you made to fight it?

**Respondent:** Okay. There has been a big change, because like I said I didn’t know about antimicrobial resistance, so since I finished those meetings I experienced a big change because some of the activities that I used to do I stopped doing them.

**Interviewer:** What activities are those?

**Respondent:** For instance, I used to buy drugs without seeking prescription from the hospital but now that I have stopped, even when my child is sick I tell my wife not to buy drugs but to take the child to the hospital, so such things have changed in my family.

**Interviewer:** what challenges are you facing to implement these things?

**Respondent:** The challenge is that some people don’t listen but in our community we use the chiefs, so the chiefs announce about these messages during funerals and they ask people to find us for more information, so people find us to ask questions.

**Interviewer:** Alright, this is the end of our discussion but before we close I would like to give you a chance to ask questions if you have any question or if there is anything else that you would like to add?

**Respondent:** No, I don’t have any questions

**Interviewer:** Alright, thank you very much for your time!

**Respondent:** Thank you!
