# Supplementary material for: Co-creating community-driven solutions and policy priorities to address antimicrobial resistance through Responsive Dialogues: A qualitative evaluation from Malawi
Source: PLOS Glob Public Health. 2026 Apr 28;6(4):e0005697. doi: 10.1371/journal.pgph.0005697 (PMC13123971; doi:10.1371/journal.pgph.0005697)
Supplement: S15 Text — (DOCX) [file pgph.0005697.s015.docx]

**Interviewer:** Alright, firstly thank you for your acceptance to take part in this interview, I ask to feel free, there is not right or wrong answer. So, firstly I would like to know that what do you do on your daily basis?

**Respondent:** Okay, I should also thank you for inviting me to take part in this interview. I stay in [community name].

**Interviewer:** Okay

**Respondent:** On my daily basis I work in a chief’s office as his secretary

**Interviewer:** Alright. So to begin with I would like to know what do you know about Antimicrobial Resistance?

**Respondent:** I know few things on that topic

**Interviewer:** mmh

**Respondent:** I learnt few things from the events that we had, at first I never knew what it means when they say antimicrobial resistance, but when I attended the first workshop that’s when I learnt that they are micro-organisms which develop resistance to drugs within the body usually this is caused due to poor hygiene and it is also caused when you buy and take drugs from pharmacies without following a proper prescription from the hospital

**Interviewer:** Okay

**Respondent:** For example, a sick person may go to the hospital to consult with a doctor and then the doctor may prescribe certain drugs for his or her condition and give instructions to the person to take the drugs for one week

**Interviewer:** mmh

**Respondent:** And after taking the drugs for 2days the patient may start to feel better he or she may stop taking the drugs without completing the full dose as advised at the hospital as a result the pathogens may just get weak and not completely be killed as a result the pathogens may regain strength and if the person takes the same drugs to fight against the pathogens it now becomes difficult for him or her to recover because the micro-organisms have now developed resistance.

**Interviewer:** Okay. What are the consequences which result from this problem to human health and animal health?

**Respondent:** The first consequence is that it leads to prolonged sickness

**Interviewer:** Okay

**Respondent:** And it also leads to challenges within his family because the person spends much time seeking treatment

**Interviewer:** mmh

**Respondent:** And in animals it may cause the animal to fail to recover due to resistance as a result the animal may die

**Interviewer:** Okay

**Respondent:** Sure

**Interviewer:** How about in the community, what would be the consequences of this problem to the community?

**Respondent:** If a lot of people have this problem in the community there would be a decline in development and people would have poor health

**Interviewer:** mmh

**Respondent:** And the government would also spend a lot of money to buy alternative drugs

**Interviewer:** Alright. How can we prevent this problem?

**Respondent:** This problem can be prevented by spreading right messages to people for instance informing them to take right drugs, they should be seeking medical treatment from the hospital and if they are given the drugs they should be using the drugs by following the right instructions

**Interviewer:** Okay. Where did you learn about this?

**Respondent:** I learnt about this from the various meetings that I participated in which were organized by Malawi-Liverpool-Wellcome-Trust

**Interviewer:** Was this your first time to hear about it or had heard about it before?

**Respondent:** This was my first time to hear about it

**Interviewer:** Okay

**Respondent:** Because I was also one of the people who never used to go to the hospital when I feel sick, I had a habit of buying drugs from pharmacies instead of going to the hospital but when I went to the first workshop that’s when I realized that I was doing a bad habit.

**Interviewer:** Alright, we are moving on. Now, I want to know what was your experience for participating in these conversation events?

**Respondent:** My experience for participating in these events has benefited me a lot and it has also benefited other people within my community

**Interviewer:** mmh

**Respondent:** Because like I said before I was one of the people who had a bad habit of not going to the hospital when I’m sick, but when I learnt about this problem from the events I started going to the hospital and I also encourage my family to do the same and I also to the message to other people within my community

**Interviewer:** mmh

**Respondent:** I tell them that it is dangerous to take drugs without following the instructions that are given to us at the hospital, and through these messages some people are becoming aware of it

**Interviewer:** Okay, where do you disseminate these messages?

**Respondent:** I disseminate these messages during funeral gatherings, I also disseminate these messages in male chatting joints such as places where we play Bawo and we are also disseminating in churches

**Interviewer:** What is the reaction of people to these messages?

**Respondent:** People are receiving these messages especially from people that know me and some even given comments that what I’m telling them is true, because most people had a habit of storing drugs for future use so that when somebody else gets sick in the house they should share the same drugs.

**Interviewer:** Alright. From your experience what is making it possible to disseminate these messages and what is making it a challenge to disseminate these messages?

**Respondent:** What is making it possible is that we are approaching these people in a group and we share the messages to that group of people

**Interviewer:** mmh

**Respondent:** And what is making I a challenge is that some people don’t listen so as a result they hear about the message but they don’t use it for instance people who are drunk, they may hear about it but never use it.

**Interviewer:** Alright

**Respondent:** Sure

**Interviewer:** Now I want to know how did the time that you used to spend at these events affect your daily basis activities?

**Respondent:** It didn’t affect my daily basis activities because the time that I was spending there was also benefiting me and my family

**Interviewer:** You didn’t have any problem with the time that you were supposed to be there or in terms of the duration that you spent there?

**Respondent:** No, there was no any problem and when we go there they were giving us money and transport refund which was benefiting my family

**Interviewer:** Alright, we are moving on. I want to hear how was your interaction with the facilitators of these events?

**Respondent:** According to me my interaction with these facilitators was very good

**Interviewer:** Okay

**Respondent:** They were teaching us with interest and with professionalism

**Interviewer:** Do you feel like they were giving you enough information in the various topics that you were discussing about?

**Respondent:**  Yes they were giving us enough information because what they were sharing with is indeed what is happening in our communities

**Interviewer:** Alright. Which information was difficult to understand among the information which they gave you?

**Respondent:** Yes, there was a certain information about how other religions which don’t allow their members to take drugs when they are sick and the difficult part was how can we approach that group of people

**Interviewer:** Alright. Is there anything that you would change in terms of your interaction with these facilitators?

**Respondent:** The only thing that can change is that when I’m sick I will have to go to the hospital and my family will also do the same. That is what will change me.

**Interviewer:** Alright, now I would like to know how was your interaction with the experts?

**Respondent:** Our interaction was good because they were teaching and explaining to us about antimicrobial resistance and within their explanation they also told us that there are several types of antibiotics and it’s not just only one drug for instance they told us that there is Bactrim, Amoxicillin, penicillin and many more and they were telling us that these drugs are prescribed by doctors and after a proper diagnosis on the patient

**Interviewer:** Alright. How were these experts responding to your ideas?

**Respondent:** They were responding to our ideas properly and there were giving us good insights

**Interviewer:** Alright, now I want to know how do you think about the process which you used to come up with the various solution which you came up with?

**Respondent:** According to me it was a very good process because through that process we were able to identify solution which would benefit Malawi as a country but also worldwide. So, developed solutions that we feel like if we are to make these solutions as policies they will benefit the whole country and the whole world.

**Interviewer:** Okay. What did you like and what didn’t you like in terms of how the process was organized?

**Respondent:** According to me I liked the process a lot and there is nothing that I didn’t like about the whole process

**Interviewer:** What did you like about it?

**Respondent:** What I liked the most is the topics that we were learning about because we learnt about things which will benefit our health

**Interviewer:** Alright. So, I want us to discuss about the co-creation event, what are your views on that event?

**Respondent:** The final event was an outstanding event because it was a final event and it brought various people together whereby we shared ideas and co-created ideas which we thought would be helpful to the whole country

**Interviewer:** Okay, what do you think could be the challenges that you would face in implementing those solutions that you discussed?

**Respondent:** The big challenge that I think could make it difficult to implement is funds, for these things to be implemented they will require enough funds.

**Interviewer:** Alright, Now I want to hear your views on what you think about the time that you were present there and the duration on that day at the event, how did you see it?

**Respondent:** The time was very good and when they tell us that we are done for that particular day we were even surprised that that the time has gone so fast because everything was interesting

**Interviewer:** Okay, how about in terms you being given a chance to participate in that event how did you see it?

**Respondent:** It made me an open minded person and I am able to share what I learnt from there with other people

**Interviewer:** Alright, so you mentioned that there are other groups which joined you at the final event, what do you think about that arrangement that some other people should be joining you at the final event only?

**Respondent:** It was good that those other people joined us at the end, because most of them were already experts who helped us to create solid solutions

**Interviewer:** Alright, moving forward, what have you changed or what are you planning to do differently on your daily living?

**Respondent:** My daily activities have changed because when I’m leaving the house I make sure that I check if there is anyone that is sick in the house and I also make sure to maintain good hygiene at the house, I have also share messages with other people so that they should also benefit

**Interviewer:** What is the importance of that hygiene and the sharing of messages with other people?

**Respondent:** The importance of the hygiene is that it will help to prevent other diseases which are caused by bacteria due to lack of hygiene

**Interviewer:** Okay. What can make this hygiene to be feasible and what can make it difficult to implement?

**Respondent:** This can be made possible by using village committee groups which would teach people in the village on the importance of hygiene on daily basis

**Interviewer:** Okay, what are the challenges can this solution come across?

**Respondent:** The challenge that this solution can come across is that the houses in our communities are too close to each other as a result it would be very difficult to maintain hygiene, however government provide us with chlorine to use in stagnant water.

**Interviewer:** Alright. This is the end of our discussion, but before we close I would like to give you an opportunity to say anything that you feel like you left it out?

**Respondent:** My final words that I would like to say that the problem of antimicrobial resistance is very dangerous and I would like to ask the health care workers to be clear when giving us the antibiotics and they should be mentioning to people that we are giving you antibiotics and you will have to complete the full dose. Because some people don’t complete the dose because they are not told that they are supposed to complete the dose. That’s all I wanted to add.

**Interviewer:** Alright. Thank you very much for your time!

**Respondent:** Thank you!
