# Supplementary material for: Co-creating community-driven solutions and policy priorities to address antimicrobial resistance through Responsive Dialogues: A qualitative evaluation from Malawi
Source: PLOS Glob Public Health. 2026 Apr 28;6(4):e0005697. doi: 10.1371/journal.pgph.0005697 (PMC13123971; doi:10.1371/journal.pgph.0005697)
Supplement: S16 Text — (DOCX) [file pgph.0005697.s016.docx]

**Interviewer:** Alright, so we are starting.

**Respondent:** Alright

**Interviewer:** Firstly, I ask you to feel free, there is no right or wrong answer, mostly I just want to hear about your views.

**Respondent:** Alright

**Interviewer:** So to begin with, I would like to know what do you do on your daily basis?

**Respondent:** On my daily basis I run my business

**Interviewer:** mmh

**Respondent:** And I’m also the chief’s counsel in [community name].

**Interviewer:** Alright

**Respondent:** Sure

**Interviewer:** So now, I would like to know what do you know about the issue of Antimicrobial Resistance?

**Respondent:** From the training that we had it opened my mind on this issue of antimicrobial resistance due to taking of drugs without following its instructions

**Interviewer:** What causes this issue?

**Respondent:** This is caused by us people. For example, sometimes us men we may receive drugs from the hospital and when we start to feel better we stop taking the drugs without completing the full dose and we share the remaining drugs with our colleagues yet we didn’t finish the dose.

**Interviewer:** mmh

**Respondent:** So that habit doesn’t kill the microbes in our bodies but it just causes the microbes to faint as a result when we try to take drugs again they fail to work in our bodies

**Interviewer:** Okay

**Respondent:** Sure

**Interviewer:** What problems does this cause to human health or animal health?

**Respondent:** This issue puts our lives in danger, because if we get sick again and get prescribed the same drug at the hospital, the drug may not work anymore in our body

**Interviewer:** Okay. How about in terms of the community, how would it be affected with this issue of antimicrobial resistance?

**Respondent:** It’s just like with Cholera outbreak or cough, if we don’t follow good hygiene habits it’s easy for the disease to spread in the community it is also the same with this issue if we don’t follow the instructions it is easy for this problem to spread in the community

**Interviewer:** Alright. So how can we prevent this problem?

**Respondent:** My main point will still be focused on the drugs, when we get sick we should be seeking treatment from the public hospital and we should make sure to complete the full dose of the drugs that has been prescribed to us by the doctor. If we follow the instructions, then we will be able to deal with this problem.

**Interviewer:** Alright. We are moving on.

**Respondent:** sure

**Interviewer:** Now what I would like to here is what was your experience in taking part in these conversation events?

**Respondent:** Taking part in these events benefited me a lot as an individual and it also benefited my family and the whole community, because I realized the dangers of not seeking prescription from the hospital and the dangers of sharing drugs without completing the dosage.

**Interviewer:** Okay

**Respondent:** These points benefited us a lot and when we share these points with people during funerals they seem to agree with us and due to that training a lot of people are benefiting from it.

**Interviewer:** Alright, how about in terms of your time that you spent there or distance or maybe the duration or in terms of the venue where these events were taking place, what is your view on that?

**Respondent:** In terms of the distance to the venue there was no any problem, the time was also fine because we were meeting at a good time. The venue was also alright. That’s what I would say in short.

**Interviewer:** Okay. Where did you learn about antimicrobial resistance, was it at these events or you already knew about it from the past?

**Respondent:** To say the truth it was my first time to hear about antimicrobial resistance and I heard it from Malawi Liverpool Wellcome Trust

**Interviewer:** Alright. Now, in terms of how these meetings were organized and in terms of your experience in participating in these meetings, what did you like and what didn’t you like about it?

**Respondent:** These events were organized well from the beginning up to the end and all the messages that we were sharing there were to benefit our health. So, it was good.

**Interviewer:** Okay. There is nothing that you would change about how these meetings were organized?

**Respondent:** The only thing that I was expecting to change was the allowance, because we changed the venue on the last events to a higher quality venue so I was expecting that the allowance will also change. This was the expectation of a lot of us that participated. But we were not disappointed.

**Interviewer:** Alright, we are moving on. Now, I would like to know how was your interaction with the facilitators of these meetings?

**Respondent:** To say the truth these people are well trained people, they were very interactive, they were not selfish, I would say the interaction with them was 100% good.

**Interviewer:** Okay, do you feel like they were listening to you?

**Respondent:** They were listening to us and they were giving us a chance to ask questions and they were responding to all of our questions and they were telling us to feel free and they were open to us.

**Interviewer:** Alright

**Respondent:** Sure

**Interviewer:** How clear were the messages that these facilitators were giving you?

**Respondent:** The messages that they were giving us was very clear hence I’m still able to answer some of the questions that you are asking me and to say the truth those people are very intelligent because everything that they taught us we still remember up to now and we still spread the messages in our community gatherings.

**Interviewer:** Okay.

**Respondent:** Sure

**Interviewer:** Maybe which message was difficult to understand from the various messages that you received?

**Respondent:** The only difficult part was to understand the problem tree analysis but we all understood the assignment when the facilitators explained it and we all came up with similar solutions.

**Interviewer:** Alright. Now, how was your interaction with the experts on this issue of antimicrobial resistance?

**Respondent:** Our interaction was good because they were not taking themselves as superior but they were putting themselves on the same level like us. They were just correcting us when we are sharing what we have discussed but there wasn’t any problem with them in short.

**Interviewer:** What new thing did you learn from the experts?

**Respondent:** What I learnt from them is that when you are trying to learn you need to be humble and respectful because that’s how they were and that’s also how the facilitators treated us

**Interviewer:** Alright. We are moving on. Now, I would like us to talk about the various solutions that you developed, what are your views on those solutions in terms of dealing with antimicrobial resistance?

**Respondent:** I will come back to the point that I mentioned about the problem tree analysis, when we finish drawing the tree we were coming up with the solutions, some of the solutions that we came up with were in a manner that we should be able to implement on our own when we return to our communities and that we should be able to share with our colleagues in several community gatherings such as in churches, funerals and in chatting joints such as at bawo.

**Interviewer:** Alright. What are your views on the procedure that you used to design these solutions?

**Respondent:** The procedure was very good because we were divided into groups and then all our groups were coming together and discuss the solutions and we would come up with one strong solution and the facilitators were also learners during this time because they were participating the same way like us.

**Interviewer:** What did you like about this procedure and what didn’t you like about this procedure?

**Respondent:** What I liked the most is the idea of dividing us into groups so that everyone should come up with their own ideas and then bringing all those ideas together so that we should take it out to the nation.

**Interviewer:** Okay

**Respondent:** Sure

**Interviewer:** Now, in terms of feasibility of these solutions, for instance you mentioned of sharing the messages, do you think these solutions would help to deal with antimicrobial resistance? Or how feasible are these?

**Respondent:** The solutions that we came up have higher feasibility however they have some challenges for instance we identified that there are some people who don’t visit the hospital, and some don’t take medications due to their religious beliefs, so to deal with such challenges we agreed that some of the solutions should be given to our policy makers such as members of parliament so that they should make them mandatory.

**Interviewer:** Alright. Now, I would like us to talk about the final meeting that took place the co-creation event, what are your views on this event?

**Respondent:** The co-creation event was an outstanding event, our facilitators took us to an outstanding venue, they provided us with internet to everyone that was using internet, we were given freedom and we shared what we like, so I would say our facilitators were well prepared for this event, there was no any problem with that event.

**Interviewer:** Alright. Didn’t it consume your personal time to participate at that event? And also the duration of that particular day, what are your views on these issues?

**Respondent:** Considering that I’m a leader, what we were learning there was very important and it was benefiting our health, so there was no any problem with the time and in fact we all thought we needed more time. And there was no any issue with the distance.

**Interviewer:** Alright. Now, how about in terms of being given a chance to participate in these events?

**Respondent:** This was very good, we were being given a chance to participate and at the end of the meeting one of us would get a chance to say closing remarks and everything was good and during the final event I was the one who said the closing remarks.

**Interviewer:** Alright.

**Respondent:** Sure

**Interviewer:** Now, I want to ask you that some of the people such as the chiefs joined you at the final meetings, what do you think of that arrangement, was it good that they should be joining you at the end or they should have joined you earlier?

**Respondent:** Thank you for that question, I think our facilitators they thought well in this arrangement because you cannot put a standard one student together with a standard eight students, I’m saying this because we were the ones that were completely illiterate but the chiefs were already previously involved in these meetings so it was a good arrangement and some people would haven’t felt free to speak in front of their leaders due to fear.

**Interviewer:** Alright, we are moving on. Now, I would like to know what have you changed on how you do your daily activities or what are you planning to do differently since participated in those meetings?

**Respondent:** I have seen a lot changes in me since I attended those meetings, I’m now able to warn people on the dangers that may result due to misuse of drugs and the dangers of sharing drugs without prescription and we share these messages in our community gatherings

**Interviewer:** So how are people reacting to it when you are sharing these messages with them?

**Respondent:** I remember there was a funeral in the community and when they gave the chief time to speak to the people he talked about the funeral and at the end of his speech he added the message about antimicrobial resistance telling people the dangers of not completing the full dose of antibiotics that such habits cause antimicrobial resistance, people craped their hands after receiving this messages and they showed a lot interest. I’m telling you this from what I know because it really happened when I was there.

**Interviewer:** Okay.

**Respondent:** Sure

**Interviewer:** Alright, thank you very much for this interview, this is the end of our interview.

**Respondent:** Thank you!
