# Supplementary material for: Co-creating community-driven solutions and policy priorities to address antimicrobial resistance through Responsive Dialogues: A qualitative evaluation from Malawi
Source: PLOS Glob Public Health. 2026 Apr 28;6(4):e0005697. doi: 10.1371/journal.pgph.0005697 (PMC13123971; doi:10.1371/journal.pgph.0005697)
Supplement: S17 Text — (DOCX) [file pgph.0005697.s017.docx]

**Interviewer:** Alright we are starting.

**Respondent:** mmh

**Interviewer:** Firstly, thank you for accepting to take part in this interview

**Respondent:** mmh

**Interviewer:** So, first of all I would like to know, what do you do on your daily basis?

**Respondent:** Well on a daily basis I’m employed, I also do house chores and apart from that I am also a church elder.

**Interviewer:** Alright

**Respondent:** And I’m also a chief’s assistant

**Interviewer:** Alright, now I want to know, what do you know about antimicrobial resistance?

**Respondent:** I know something about it because in the previous year’s according to my age, when we get sick and get medicine from the hospital, we were recovering

**Interviewer:** mmh

**Respondent:** But during these current days when you get sick and take the drugs you find that the body isn’t recovering

**Interviewer:** Okay

**Respondent:** And sometimes you go back to the doctor and tell him that you are not seeing any recovery change after taking the drugs

**Interviewer:** Alright, what is the cause of that problem?

**Respondent:** What was happening in the previous year is that, like I said with my age experience, what was happening in the previous years was that when we start to feel better after starting the drugs we were not completing the dose

**Interviewer:** okay

**Respondent:** we used to think that the doctor has given us a lot of drugs than necessary as a result we were storing the remaining drugs from the dosage

**Interviewer:** What effects does that cause to humans or animals?

**Respondent:** That behavior was affecting us in such a way that when we don’t complete the dosage after a short time we were starting to get sick again and that is because the first dose which you didn’t finish it didn’t completely kill the microbes but it just made them weak hence why you start get sick again.

**Interviewer:** Alright, how about as a community, how would it be affected by antimicrobial resistance?

**Respondent:** People in the community if they are not completing the full dose they would end up having prolonged sicknesses because the microbes will develop resistance in their bodies

**Interviewer:** Okay, so how can prevent this issue of antimicrobial resistance?

**Respondent:** This issue of not completing drugs we were doing it due to lack of awareness that such behavior is bad, so the solution to this problem is to raise awareness on the dangers of misusing antibiotics

**Interviewer:** Where did you learn about this?

**Respondent:** I learnt this from the workshops which I was invited to

**Interviewer:** You have never heard about it previously?

**Respondent:** I never heard about it previously

**Interviewer:** Alright. Now I would like us to discuss about your experience in taking part in those meetings, for instance your experience on the time that you were supposed to meet there or may in terms of the venue, what are your experiences on those issues?

**Respondent:** According to me I didn’t feel like I wasted time in attending those meeting because by attending those meeting I acquired knowledge that when you misuse drugs the microbes in your body may develop resistance

**Interviewer:** Okay, how about in terms of the venue where you were meeting at, how accessible was the venue or what are your views on the venue?

**Respondent:**  About the venue I would say it was easy to get to the venue but the only problem that I encountered at the first venue was the issue to do with the toilets, the toilets were really bad.

**Interviewer:** Was there any other problem that you encountered at the venue?

**Respondent:** It was just the toilets, they were bad

**Interviewer:** Alright, what would you like to change on how things were in your experience?

**Respondent:** The only change that I would do is that I have now learned that I’m supposed to complete the full dose and follow the instructions that have been given to me by the doctor for me to have a full recovery

**Interviewer:** Alright, so I would like us now to talk about the various meetings that you had. Firstly, I want to know how was your interaction with the facilitators?

**Respondent:** In all these meetings that I have attended these facilitators facilitated very well but the only problem that I encountered was time, we were not having enough time. I’m saying this because we were having several topics to discuss so sometimes due to lack of time we were being rushed to finish one topic so that we should start another topic to avoid stealing time of another topic

**Interviewer:** Okay. What message was difficult to understand from the various messages that the facilitators gave you?

**Respondent:** The message that was difficult to understand was the issue to do with administering human drugs to animals, some people administer human drugs to animals so that was something that gave me a lots of thinking that can human drugs work in animals too.

**Interviewer:** mmh

**Respondent:** But after the discussions that’s when realized that such behavior is not acceptable

**Interviewer:** Okay, we are moving on. Now, I would like to know about your interaction with the experts on this issue of antimicrobial resistance, I believe that you had experts right?

**Respondent:** Yes

**Interviewer:** How was your interaction with these experts?

**Respondent:** Our interaction was very good and especially on these issues of antibiotics they really went deep on it.

**Interviewer:** And how were they reacting to your ideas?

**Respondent:** The only challenge that we had was about differentiating between antibiotics and painkillers. This was a problem with us because we were depending more on drugstores and markets were people who sell us drugs in those places sometimes don’t even have knowledge of those drugs.

**Interviewer:** Alright, how were these experts reacting to your ideas? Were they listening to your idea? Or they were just giving you insights?

**Respondent:** They were giving us insights on how the antibiotics work and how painkillers work.

**Interviewer:** Alright, now I would like us to discuss about the process that you used to develop the solutions, what do you think about the process that you used?

**Respondent:** The process that we used was good because the facilitators divided us into groups and each group was given a topic to discuss on then in the groups we were coming up with the root causes and then we were developing solutions to those problems. Then after we finish discussing in the groups each group was choosing a representative to present what was discussed in their group to the rest of the groups.

**Interviewer:** From how you have explained about the process that you used, what did you like about this process and what didn’t you like about it?

**Respondent:** Firstly, I liked about the idea of putting us in the groups through that process everyone was being given a chance to contribute their ideas

**Interviewer:** Okay

**Respondent:** Even if we had ten ideas, each idea was being taken and as a group we were discussing all those ideas to come up with one solid idea

**Interviewer:** Okay

**Respondent:** So that’s what I loved the most because the points were coming from a group

**Interviewer:** What didn’t you like about it?

**Respondent:** What I didn’t like was that I learned that some doctors don’t spend much time to listen to the patient and also some doctors they spend much time chatting instead of treating the patients which results into causing long queues

**Interviewer:** Alright, now I would like to know in your opinion, how feasible are the solutions that you developed? Do you think they would to deal with this problem?

**Respondent:** The main cause of this problem was lack of knowledge on the proper use of antibiotics so there is need for government to civic educate people using ministry of health and community leaders.

**Interviewer:** What would be the challenge to implement that civic education?

**Respondent:** The main challenge is that people hesitate when it comes to health issues, some people are even lazy to go to the hospital, they visit the hospital when the sickness gets worse. So people would neglect this.

**Interviewer:** Alright who else have you shared these messages with in your community?

**Respondent:** Me personally?

**Interviewer:** Yes

**Respondent:** I have shared what we discussed with other people in my community, I tell them to visit the hospital when they feel sick

**Interviewer:** Which groups of people are you sharing these messages with?

**Respondent:** As the chief’s messenger I share with all groups of people in community gatherings, for instance we have an upcoming community gathering^th^where we share these messages after we are done.

**Interviewer:** How were people responding to the messages when you were sharing with them?

**Respondent:** People responded very well because some of the issues are really happening because for instance some people dead for not going to the hospitals due to religious beliefs and people were aware of such incidents in the community.

**Interviewer:** Alright, this is the end of our interview, I thank you so much for your time.

**Respondent:** Thank you!
