# Supplementary material for: Co-creating community-driven solutions and policy priorities to address antimicrobial resistance through Responsive Dialogues: A qualitative evaluation from Malawi
Source: PLOS Glob Public Health. 2026 Apr 28;6(4):e0005697. doi: 10.1371/journal.pgph.0005697 (PMC13123971; doi:10.1371/journal.pgph.0005697)
Supplement: S18 Text — (DOCX) [file pgph.0005697.s018.docx]

**Interviewer:** Firstly, I thank you for accepting to take part in this interview, I ask you to feel free, there is no right or wrong answer.

**Respondent:** mmh

**Interviewer:** To begin with I would like to know you, what do you do on your daily basis?

**Respondent:** Thank you, I stay here in [community name]

**Interviewer:** Okay

**Respondent:** To earn a living I work as a tailor, I’m self-employed I have my own shop.

**Interviewer:** Okay

**Respondent:** Sure

**Interviewer:** Alright. Now I want to know what do you know about the issue of Antimicrobial Resistance?

**Respondent:** I know about this issue because I have been told about it, what happen with this issue is that when you are taking drugs and stop taking them once you start to feel better without completing the full dosage it makes the micro-organisms in the body to get used to the drug and when the micro-organism gets used to the drug even when you suffer from the same disease when you take the same drug it doesn’t work anymore.

**Interviewer:** Okay

**Respondent:** Sure

**Interviewer:** What problems does that cause to human health or animal health?

**Respondent:** The problem that this cause to human health is that we only have few of these drugs as a result the drug stops working in the body because the micro-organisms have now developed resistance

**Interviewer:** Alright

**Respondent:** Sure

**Interviewer:** How about to the community, how does this issue affect the community?

**Respondent:** In the community it means they will be a lot of diseases

**Interviewer:** mmh

**Respondent:** Secondly, there will be no development in that community because of the prolonged sickness due to resistance to drugs

**Interviewer:** Okay

**Respondent:** And it also gets affected economically

**Interviewer:** Alright, how can we prevent this issue of antimicrobial resistance?

**Respondent:** We can prevent this by following the instructions that are provided by the doctor, we can also prevent this by not buying drugs carelessly. You need to consult a doctor if you are sick and follow the instructions that are given to you by the doctor

**Interviewer:** Alright

**Respondent:** Sure

**Interviewer:** Where did you learn about this?

**Respondent:** I learnt about this from the conversation events that were organized for us to learn about this topic

**Interviewer:** Okay

**Respondent:** That was the first time that I got to know about this issue.

**Interviewer:** This was your first time to hear about it?

**Respondent:** Yes! it was my first time to hear about it, previously we were only hearing about TB in the radios.

**Interviewer:** Alright

**Respondent:** Sure

**Interviewer:** So, these conversation events that you mentioned, what was your experience in participating in this events?

**Respondent:** To me it was very beneficial, and all of us when we get out of the conversation events we were appreciating that this is indeed a big issue and maybe these events should have started long time ago.

**Interviewer:** When you say they were beneficial to you; in what way did you benefit from it?

**Respondent:** In a way that my mind was opened in anything relating to this issue

**Interviewer:** Alright

**Respondent:** Sure

**Interviewer:** How about in terms of how these meetings were organized maybe the time that you were supposed to be there and duration, how did you see it?

**Respondent:** Everything was good because the meetings were interesting

**Interviewer:** How about in terms of the venue?

**Respondent:** The venue was all good, because venue isn’t an issue but what we were discussing there.

**Interviewer:** Maybe in terms of the direction to get to the venue?

**Respondent:** It wasn’t a problem the directions were simple

**Interviewer:** What would you like to change on how the meetings were organized?

**Respondent:** What can change is that as I’m out here I’m also gathering some more information so that when we are meeting again I should be able to contribute what I have also find out in the community

**Interviewer:** Alright, according to what you have said, is there anything new that you have found out in the community?

**Respondent:** The new thing that I have identified is that there is need to communicate with community members and health workers so that they should work together.

**Interviewer:** Alright, now I would like to know, how was your interaction with the facilitators of these meetings?

**Respondent:** To say the truth we were listening to each other, it didn’t seem like anyone knew anything we were all asking ideas from each other

**Interviewer:** Okay

**Respondent:** We were all one

**Interviewer:** Okay

**Respondent:** Sure

**Interviewer:** Were they giving you enough information?

**Respondent:** The information was enough and up to this day in my head I have enough information about this issue

**Interviewer:** Was there any information that was difficult to understand?

**Respondent:** At the beginning the information that was difficult was the word of “Antibiotics” because that word is in English and we were asking ourselves that what if we come up with our own Chichewa name for this antibiotic word.

**Interviewer:** Alright. We are moving on

**Respondent:** Sure

**Interviewer:** What was your interaction with the experts?

**Respondent:** Our interaction was also the same, they were giving us a chance to explain what we know and when we are mistaken something they were jumping in to correct us and when we correct it they were also agreeing with us showing that we are moving together.

**Interviewer:** Alright

**Respondent:** Sure

**Interviewer:** What did you learn from these experts?

**Respondent:** These experts mostly encouraged us that we should share the messages that we have acquired at the meetings with our friends in the communities

**Interviewer:** Okay

**Respondent:** So to say the truth some people now know about this issue through those meetings

**Interviewer:** How were they responding to your ideas?

**Respondent:** Like I said before they were respecting our ideas and we were all one

(Paused)

**Interviewer:** Alright, we can continue?

**Respondent:** Yes

**Interviewer:** Alright, now I want to know how was the process that you used to develop the various solutions that you developed?

**Respondent:** What was happening was that we were divided into groups and each group was given a topic to discuss, and in each group we were having a facilitator and all together we were coming up with solutions

**Interviewer:** what are your views on that process?

**Respondent:** That process was a very good process to me because we were identifying what the problem is and then we were finding the solutions to cut the root causes of that problem

**Interviewer:** Alright

**Respondent:** Sure

**Interviewer:** What did you like and what didn’t you like about that process of developing the solutions?

**Respondent:** There is nothing that I didn’t like, everything about it was good

**Interviewer:** Alright, now I would like us to discuss about the final event, the co-creation event, what are you views on that event?

**Respondent:** That event to me was good to me and my only problem was the time, the time was short because the discussions were very interesting

**Interviewer:** How about I terms of the venue?

**Respondent:** The venue was very good

**Interviewer:** It was good in what way?

**Respondent:** In a way they received us and our seating plan, everything was good.

**Interviewer:** What are your views in terms of being given a chance to participate in those discussions?

**Respondent:** From my point of view the visitors were mostly there to listen to what we have come up with, we were the ones that had most of the opportunities to speak

**Interviewer:** Okay

**Respondent:** Sure

**Interviewer:** What would you change or do differently on how the co-creation event was organized?

**Respondent:** According to me I think my only problem was with the time, I wish we had more time

**Interviewer:** Alright

**Respondent:** Sure

**Interviewer:** What are your views on the arrangement that some people joined you during the co-creation event only when you had already completed the other first events for instance the chiefs, how do you see that arrangement?

**Respondent:** To me that was a good arrangement, because those people were like our visitors to ask us questions on what we had come up with. So it was a good arrangement because they were able to ask us questions on the solutions that we had developed

**Interviewer:** Alright

**Respondent:** Sure

**Interviewer:** What do you think about the solutions that you developed?

**Respondent:** In my point view my only hope is that wherever those solutions will be taken to for further prioritization they should take a good look at all the solutions

**Interviewer:** You feel like there should be an overview on the solutions from the way you developed them?

**Respondent:** They should be overlooked by other groups of people

**Interviewer:** Which group of people?

**Respondent:** These issues concerning medications involve policy makers, so those policy makers are the ones who need to overlook at these solutions and make a priority

**Interviewer:** Alright. How feasible are these solutions in dealing with antimicrobial resistance?

**Respondent:** These solutions would be possible if we all work together starting from the hospital and the patients that are receiving the drugs. In so doing the upcoming generation will be more aware of this issue because of the messages.

**Interviewer:** Okay

**Respondent:** Because to say the truth this issue of resistance is a new thing here in Malawi, we are just realizing it now.

**Interviewer:** What Challenges do you think you these solutions would come across?

**Respondent:** The first challenge is lack of funds, because for everything to work there will be need for funds

**Interviewer:** Alright. So moving forward from your participation in these meeting what have you changed in how you do things or what are you planning to do differently?

**Respondent:** To me what I have been doing since attending those meetings is that I have been sharing messages on the dangers of misusing the drugs, sometimes I go to the community clinic and have a chat with people, and I look at the drugs which they have received and if it’s a painkiller I let them go but when it’s an antibiotic I tell them to complete the full dosage even if they start feeling better.

**Interviewer:** Okay

**Respondent:** So that’s what I’m doing differently

**Interviewer:** Okay

**Respondent:** Sure

**Interviewer:** How important is it to share such messages?

**Respondent:** It is important because it will help other people who never attended those meetings to gain knowledge about this issue

**Interviewer:** What challenges are you facing in sharing these messages or what challenges are you expecting to come across?

**Respondent:** The challenges that are there is that some people require deep understanding of this issue so for us we cannot go much deeper in this issue because we don’t have the expertise in this issue

**Interviewer:** Okay, which group of people have you shared these messages with?

**Respondent:** I have share these messages in community meetings and I have also shared these messages in funerals. And people ask me questions and when I cannot answer I ask them to go and ask the doctor.

**Interviewer:** Alright. This is the end of our discussion, I thank you so much for your time

**Respondent:** Thank you, I wish we had more time

(Laughter)
