# Supplementary material for: Co-creating community-driven solutions and policy priorities to address antimicrobial resistance through Responsive Dialogues: A qualitative evaluation from Malawi
Source: PLOS Glob Public Health. 2026 Apr 28;6(4):e0005697. doi: 10.1371/journal.pgph.0005697 (PMC13123971; doi:10.1371/journal.pgph.0005697)
Supplement: Attachment — Submitted filename: Response to Reviewers.docx [file pgph.0005697.s019.docx]

**Response to Reviewers**

Manuscript ID: PGPH-D-25-03764

*Co-creation of community-driven solutions and policy priorities to address antimicrobial resistance through Responsive Dialogues: A qualitative evaluation from Malawi*

PLOS Global Public Health

Dear Dr. Bipin Adhikari and Reviewers,

Thank you for the constructive and insightful feedback on our manuscript. We are grateful for the time and effort invested in reviewing our work. We have carefully considered each comment and have made substantial revisions to strengthen the manuscript. Below, we provide a detailed point-by-point response to each comment. Our responses are presented in blue text, and all changes are visible as tracked changes in the revised manuscript.

# Academic Editor Comments

**Editor Comment 1 – Title:** *Consider modifying title to ensure it aligns with the most used terminologies. Suggested: “Co-creation of community-driven solutions and policy priorities to address antimicrobial resistance through Responsive Dialogues: A qualitative evaluation from Malawi.”*

**Response:** We have adopted the recommended title: “Co-creation of community-driven solutions and policy priorities to address antimicrobial resistance through Responsive Dialogues: A qualitative evaluation from Malawi.” The change from “policy asks” to “policy priorities” better aligns with standard terminology in the field. We also added a hyphen to “community-driven” for grammatical consistency.

**Editor Comment 2 – Introduction: Multi-sectoral AMR contributions:** *Expand/acknowledge the multi-sectoral/multi-factorial contributions to AMR and why so more prominently in LMICs (e.g. regulatory, health system, formal/informal health services). Reference: Pokharel et al. (2019) BMJ Glob Health, PMID: 31799007.*

**Response:** We have added a new paragraph after the existing text on structural health system challenges in sub-Saharan Africa. This paragraph addresses: weak regulatory frameworks and poor enforcement of antibiotic sales policies; the role of informal health services (drug shops, drug dispensers, traditional practitioners) as the primary point of care where formal healthcare is constrained; economic incentives driving inappropriate antibiotic use on both the patient and seller sides; and antibiotic use in the agricultural sector by farmers lacking access to veterinary services. The paragraph concludes by underscoring the need for multi-sectoral, participatory approaches across the One Health spectrum. We have cited the suggested reference, Pokharel et al. (2019), along with existing references on antibiotic use patterns in Malawi (Mithi et al., 2024; MacPherson et al., 2022).

**Editor Comment 3 – Methods: Supplementary files:** *Could you share semi-structured interview guide and FGD guide as supplementary files?*

**Response:** We have included the semi-structured interview guide and focus group discussion guide as S1 File and S2 File, respectively. A reference to these supplementary files has been added in the Methods section.

**Editor Comment 4 – Methods, Line 130: Rationale for cattle and poultry farmers:** *Add a line why you interviewed cattle and poultry farmers among animal sector?*

**Response:** We have added the following justification after “livestock farmers (cattle and poultry)” in the Participants section: “Cattle and poultry farmers were included because livestock farming in Malawi involves substantial antibiotic use for disease prevention and growth promotion, representing a critical stakeholder group within the One Health framework.”

**Editor Comment 5 – Discussion: Poverty as structural barrier:** *Expand on poverty as a significant barrier; these are structural issues, quite systemic, and informal healthcare/services act as patches for structural deficit. References: Adhikari et al. (2021) PMID: 33975888; Adhikari et al. (2024) PMID: 38569658.*

**Response:** We have substantially expanded the discussion of poverty as a barrier. The revised text now discusses how informal health services, including drug shops and over-the-counter antibiotic dispensers, function as patches for structural deficits in formal healthcare systems (citing Pokharel et al., 2019; Adhikari et al., 2021). We address the ethical tensions identified by Adhikari et al. (2024): between personal economic interests of individuals whose livelihoods depend on antibiotic sales, and the collective interest in curbing AMR; and between addressing immediate health needs and preserving antibiotic effectiveness for future generations. We conclude that participatory approaches like RDs cannot alone resolve these structural contradictions without concurrent investment in strengthening health systems, improving access to formal healthcare, and providing alternative livelihood options.

**Editor Comment 6 – Discussion, Line 402: Desire for feedback:** *Not clear. Explain why there was a desire for feedback and continued engagement following the RDs.*

**Response:** We have revised this section to clearly explain the reasons behind participants’ desire for feedback. The revised text describes three factors: (1) having invested significant time and energy in the co-creation process, participants expected reciprocity and accountability regarding how their contributions would be used; (2) the gap between the final co-creation event and any tangible implementation created uncertainty about the impact of their participation, with some expressing frustration at the perceived silence following the workshops; and (3) participants who had begun sharing AMR messages in their communities needed ongoing support, resources, and validation to sustain their advocacy efforts. We also revised the framing to emphasize the importance of closing the feedback loop to maintain trust.

**Editor Comment 7 – Discussion: AMR as super-wicked problem and stakeholder analysis:** *AMR is a unique and super-wicked problem; needs stakeholder analysis (interests, alignment, power, actors, policy and their transformation potential). Authors need to acknowledge this preliminary engagement process before being able to first understand the actors, their interests and second sustain the engagement. Reference: PMID: 41125295.*

**Response:** We have added a new paragraph acknowledging AMR as a super-wicked problem, characterized by inherent complexity, multiple interrelated biological and social drivers, numerous stakeholders with competing interests, and limited time to act (Littmann et al., 2020). We discuss how addressing such problems requires not only participatory engagement but also systematic stakeholder analysis that maps the interests, alignment, power dynamics, and transformation potential of actors involved (Adhikari et al., 2025). We position the Malawi RDs as a preliminary but important step in a longer process, noting that sustained engagement requires first understanding who the key actors are, where their interests align or conflict, and how coalitions for sustained AMR action can be built. We use the exclusion of large-scale poultry farmers as an illustrative example of how incomplete stakeholder mapping can limit the reach of co-created solutions, and recommend that future iterations incorporate formal stakeholder analysis at the scoping phase.

# Reviewer #1

**R1 Overall Impression:** *The manuscript doesn’t explain the implementation of RDs through conversation events well. No mention of how many participants were in the RDs, what interventions were co-created, or how many CEs were conducted. Results focus on awareness/knowledge/attitudes; behaviour change feels like an afterthought. Conclusion not supported by results. Suggests refocusing on the RD process (co-creation, strengths, challenges, next steps).*

**Response:** We thank the reviewer for this detailed and constructive critique, which has led to substantial restructuring of the manuscript. We have made the following key changes: (1) Added implementation details in a new Methods subsection (“Study context: RDs implementation in Malawi”) specifying that two CEs were held for each of the three participant groups, the structure of co-creation workshops in Phase 3, and the national stakeholder meeting where outputs were presented. (2) The co-created solutions (community-led awareness campaigns, local antibiotic stewardship committees, integrating AMR education into existing health programs, promoting alternatives in livestock, and visual education materials) remain clearly presented in the Results. (3) Reframed the paper as a process evaluation rather than an effectiveness study, with the Discussion and Conclusion now focusing on process insights, implementation challenges, and practical lessons for future RDs implementation. (4) Revised the Conclusion to align with the evidence presented, focusing on what the RD process achieved, key challenges identified, and recommendations for future implementation.

**R1 Comment 1 – Abstract, Line 27: RDs abbreviation:** *Responsive Dialogues should include its corresponding abbreviation (RDs) after first mention. Remove abbreviation from Line 31.*

**Response:** Corrected. The abbreviation (RDs) is now introduced at the first mention of Responsive Dialogues in the abstract (Line 27): “The aim of Responsive Dialogues (RDs) is to engage communities...” The redundant abbreviation has been removed from Line 31.

**R1 Comment 2 – Introduction: WHO citation:** *The WHO citation links to an editorial (Kumar, 2021), not a WHO document. Please cite the relevant WHO document as a primary source and include the year.*

**Response:** We agree. We have replaced the Kumar (2021) editorial with the primary WHO source: World Health Organization. Global Action Plan on Antimicrobial Resistance. Geneva: WHO; 2015. This is now reference [1] in the revised manuscript.

**R1 Comment 3 – Introduction, Lines 75-84: Confusing structure:** *This section is confusing. Suggest starting with what RD is, then discuss how it was made iteratively in Thailand and Malawi, and position this as a pilot.*

**Response:** We have substantially restructured this section. The Introduction now contains only a concise overview of RDs: what they are (a participatory public engagement approach developed by Wellcome/ICARS), the purpose of the toolkit, that Malawi and Thailand were among the first to pilot it, key findings from the Thailand evaluation, and the aims of this study. The detailed implementation description (three phases, stakeholder mapping, CEs, COVID adaptations) has been moved to a new Methods subsection titled “Study context: RDs implementation in Malawi.” This addresses both the reviewer’s concern about clarity and Reviewer #2’s comment that Lines 75-102 read more like methodology.

**R1 Comment 4 – Introduction, Line 96: Remove “what the toolkit calls”:** *Remove “what the toolkit calls” so the sentence flows better.*

**Response:** Revised. In the new Methods subsection, the sentence now reads: “The second phase involved the implementation of RDs through CEs, day-long meetings where the identified stakeholders and participants together explored AMR topics and co-created solutions to address AMR.”

**R1 Comment 5 – Introduction, Line 96: Phase 2 not labelled:** *This is not labelled as the second phase, but phases one and three are explicitly labelled.*

**Response:** Corrected. Phase 2 is now explicitly labelled: “The second phase involved the implementation of RDs through CEs...” All three phases are now clearly delineated with separate paragraphs in the Methods subsection.

**R1 Comment 6 – Introduction, Line 99: COVID-19 adaptations not specified:** *The adaptations for COVID-19 are not specified.*

**Response:** We have specified the COVID-19 adaptations: “These adaptations included conducting CEs in well-ventilated spaces, implementing mask-wearing protocols, providing hand washing stations and hand sanitizer, and maintaining physical distancing between participants.”

**R1 Comment 7 – Introduction, Lines 100-102: Phase 3 lacks detail:** *Phase 2 and 3 should have own paragraphs. Phase 3 lacks contextual information – who was collating and synthesising learning?*

**Response:** Each phase now has its own paragraph in the Methods subsection. Phase 3 has been substantially expanded: “The third phase focused on synthesizing learnings from the first two phases through co-creation workshops. Each participant group attended a co-creation workshop where participants, together with the implementation team, invited community leaders, and members of the national AMR unit, synthesized findings from the CEs and co-created solutions and policy priorities. Following the group-specific workshops, a combined co-creation workshop brought together representatives from all three participant groups alongside community leaders, members of the national AMR unit, and other stakeholders to consolidate the co-created solutions and policy priorities. The outputs from this process were presented at a national stakeholder meeting attended by group representatives and members from the Ministry of Health and the Ministry of Agriculture.”

**R1 Comment 8 – Methods: Inconsistent abbreviations:** *Lines 113, 129, 131-132: Inconsistent use of abbreviations for CE, SSI, RD after introduction.*

**Response:** We have conducted a thorough review of the manuscript and standardized all abbreviations. SSIs is introduced at Line 113 and used consistently throughout (replacing “IDIs” and “key informant interviews”). CEs replaces “Conversation Events” after its introduction. RDs replaces “Responsive Dialogues” after first use. FGDs replaces “focus group discussions” after introduction.

**R1 Comment 9 – Methods, Lines 139-140: FGD locations and timelines:** *Please detail where FGDs were located and timelines.*

**Response:** We have expanded this section. SSIs were conducted where participants found most convenient, such as their homes or places of work such as pharmacies. FGDs were conducted at community halls. Each FGD lasted approximately 90-120 minutes as stated at Line 117-118.

**R1 Comment 10 – Methods, Lines 147-154: Confidentiality in FGDs:** *Please specify how confidentiality was kept in FGDs.*

**Response:** We have added the following to the Participants section: “For FGDs, ground rules were established at the beginning of each session, including an agreement among participants to maintain confidentiality about what was discussed. Identification numbers were given to each participant as identifiers to be used during transcription and analysis.”

**R1 Comment 11 – Methods, Line 156: Inconsistent interview terminology:** *You use Semi-structured interviews, SSI, interviews, and IDIs. Please decide on one universal term.*

**Response:** We have standardized to “semi-structured interviews (SSIs)” throughout the manuscript. The abbreviation is introduced at Line 113 and all subsequent references use SSIs, including replacing “IDIs” at Line 156.

**R1 Comment 12 – Methods, Lines 156-157: Recording, translation, quality checks:** *Were people told not to self-identify in interviews and that they were being recorded? Who translated? How many transcripts were randomly checked?*

**Response:** We have added: “Participants were informed that interviews would be audio-recorded as part of the consent process, and were advised to avoid using identifying information during recorded sessions.” In the Data management section, we now specify: “Transcription was conducted by RP, and translation into English by RP. Four translated transcripts were randomly checked by MP to ensure data accuracy.”

**R1 Comment 13 – Methods, Lines 161-164: Single coder:** *Only one person coded all transcripts? That is a bit unusual.*

**Response:** We appreciate this concern. First this was part of the corresponding author’s MSc project as such it was required to demonstrate research independence. However, while HS led the systematic coding of all transcripts using NVivo 12, the existing text at Lines 161-164 describes several quality assurance measures: initial coding was reviewed by DN; developed codes and themes were discussed and refined by team members MP, JM, RP, and EM; and consultation sessions were held with all researchers. This iterative team-based approach to code review and theme development ensured analytical rigor despite the primary coding being conducted by one researcher. We have also added a reflexivity paragraph to the Limitations acknowledging the positionality of HS as the lead coder and researcher.

**R1 Comment 14 – Results, Line 214: Missing comma:** *Need a comma after “farmers.”*

**Response:** Corrected. The text now reads: “...male caregivers and farmers, stated that...”

**R1 Comment 15 – Results, Lines 216-218: Interpretive sentence:** *This sentence is more of a conclusion/discussion than a result. Suggest moving.*

**Response:** Agreed. The sentence “This reported shift in attitude may represent a significant outcome of the RDs, suggesting that improving understanding of AMR could potentially lead to more responsible antibiotic use practices at the community level” has been removed from the Results. The point is now addressed in the revised Discussion.

**R1 Comment 16 – Results, Lines 226-228: Discussion sentence in results:** *This is a discussion sentence, not results.*

**Response:** Agreed. The sentence “This reported change in behaviour among healthcare professionals is noteworthy, as it suggests that the RDs may have been effective in influencing not only community members but also those who play a crucial role in antibiotic stewardship” has been removed from the Results. This point is addressed in the revised Conclusion.

**R1 Comment 17 – Results, Lines 232-241: Concrete behaviour change examples:** *There are reported intentions but did anyone give examples of discussing with neighbors?*

**Response:** We acknowledge that the data primarily captured reported intentions to share knowledge and educate others, rather than verified examples of actual community-level dissemination. We have been more careful throughout the Results to distinguish between reported intentions and reported actual behaviour changes. We have also strengthened the Limitations section to acknowledge that self-reported data is subject to social desirability bias and that the absence of baseline data limits our ability to track actual behaviour change.

**R1 Comment 18 – Discussion, Lines 369-372: Thailand vs Malawi comparison:** *Thailand saw spontaneous AMR initiatives. Discuss why this didn’t happen in Malawi. Would RDs be less feasible in similar countries?*

**Response:** We have substantially expanded this comparison. The revised Discussion now analyses contextual differences between the two settings: Thailand’s relatively more developed healthcare infrastructure and stronger regulatory frameworks; greater resource availability for community-level action; differences in existing community organization structures; and the disruptive impact of COVID-19 on the Malawi pilot. We explicitly state that the absence of spontaneous initiatives does not mean RDs are less feasible in countries similar to Malawi, but rather that additional support structures, sustained engagement, and dedicated resource allocation are essential to translate dialogue outcomes into community action in resource-constrained settings.

**R1 Comment 19 – Discussion: Missing large poultry farmers:** *Include insights about not including large poultry farmers and how this population was missed.*

**Response:** This is addressed in the Results (existing text at Lines 313-324) and is now further discussed in the new super-wicked problem paragraph in the Discussion. We use the exclusion of large-scale poultry farmers as an illustrative example of how incomplete stakeholder mapping can limit the reach and effectiveness of co-created solutions, and recommend that future iterations incorporate formal stakeholder analysis at the scoping phase to ensure all relevant actors across the One Health spectrum are systematically identified and engaged.

# Reviewer #2

**R2 Comment 1 – Title: Misleading:** *The title may be misleading. Did you co-create solutions through a qualitative evaluation, or evaluate the implementation? No framework/model/list of co-created solutions presented.*

**Response:** We agree with this important distinction. The study evaluated the implementation of the AMR RDs in Malawi. We have revised the title as suggested by the editor to “Co-creation of community-driven solutions and policy priorities to address antimicrobial resistance through Responsive Dialogues: A qualitative evaluation from Malawi.” The co-created solutions are clearly presented in the Results section: community-led awareness campaigns on responsible antibiotic use, local antibiotic stewardship committees, integrating AMR education into existing community health programs, promoting alternative medicine to reduce antibiotic use in livestock, and visual education materials (Figure 1). The revised framing throughout the manuscript is now consistent with a process evaluation.

**R2 Comment 2 – Abstract, Line 26: Reword:** *Reword: “Inappropriate antibiotic use due to low public knowledge and awareness.”*

**Response:** Revised. The opening line of the abstract now reads: “Inappropriate antibiotic use due to low public knowledge and awareness contributes to the growing Antimicrobial Resistance (AMR) burden, especially in low- and middle-income countries (LMICs).”

**R2 Comment 3 – Abstract, Line 27: Use abbreviation:** *Use Responsive Dialogues (RD).*

**Response:** We have introduced the abbreviation at the first mention: “The aim of Responsive Dialogues (RDs) is to engage communities...” We use RDs (plural) throughout for consistency.

**R2 Comment 4 – Introduction, Lines 54-55: Add reference:** *Add reference.*

**Response:** The statements at Lines 54-55 regarding AMR’s clinical and economic consequences in Malawi are supported by references [4,5] (Lester et al., 2022, 2023). We have verified the citations are correctly positioned.

**R2 Comment 5 – Introduction, Line 74: Abrupt subtitle:** *Abrupt subtitle.*

**Response:** We have addressed this in two ways. First, we added a transitional sentence at the end of the preceding paragraph: “However, there is limited empirical evidence on how participatory approaches function in practice in resource-limited settings, particularly for addressing complex multi-sectoral challenges like AMR.” Second, the detailed content that was under this subtitle has been moved to the Methods section, and the Introduction now contains a concise RDs overview that flows naturally from the transitional sentence without requiring a separate subtitle.

**R2 Comment 6 – Introduction, Lines 75-102: Sounds like methodology:** *Sounds more like methodology; could go to the methods section or supplementary.*

**Response:** Agreed. We have moved the detailed implementation description (the three phases, stakeholder mapping, CEs, COVID adaptations) to a new Methods subsection titled “Study context: RDs implementation in Malawi,” placed before the existing “Data collection” subsection. The Introduction now retains only a brief overview of the RDs approach and the aims of this study.

**R2 Comment 7 – Methods: Quantify as “AMR RD”:** *There is need to quantify that the RD was an “AMR RD” for rigor (transparency).*

**Response:** We have ensured that throughout the Methods section, the RDs are consistently referred to as “AMR RDs” to make it explicit that the dialogues were specifically focused on antimicrobial resistance.

**R2 Comment 8 – Results, Lines 168, 172: Use abbreviations:** *Shorthand already used for RDs, FGDs, KIIs.*

**Response:** Corrected. All abbreviated terms are now consistently used after their first introduction. “Responsive Dialogues” has been replaced with “RDs,” “focus group discussions” with “FGDs,” and “key informant interviews” with “SSIs” (as we have standardized to semi-structured interviews per Reviewer #1’s suggestion).

**R2 Comment 9 – Discussion, Line 353: Feasibility vs evaluation:** *What exactly did this study evaluate? Evaluation is after implementation assuming feasibility was already determined.*

**Response:** We have clarified the opening of the Discussion. The revised sentence reads: “This study evaluated the process of implementing AMR RDs in Malawi, examining how participants experienced the engagement, what solutions they co-created, and what challenges they identified in implementation.” This makes clear that the study was a process evaluation rather than a feasibility assessment.

**R2 Comment 10 – Discussion, Line 356: Paragraph flow:** *The following paragraph doesn’t fit with the previous paragraph.*

**Response:** We have improved the transition between paragraphs. The revised opening paragraph now ends with: “Below, we discuss these findings in relation to the existing literature,” which creates a clear bridge to the subsequent discussion of AMR awareness and existing evidence.

**R2 Comment 11 – Discussion: Concordance/discordance with literature:** *After Line 356, paragraphs need major revisions with concordance and discordance with current literature on AMR RDs.*

**Response:** We have substantially revised the Discussion to more systematically compare our findings with existing literature. Key additions include: (a) an expanded comparison with the Thailand RDs pilot (Poomchaichote et al., 2024) identifying both concordant outcomes (improved awareness, co-creation of solutions) and discordant findings (spontaneous community action in Thailand but not Malawi), with analysis of contextual factors explaining the differences; (b) new discussion of structural barriers drawing on ethical tensions literature (Adhikari et al., 2021, 2024); (c) positioning of findings within the super-wicked problem framework (Littmann et al., 2020) and stakeholder analysis literature (Adhikari et al., 2025).

**R2 Comment 12 – Limitations: Reflexivity and triangulation:** *Consider adding something on reflexivity and triangulation of researchers.*

**Response:** We have added a new paragraph to the Limitations section. Regarding reflexivity: we describe how the lead researcher (HS) is a Chichewa-speaking Malawian researcher with extensive experience in qualitative health research in the study communities and was involved in the broader RDs implementation. We acknowledge that this insider perspective facilitated rapport and cultural understanding but may also have influenced participant responses and data interpretation, and that regular reflexive discussions within the research team helped mitigate potential biases. Regarding triangulation: we describe data triangulation through multiple methods (SSIs and FGDs) and multiple participant groups, and investigator triangulation through collaborative coding review and theme development involving multiple team members (DN, MP, JM, RP, EM). We acknowledge that methodological triangulation (e.g., incorporating observational data from the CEs) would have further strengthened the findings.

**R2 Comment 13 – Conclusions: Amend accordingly:** *Amend accordingly.*

**Response:** The Conclusion has been fully rewritten to align with the process evaluation framing. The revised conclusion focuses on: (1) what the RD process achieved in terms of engagement, collaborative learning, improved awareness, and co-creation of solutions; (2) critical challenges including structural barriers, absence of feedback mechanisms, and incomplete stakeholder mapping; (3) practical recommendations for future implementation including systematic stakeholder analysis, resource allocation for post-dialogue follow-up, integration with health system structures and national AMR action plans, and feedback mechanisms; and (4) future research priorities including long-term impact assessment and combining participatory approaches with structural interventions.

# Data Availability

**Reviewer #1 flagged data availability as “No”:**

**Response:** We have revised the Data Availability Statement to be more specific about what data are available and how researchers can request access. The statement now specifies that relevant data excerpts are included within the manuscript, the SSI and FGD guides are provided as supporting information (S1 File and S2 File), and that researchers wishing to access additional data may submit a request to the Malawi Liverpool Wellcome Programme Community Health Sciences Research Support Unit at crsu@mlw.mw, with requests reviewed in accordance with ethical approval conditions and institutional data sharing policies.

We believe these revisions have substantially strengthened the manuscript and we hope it is now suitable for publication in PLOS Global Public Health.

Sincerely,

Henry Sambakunsi, on behalf of all authors
